# Supplementary material for: Zinc as adjunct treatment for clinical severe infection in young infants: A randomized double-blind placebo-controlled trial in India and Nepal
Source: PLoS Med. 2025 Oct 9;22(10):e1004759. doi: 10.1371/journal.pmed.1004759 (PMC12527131; doi:10.1371/journal.pmed.1004759)
Supplement: S1 Appendix — This appendix provides the names, affiliations, and highest degrees of the members of the Zinc Sepsis Study Group, listed in alphabetical order. (DOCX) [file pmed.1004759.s001.docx]

# **S1 Appendix: Zinc sepsis study group**

| **S No** | **Name**  ***(alphabetical order)*** | **Affiliation** | **Highest Degree** |
| --- | --- | --- | --- |
|  | Dr Ajay Kumar (AJ) | Vardhman Mahavir Medical College & Safdarjung Hospital, New Delhi, India | MD |
|  | Dr Ajay Kumar (AK) | Maulana Azad Medical College, New Delhi, India | MD |
|  | Dr Anju Seth (AS) | Kalawati Sharan Children's Hospital, Delhi, India | MD |
|  | Dr Anuradha Govil (AG) | Kasturba Hospital, Delhi, India | MD |
|  | Dr Antara Sinha (AN) | Translational Health Science and Technology Institute, Faridabad, India | MD |
|  | Dr Anup Mohta (AM) | Chacha Nehru Bal Chikitsalaya, Delhi, India | MS, MCh |
|  | Ms Ayushi (AA) | Translational Health Science and Technology Institute, Faridabad, India | MSc |
|  | Dr Debjani Ram Purakayastha (DRP) | Indian Council of Medical Research, New Delhi, India | PhD |
|  | Mr Dharmendra Sharma (DS) | Translational Health Science and Technology Institute, Faridabad, India | MCA |
|  | Dr Ganesh Prasad Shah (GS) | Patan Academy of Health Sciences, Lalitpur, Nepal | MD |
|  | Dr Ganesh Rai (GR) | Kathmandu Medical College, Kathmandu University, Nepal | MD |
|  | Dr Halvor Sommerfelt (HS) | Centre for Intervention Science in Maternal and Child Health (CISMAC) and Centre for International Health, University of Bergen, Norway | MD, PhD |
|  | Dr Harish Chellani (HC) | Vardhman Mahavir Medical College & Safdarjung Hospital, New Delhi, India | MD |
|  | Dr Harish K. Pemde (HP) | Kalawati Sharan Children's Hospital, Delhi, India | MD |
|  | Dr Kanika Kapoor (KK) | Vardhman Mahavir Medical College & Safdarjung Hospital, New Delhi, India | MD |
|  | Dr Kirtisudha Mishra (KM) | Chacha Nehru Bal Chikitsalaya, Delhi, India | MD |
|  | Dr Laxman Prasad Shrestha (LS) | Institute of Medicine, Tribhuvan University, Kathmandu, Nepal | MD |
|  | Dr Mamta Jajoo (MJ) | Chacha Nehru Bal Chikitsalaya, Delhi, India | MD |
|  | Dr Manish Kumar (MK) | Chacha Nehru Bal Chikitsalaya, Delhi, India | MD |
|  | Dr Medha Mittal (MM) | Chacha Nehru Bal Chikitsalaya, Delhi, India | MD |
|  | Dr NB Mathur (NB) | Maulana Azad Medical College, New Delhi, India | MD |
|  | Dr Nitya Wadhwa (NW) | Translational Health Science and Technology Institute, Faridabad, India | MD |
|  | Dr Raghvendra Singh (RS) | Maulana Azad Medical College, New Delhi, India | MD |
|  | Dr Ram Hari Chapagain (RHC) | Kanti Children’s Hospital & National Academy of Medical Sciences, Kathmandu, Nepal | MD |
|  | Dr Rani Gera (RG) | Vardhman Mahavir Medical College & Safdarjung Hospital, New Delhi, India | MD |
|  | Dr Ratan Gupta (RA) | Vardhman Mahavir Medical College & Safdarjung Hospital, New Delhi, India | MD |
|  | Dr Shinjini Bhatnagar (SB) | Translational Health Science and Technology Institute, Faridabad, India | PhD |
|  | Dr Srijana Basnet (SI) | Institute of Medicine, Tribhuvan University, Kathmandu, Nepal | MD |
|  | Dr Suchita Shrestha (SS) | Oxford University Clinical Research Unit, Patan Academy of Health Sciences, Lalitpur, Nepal | MPH |
|  | Dr Sudha Basnet (SU) | Institute of Medicine, Tribhuvan University, Kathmandu, Nepal | MD, PhD |
|  | Dr Sugandha Arya (SA) | Vardhman Mahavir Medical College & Safdarjung Hospital, New Delhi, India | MD |
|  | Dr Sunita Bhatia (SN) | Kasturba Hospital, Delhi, India | MD |
|  | Dr Tor A. Strand (TAS) | Centre for Intervention Science in Maternal and Child Health and Centre for International Health, University of Bergen, Bergen, Norway  Department of Research, Innlandet Hospital Trust, Lillehammer, Norway | MD, PhD |
|  | Dr Uma Chandra Mouli Natchu | Society for Applied Studies, Centre for Health Research and Development, India | MD |
|  | Dr Urmila Jhamb (UJ) | Maulana Azad Medical College, New Delhi, India | MD |
|  | Dr Virendra Kumar (VK) | Kalawati Sharan Children's Hospital, Delhi, India | MD |
|  | Dr Varinder Singh (VS) | Kalawati Sharan Children's Hospital, Delhi, India | MD |
