# Supplementary material for: Zinc as adjunct treatment for clinical severe infection in young infants: A randomized double-blind placebo-controlled trial in India and Nepal
Source: PLoS Med. 2025 Oct 9;22(10):e1004759. doi: 10.1371/journal.pmed.1004759 (PMC12527131; doi:10.1371/journal.pmed.1004759)
Supplement: S2 Checklist — This file documents the extenuating circumstances, important modifications, impacts, and mitigating strategies applied during the trial in response to the COVID-19 pandemic, following the CONSERVE reporting extension. (DOCX) [file pmed.1004759.s003.docx]

# CONSERVE Checklists

Use CONSERVE-CONSORT for completed trial reports and CONSERVE-SPIRIT for trial protocols.

| CONSERVE-CONSORT Extension: [DATE] | | | | | |
| --- | --- | --- | --- | --- | --- |
| Item | Item Title | Description | | | Page No. |
| I. | Extenuating Circumstances | Describe the circumstances and how they constitute extenuating circumstances. | | | The COVID-19 pandemic; Discussion section (para 4, page 22, lines 404–406) |
| II. | Important Modifications | 1. Describe how the modifications are important modifications. | | | The pandemic posed serious challenges to the implementation of our trial, as most sites had to temporarily stop recruitment, which limited the number of total infants enrolled (Discussion section, para 4, page 22, lines 404–406) |
|  |  | 1. Describe the impacts and mitigating strategies, including their rationale and implications for the trial. | | | Most sites had to temporarily stop recruitment during COVID pandemic. This limited the number of eligible children leading to a shortfall in achieving the projected sample size.  (Discussion section, para 4, page 22, lines 404–406) |
|  |  | 1. Provide a modification timeline. | | | Screening and recruitments were stopped on 23 March 2020; Resumption of screening and recruitments was phased across hospitals from 27 May 2020 to 15 Feb 2021 |
| III. | Responsible Parties | State who planned, reviewed and approved the modifications. | | | The modifications were planned by investigator consensus and approved by the institutional ethics committees. |
| IV. | Interim data | If modifications were informed by trial data, describe how the interim data were used, including whether they were examined by study group, and whether the individuals reviewing the data were blinded to the treatment allocation. | | | NA |
| CONSORT Number and Item | | For each row, if important modifications occurred check “direct impact” and/or “mitigating strategy” and describe the changes in the trial manuscript or supplement. Check “no change” for items that are unaffected in the extenuating circumstance. | | | Page No. |
|  |  | No Change | Impact* | Mitigating Strategy** |  |
| 1 | Title and abstract | **√** |  |  |  |
| 2 | Introduction | **√** |  |  |  |
| 3 | Methods: Trial Design | **√** |  |  |  |
| 4 | Methods: Participants | **√** |  |  |  |
| 5 | Methods: Interventions | **√** |  |  |  |
| 6 | Methods: Outcomes | **√** |  |  |  |
| 7 | Methods: Sample Size | **√** |  |  |  |
| 8-10 | Methods: Randomisation | **√** |  |  |  |
| 11 | Methods: Blinding | **√** |  |  |  |
| 12 | Methods: Statistical methods | **√** |  |  |  |
| 13 | Results: Participant flow | **√** |  |  |  |
| 14 | Results: Recruitment | **√** |  |  |  |
| 15 | Results: Baseline data | **√** |  |  |  |
| 16 | Results: Numbers analysed | **√** |  |  |  |
| 17 | Results: Outcomes and estimation | **√** |  |  |  |
| 18 | Results: Ancillary analyses | **√** |  |  |  |
| 19 | Results: Harms | **√** |  |  |  |
| 20 | Discussion: Limitations |  | **√** |  | Most sites had to temporarily stop recruitment during COVID pandemic. This limited the number of eligible children leading to a shortfall in achieving the projected sample size. **(**Discussion section, para 4, page 22, lines 404–406) |
| 21 | Discussion: Generalisability | **√** |  |  |  |
| 23 | Other information: Registration | **√** |  |  |  |
| 24 | Other information: Protocol | **√** |  |  |  |
| 25 | Other information: Funding | **√** |  |  |  |
| *Aspects of the trial that are directly affected or changed by the extenuating circumstance and are not under the control of investigators, sponsor or funder.  **Aspects of the trial that are modified by the study investigators, sponsor or funder to respond to the extenuating circumstance or manage the direct impacts on the trial. | | | | | |

| CONSERVE-SPIRIT Extension: [DATE] | | | | | |
| --- | --- | --- | --- | --- | --- |
| Item | Item Title | Description | | | Page No. |
| I. | Extenuating Circumstances | Describe the circumstances and how they constitute extenuating circumstances. | | |  |
| II. | Important Modifications | 1. Describe how the modifications are important modifications. | | |  |
|  |  | 1. Describe the impacts and mitigating strategies, including their rationale and implications for the trial. | | | (see below) |
|  |  | 1. Provide a modification timeline. | | |  |
| III. | Responsible Parties | State who planned, reviewed and approved the modifications. | | |  |
| IV. | Interim data | If modifications were informed by trial data, describe how the interim data were used, including whether they were examined by study group, and whether the individuals reviewing the data were blinded to the treatment allocation. | | |  |
| SPIRIT Item and Number | | For each row, if important modifications occurred, check one or both of “impact” and/or “mitigating strategy” and describe the changes in the protocol. Check “no change” for items that are unaffected in the extenuating circumstance. | | | Page No. |
|  |  | No Change | Impact* | Mitigating Strategy** |  |
| 1 | Title |  |  |  |  |
| 2 | Trial registration |  |  |  |  |
| 3 | Protocol version |  |  |  |  |
| 4 | Funding |  |  |  |  |
| 5 | Roles and responsibilities |  |  |  |  |
| 6 | Background and rationale |  |  |  |  |
| 7 | Objectives |  |  |  |  |
| 8 | Trial design |  |  |  |  |
| 9 | Study setting |  |  |  |  |
| 10 | Eligibility criteria |  |  |  |  |
| 11 | Interventions |  |  |  |  |
| 12 | Outcomes |  |  |  |  |
| 13 | Participant timeline |  |  |  |  |
| 14 | Sample size |  |  |  |  |
| 15 | Recruitment |  |  |  |  |
| 16 | Allocation |  |  |  |  |
| 17 | Blinding (masking) |  |  |  |  |
| 18 | Data collection methods |  |  |  |  |
| 19 | Data management |  |  |  |  |
| 20 | Statistical methods |  |  |  |  |
| 21 | Data monitoring |  |  |  |  |
| 22 | Harms |  |  |  |  |
| 23 | Auditing |  |  |  |  |
| 24 | Research ethics approval |  |  |  |  |
| 25 | Protocol amendments |  |  |  |  |
| 26 | Consent or assent |  |  |  |  |
| 27 | Confidentiality |  |  |  |  |
| 28 | Declaration of interests |  |  |  |  |
| 29 | Access to data |  |  |  |  |
| 30 | Ancillary and post-trial care |  |  |  |  |
| 31 | Dissemination policy |  |  |  |  |
| 32 | Informed consent materials |  |  |  |  |
| 33 | Biological specimens |  |  |  |  |
| *Aspects of the trial that are directly affected or changed by the extenuating circumstance and are not under the control of investigators, sponsor or funder.  **Aspects of the trial that are modified by the study investigators, sponsor or funder to respond to the extenuating circumstance or manage the direct impacts on the trial. | | | | | |
