# Supplementary material for: Zinc as adjunct treatment for clinical severe infection in young infants: A randomized double-blind placebo-controlled trial in India and Nepal
Source: PLoS Med. 2025 Oct 9;22(10):e1004759. doi: 10.1371/journal.pmed.1004759 (PMC12527131; doi:10.1371/journal.pmed.1004759)
Supplement: S1 Table — (DOCX) [file pmed.1004759.s004.docx]

**S1 Table: Reasons for losses to follow-up during the 12-week study period**

|  | **Zinc^a^**  **n=1,576** | **Placebo^a^**  **n=1,577** |
| --- | --- | --- |
| Lost to follow-up | 22 (1.40%) | 27 (1.71%) |
| Reasons |  |  |
| 1. Consent for participation withdrawn | 2 (0.32%) | 2 (0.13%) |
| 2. LAMA^b^/ absconded from hospital & lost to follow-up | 5 (0.19%) | 3 (0.19%) |
| 3. Discharged but lost to follow-up after that | 15 (0.95%) | 22 (1.40%) |

**^a^** All values are n (%)

^b^LAMA: Left against medical advice
