# Supplementary material for: Zinc as adjunct treatment for clinical severe infection in young infants: A randomized double-blind placebo-controlled trial in India and Nepal
Source: PLoS Med. 2025 Oct 9;22(10):e1004759. doi: 10.1371/journal.pmed.1004759 (PMC12527131; doi:10.1371/journal.pmed.1004759)
Supplement: S2 Table — (DOCX) [file pmed.1004759.s005.docx]

**S2 Table: Compliance to adjunct zinc or placebo**

|  | **Zinc^a^**  **n=1,576** | **Placebo^a^**  **n=1,577** |
| --- | --- | --- |
| Time to initiation of study intervention (in minutes), median (IQR) | 18 (9–32) | 17 (9–31) |
| Study intervention never initiated | 3 (0.2%) | NA |
| Received at least 50% of intervention doses in first 5d | 1,374 (87%) | 1,354 (86%) |
| Completed 14 days of study intervention | 1,494/1,506 (99.2%) | 1,481/1,495 (99.1%) |

^a^ All values are n (%) except where specified
