# Supplementary material for: Zinc as adjunct treatment for clinical severe infection in young infants: A randomized double-blind placebo-controlled trial in India and Nepal
Source: PLoS Med. 2025 Oct 9;22(10):e1004759. doi: 10.1371/journal.pmed.1004759 (PMC12527131; doi:10.1371/journal.pmed.1004759)
Supplement: S3 Table — Plasma zinc concentration measured at enrollment and at discharge in a subset of infants from Kalawati Saran Children’s hospital. The data underlying the values shown in this table can be found in S1 Data. (DOCX) [file pmed.1004759.s006.docx]

**S3 Table: Plasma zinc in the two treatment arms; change in zinc concentration from enrollment to discharge**

|  | **Zinc^a, b^** | **Placebo^a, b^** | **Mean Difference (95% CI)** |
| --- | --- | --- | --- |
| Plasma zinc concentration at enrollment (μmol/L) | n=36  10.1 (4.8) | n=36  10.3 (4.1) | NA |
| Plasma zinc concentration at discharge (μmol/L) | n=30  12.3 (3.1) | n=26  10.4 (4.0) | NA |
| Change in zinc concentration from enrollment to discharge (μmol/L) | n=30  2.2 (6.2) | n=24  0.3 (5.1) | 1.9 (-1.3, 5.0) |

^a^ All values are Mean (SD) except where specified

^b^ Plasma zinc concentration measured at enrollment and at discharge in a subset of infants from Kalawati Saran Children’s hospital
