# Supplementary material for: Zinc as adjunct treatment for clinical severe infection in young infants: A randomized double-blind placebo-controlled trial in India and Nepal
Source: PLoS Med. 2025 Oct 9;22(10):e1004759. doi: 10.1371/journal.pmed.1004759 (PMC12527131; doi:10.1371/journal.pmed.1004759)
Supplement: S4 Table — (DOCX) [file pmed.1004759.s007.docx]

**S4 Table: Adverse events in young infants with clinical severe infection**

| **Adverse events^b^** | **Zinc^a^**  **n=1,576** | **Placebo^a^**  **n=1,577** | **RD (95% CI)** |
| --- | --- | --- | --- |
| Vomiting related to the time of intervention: |  |  |  |
| Vomited the first dose of intervention within 30 minutes | 21/1,573 (1.33%) | 7/1,577 (0.44%) | -0.89 (-1.54, -0.23) |
| Vomited any subsequent intervention within 30 minutes | 40/1,573 (2.54%) | 37/1,577 (2.35%) | -0.19 (-1.27, 0.89) |
| Vomited once or more times during the 14-day treatment period unrelated to time of intervention | 164 (10.41%) | 143 (9.07%) | -1.34 (-3.41, 0.73) |
| Abdominal distension lasting > 24 hours at any time during the 14-day treatment period | 19 (1.21%) | 15 (0.95%) | -0.25 (-0.98, 0.47) |

^a^ All values are n (%) except where specified

^b^ n= Number of infants who received at least one dose of the intervention
