# Supplementary material for: Zinc as adjunct treatment for clinical severe infection in young infants: A randomized double-blind placebo-controlled trial in India and Nepal
Source: PLoS Med. 2025 Oct 9;22(10):e1004759. doi: 10.1371/journal.pmed.1004759 (PMC12527131; doi:10.1371/journal.pmed.1004759)
Supplement: S1 Fig — (DOCX) [file pmed.1004759.s008.docx]

**S1 Fig: Prespecified subgroup analysis of treatment failure**


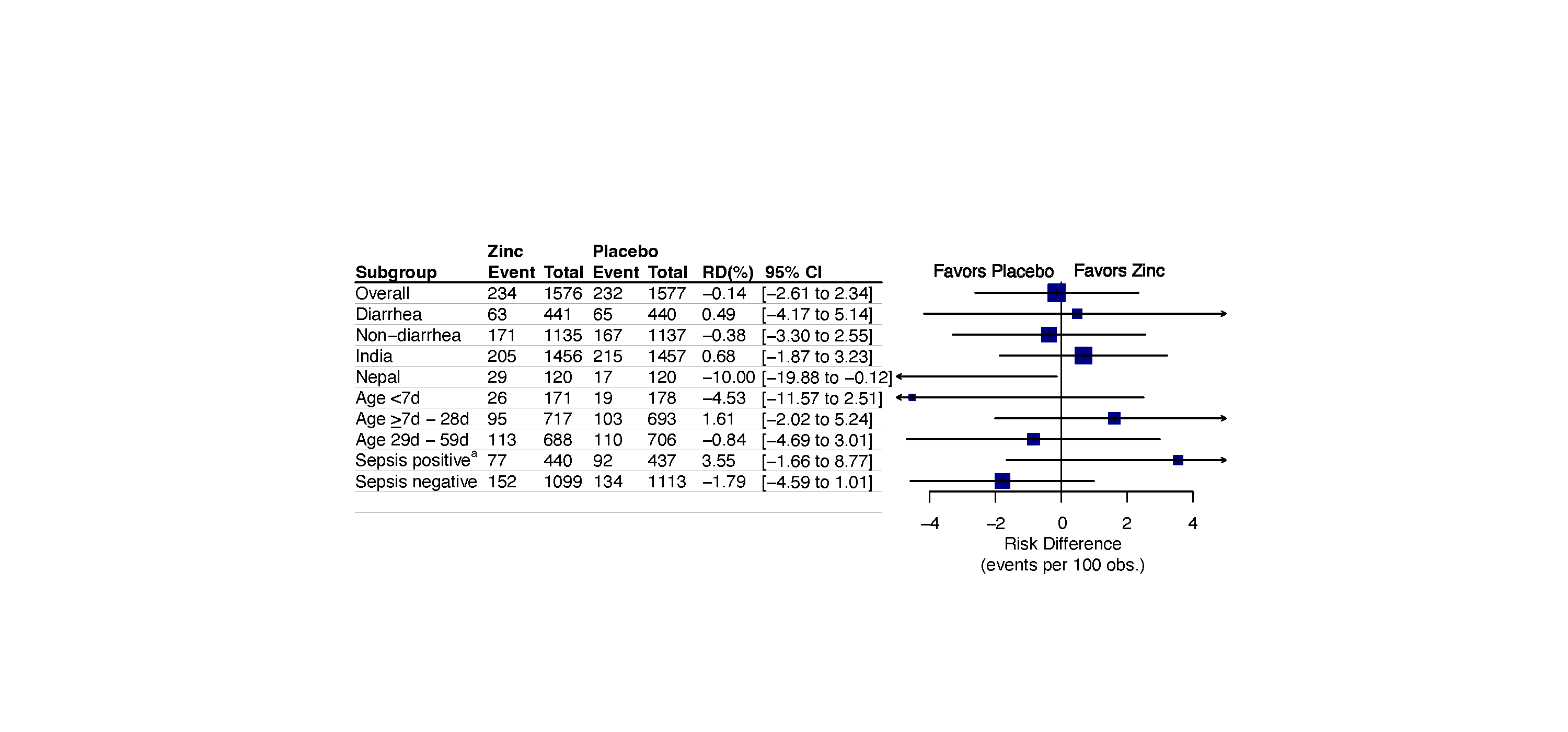


^a^ Presence of either: i) positive blood culture with potentially pathogenic bacteria, or ii) positive septic screen, indicated by any two or more of the following laboratory parameters: total leucocyte count <5 x 10^9^ cells/L; absolute neutrophil count <1.5 x 10^9^ cells/L; band cell to neutrophil ratio >0.2; micro ESR >15mm in the first hour; and C-reactive protein levels >10 mg/L.
