# Supplementary figures and images for: Zinc as adjunct treatment for clinical severe infection in young infants: A randomized double-blind placebo-controlled trial in India and Nepal
Source: PLoS Med. 2025 Oct 9;22(10):e1004759. doi: 10.1371/journal.pmed.1004759 (PMC12527131; doi:10.1371/journal.pmed.1004759)

# **S2 Fig: Post-hoc subgroup analysis of treatment failure**


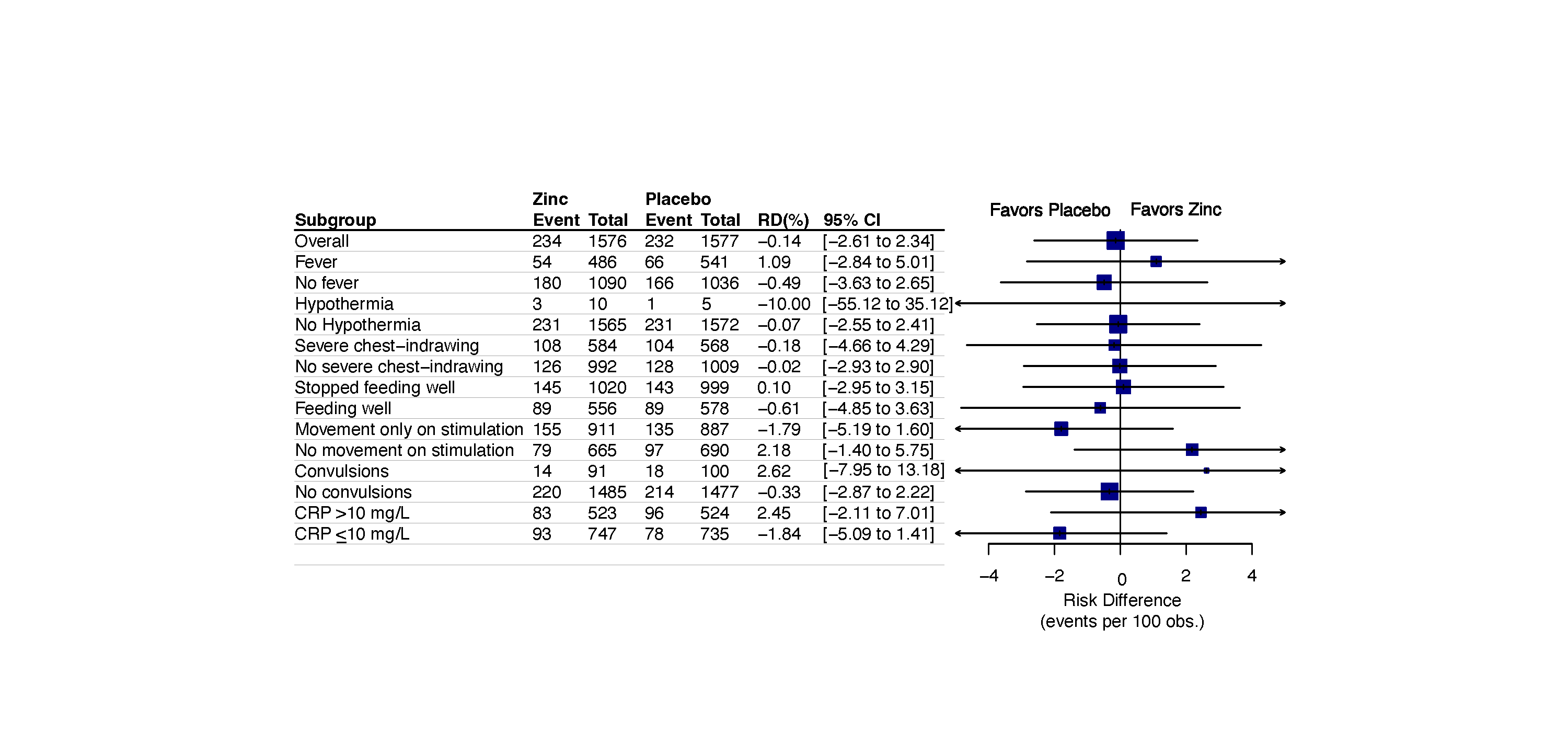

Supplement: S2 Fig — (DOCX) [file pmed.1004759.s009.docx]
