# Supplementary material for: Zinc as adjunct treatment for clinical severe infection in young infants: A randomized double-blind placebo-controlled trial in India and Nepal
Source: PLoS Med. 2025 Oct 9;22(10):e1004759. doi: 10.1371/journal.pmed.1004759 (PMC12527131; doi:10.1371/journal.pmed.1004759)
Supplement: S1 Protocol — This file provides the full trial protocol, including study rationale, objectives, design, eligibility criteria, interventions, outcomes, statistical analysis plan, and operational procedures. (PDF) [file pmed.1004759.s011.pdf]

Letter of Amendment #1

Date: June 09, 2016

Re: Letter of Amendment #1 for Protocol No. RCN/ZINCSEVINF/02/2015 Version 1.0 dated March 4, 2016

Title: Zinc as an adjunct for the treatment of clinical severe infection in infants younger than 2 months

To: Institutional Ethics Committees participating in and reviewing Protocol No. RCN/ZINCSEVINF/02/2015, Site Investigators

From: Dr. Nitya Wadhwa, Principal Investigator

The following information impacts the RCN/ZINCSEVINF/02/2015 study and must be forwarded to concerned Institutional Ethics Committee (IEC) as soon as possible for their information and review. This must be notified to the THSTI IEC and the respective hospital site IECs before implementation.

Upon notifying the THSTI IEC and the respective hospital site IECs of this LoA, The amended protocol will be considered the updated final protocol to be followed at the sites.

The following modifications are made to Protocol No. RCN/ZINCSEVINF/02/2015 Version 1.0 dated March 4, 2016.

S No	Protocol No. RCN/ZINCSEVINF/02/2015 version 1.0 dated March 4, 2016	Section/ page no in protocol no. RCN/ZINCSEVINF/02/2015 version 1.0 dated March 4, 2016	Revision in protocol no. RCN/ZINCSEVINF/02/2015 version 1.1 dated June 09, 2016	Reason for change
1.	Clarification between objective and outcome of study	i. Trial synopsis: objectives of study, page 11<br>ii. Section 3, Study objectives, page 20	The sections on study objectives and study outcomes have been revised such that there is a clear demarcation and clarity between study objectives and outcomes	In response to comment by IEC THSTI

		iii. Section 3.3, outcomes, pages 21 & 22<br>iv. Section 5.5.4, outcomes, pages 31 & 32		
2.	Study on viral etiology is not defined well. This needs to be removed along with the associated protocol of taking nasal swabs	i. Trial synopsis: Biospecimen collection, page 12<br>ii. Laboratory procedures, pages 35-36<br>iii. Investigation Checklist, page 37	The sections on viral etiology and collection of nasal swab have been removed	In response to comment by IEC THSTI
3.	The study flow has been modified	i. Figure 1, page 24<br>ii. Eligibility Criteria, pages 28-29	Modified study flow: Infant coming to emergency services will be first screened for inclusion criteria, next any exclusion criteria will be ruled out; following this we will look to see if infant requires any stabilization. If infant has inclusion, no exclusion and is stabilized, written informed consent taken from parent (s)/ guardian will be taken and then infant enrolled. objectives and study outcomes have been revised such that there is a clear demarcation and clarity between study objectives and outcomes	Consensus of site investigators and PI
4.	Clarity on the follow-up of the	i. Figure 1: page 24	At discharge, the parent/	Consensus of site investigators and PI

	enrolled infants post discharge	ii. Section 5.5.5.c. Clinical Data Collection, pages 33-34<br>iii. Section 10.2.2. Selection of subjects to be included in the analysis, page 49	Guardian will be asked to come to the hospital for a follow-up after completion of the 14 day course of the intervention, around day 15. Following this there will be two telephonic follow-ups at 6 weeks and 12 weeks after the day of enrolment.	
5.	Adverse event and serious adverse event reporting	Section 8.0, page 41-45	A Serious Adverse Event (study specific) for the enrolled and hospitalized infant is defined as any untoward medical occurrence in the enrolled participant, that at any dose:<br>i. Results in death anytime during the study period<br>ii. Is life threatening (defined as a participant at immediate risk of death at the time of the event). For eg Initiation of life support<br>iii. Requires prolongation of existing hospital admission Prolongation of hospital admission during the study period can be due to<br>a) reappearance of signs of clinical severe infection after a 48 hours symptom free period<br>b) development of signs of critical illness where life	Consensus of site investigators and PI

			support is required like apnea, shock with low pulse volume and/ or delayed capillary refill time<br>c) development of necrotizing enterocolitis<br>d) appearance of new symptom or any unexpected event which could lead to prolongation of hospital stay like injection abscess or fall from cot leading to fracture or head injury<br><br>A Serious Adverse Event (study specific) for the enrolled infant who has been discharged from hospital but continues to be a part of the study till completion of the study duration (which is upto 12 weeks from day of enrolment is still in the study till 12 weeks from day of enrolment) is defined as any untoward medical occurrence in the enrolled participant, that results in:<br>i. Death anytime during the study period<br>ii. Serious illness leading to hospitalization	

6.	Ethics approvals	i. Section 9.2, page 45<br>ii. Section 13, page 54	The Investigators will initiate the study at each site only after securing approval from the Institutional Ethics Committees of the hospital in which the study is to be carried out as well as from THSTI IEC and REC West in Norway for the Indian sites and likewise from NHRC Nepal and REC West in Norway for the Nepal sites.	Consensus of site investigators and PI
7.	Site Monitoring	Section 12.2, page 52	CDSA (Clinical Development Services Agency), an extramural centre of THSTI will be responsible for quality monitoring in the study.	Consensus of site investigators and PI

Letter of Amendment # 2

Date: June 29, 2016

Re: Letter of Amendment #2 for Protocol No. RCN/ZINCSEVINF/02/2015 Version 1.1 dated June 9, 2016

Title: Zinc as an adjunct for the treatment of clinical severe infection in infants younger than 2 months

To: Institutional Ethics Committees participating in and reviewing Protocol No. RCN/ZINCSEVINF/02/2015, Site Investigators

From: Dr. Nitya Wadhwa, Principal Investigator

The following information impacts the RCN/ZINCSEVINF/02/2015 study and must be forwarded to concerned Institutional Ethics Committee (IEC) as soon as possible for their information and review. This must be notified to the THSTI IEC and the respective hospital site IECs before implementation.

Upon notifying the THSTI IEC and the respective hospital site IECs of this LoA, The amended protocol will be considered the updated final protocol to be followed at the sites.

The following modifications are made to Protocol No. RCN/ZINCSEVINF/02/2015 Version 1.1 dated June 9, 2016.

S No	Protocol No. RCN/ZINCSEVINF/02/2015 version 1.1 dated June 9, 2016	Section/ page no in protocol no. RCN/ZINCSEVINF/02/2015 version 1.1 dated June 9, 2016	Proposed change in protocol no. RCN/ZINCSEVINF/02/2015 version 2.0 dated June 29, 2016	Reason for proposed change
1.	Age of inclusion 1d to 59 days.	i. Trial synopsis: primary and secondary objectives, page 11<br>ii. Study procedures on page 12	Age of inclusion modified to 3d to 59 days	Suggestion by external reviewer for protocol.<br>This has been done to increase the specificity of 'clinical severe infection'.<br>At 1d or 24 hrs of age there would be

		iii. Section 3, Study objectives, page 20<br>iv. Section 4, Trial design, page 23<br>v. Figure 1, page 24<br>vi. Section 5.5.2, eligibility criteria, page 26		an overlap of symptoms/ signs of ‘clinical severe infection’ and birth asphyxia or prematurity like chest indrawing (and fast breathing), poor feeding, lethargy. We would thus include many with no infection.<br>By modifying our inclusion age to say 3d (48hrs and more) to 59d we would exclude those neonates with birth asphyxia or prematurity where the signs of chest indrawing &/or poor feeding &/ or lethargy would have appeared within the first 48 hrs and would not appear for the first time on day 3.
2.	Eligibility for inclusion:	Section 5.5.2, page 28	To avoid including neonates or infants with birth asphyxia or prematurity who may have the signs of chest indrawing &/or poor feeding &/or lethargy, a criteria based on duration of the sign/ symptoms has been included.<br><br>**vi. Infant should have been well at some point in the first 48 hours of life.**	Suggestion by external reviewer for protocol.<br>This has been done to increase the specificity of ‘clinical severe infection’.
3.	Exclusion criteria: Weight at presentation < 1500 gm and weight-for-length ≤-3z at admission	Section 5.5.2.b, exclusion criteria for participants, page 29	Exclusion criteria: Replaced by weight-for-age at presentation cut off at <-4.5z.	Suggestion by external reviewer for protocol.<br>We now do not have one fixed cut off weight of < 1500 gm for a wide age

				range of 1d to 59 d of inclusion was not good and thus will not be excluding infants with weight at 1d of age of 1500 gm and thus weight-for-age <-4.5z and with the same weight at 59d of age, a weight-for-age <-7.5z.
4.	Standard Case Management: Antibiotic Protocol.	Section 5.6: Standard Case Management, Page 34	Each hospital site has a fixed antibiotic treatment algorithm and will follow the protocol strictly. Additionally, we will stratify the enrolled infant by hospital site and standardize the antibiotic treatment protocol across all hospital sites.<br>This has been added to the section to bring more clarity.	Suggestion by external reviewer for protocol.<br>The reviewer felt that having no fixed protocol at least hospital wise might be a confounder. It was clarified to him that each hospital has a fixed antibiotic treatment algorithm and will follow it strictly. Additionally we will be stratifying the infant by hospital site.<br>This has been clarified in the protocol by adding a statement.

Letter of Amendment # 3

Date: November 20, 2016

Re: Letter of Amendment #3 for Protocol No. RCN/ZINCSEVINF/02/2015 Version 2.0 dated June 29, 2016

Title: Zinc as an adjunct for the treatment of clinical severe infection in infants younger than 2 months

To: Institutional Ethics Committees participating in and reviewing Protocol No. RCN/ZINCSEVINF/02/2015, Site Investigators

From: Dr. Nitya Wadhwa, Principal Investigator

The following information impacts the RCN/ZINCSEVINF/02/2015 study and must be forwarded to concerned Institutional Ethics Committee (IEC) as soon as possible for their information and review. This must be notified to the THSTI IEC and the respective hospital site IECs before implementation.

Upon notifying the THSTI IEC and the respective hospital site IECs of this LoA, The amended protocol will be considered the updated final protocol to be followed at the sites.

The following modifications are made to Protocol No. RCN/ZINCSEVINF/02/2015 Version 2.0 dated June 29, 2016.

S No	Protocol No. RCN/ZINCSEVINF/02/2015 version 2.0 dated June 29, 2016	Section/ page no in protocol no. RCN/ZINCSEVINF/02/2015 version 3.0 dated Nov 20, 2016	Proposed change in protocol no. RCN/ZINCSEVINF/02/2015 version 3.0 dated Nov 20, 2016	Reason for proposed change
1.	Intervention formulation	i. Trial synopsis: study intervention and duration of study intervention, page 11<br>ii. Background Information, Name and Description of	Intervention formulation changed from drops to dispersible tablets	Inability of Dr Reddy's Laboratories to make placebo drops similar to the zinc drops.<br>We approached Nutriset, France to supply us the intervention- zinc and

		the Investigational Product, page 13<br>iii. Trial Design, page 23<br>iv. Randomization, Allocation Concealment, page 25-26<br>v. Randomization, Masking, page 26<br>vi. Interventions and Co-interventions, page 30-31<br>vii. Clinical Data Collection, page 33<br>viii. Supplies and Handling of Material, Intervention, page 39<br>ix. Ethical Issues and Approvals, page 46		placebo. Nutriset replied in the affirmative.<br>We have used dispersible zinc and placebo tablets supplied by Nutriset in our previous studies as well.<br>The intervention will be manufactured by a GMP certified company L.P. Rodael, France and procured from Nutriset, France. These tablets are covered by an international patent Rodael/Nutriset.
2.	Mode of giving intervention	i. Trial synopsis: duration of study intervention, page 11<br>ii. Randomization, Allocation Concealment, page 26<br>iii. Interventions and Co-interventions, page 30<br>iv. Clinical Data Collection, page 33	Dose of intervention 5 mg elemental zinc as dispersible tablets or placebo as dispersible tablets. The intervention will be dissolved in 2.5 ml expressed breast milk or distilled water twice a day 12 hours apart for a total of 14 days and given to the enrolled participants.	Intervention formulation changed from drops to dispersible tablets
2.	Supplier	i. Background Information, Name and Description of the Investigational Product, page 13<br>ii. Interventions and Co-	The intervention will be manufactured by a GMP certified company L.P. Rodael, France and procured from Nutriset,	Inability of Dr Reddy's Laboratories to make placebo drops similar to the zinc drops.<br>We approached Nutriset, France to supply us the intervention zinc and

		interventions, page 31<br>iii. Supplies and Handling of Material, Intervention, page 39	France. These tablets are covered by an international patent Rodael/Nutriset.	placebo. Nutriset agreed.<br>We have used dispersible zinc and placebo tablets supplied by Nutriset in our previous studies as well.
3.	Packaging of intervention: Allocation Concealment	i. Randomization, Allocation Concealment, page 25-26<br>ii. Interventions and Co-interventions, page 30-31<br>iii. Clinical Data Collection, page 33<br>iv. Supplies and Handling of Material, Intervention, page 39-40<br>v. Supplies and Handling of Material, Labeling, page 40<br>vi. Supplies and Handling of Material, Storage, page 40<br>vii. Supplies and Handling of Material, Clinical Supplies Accountability, page 40	Identically looking zinc sulphate and placebo dispersible tablets will be provided in identically looking blister packs. Each blister pack will contain 10 tablets of 5 mg each. Four such blister packs will be packaged in a zip-lock bag for each enrolled participant. The zip-lock bag/ intervention pack containing the 4 blister packs as well as each individual blister pack will be labeled with a 'unique serial number' which is the 'unique participant ID' and this will correspond to the randomization list	Intervention formulation changed from drops to dispersible tablets and for allocation concealment
4.	Residual syrup measure	Section 5.5.5, page 34<br>Section 10.2.2, page 51	Residual pill count	Intervention formulation changed from drops to dispersible tablets
5.	Modifications in labeling of cartons and blister packs of the intervention	Section 6, page 39-40	The labeling requirements for the cartons in which the intervention will be shipped to India and Nepal and that of the blister packs have been modified	These modifications have been made keeping in mind 'customs clearance' of the shipped cartons and the study and mandatory regulatory requirements for labeling of intervention packs. The modifications

				are after consensus of investigators and PI
**Some modifications bringing more detailing or clarity**				
6.	Initiation of life support at any time after enrolment until hospital discharge. Need for life support will be defined as a need for ventilation or vasoactive drugs at any time after enrolment until hospital discharge,	Section 3.2.2, page 20	Revised to "Initiation of life support at any time after enrolment until hospital discharge. Need for life support will be defined as a need for ventilation or vasoactive drugs at any time from the day of enrolment and till hospitalization for the illness episode "	To give more clarity to objective
7.	**To undertake mechanistic studies of immune system**	Section 3.2.2, page 21	**Revised to "To undertake mechanistic studies of immune system:** Change in Immunocytome of peripheral blood of infant from the time of enrolment to discharge."	To give more clarity to objective and after consensus of investigators and PI
8.	**Immunobiological readouts**	Section 3.3.2, page 22<br>Section 5.5.4, page 33	**Revised to "Immunobiological readouts:** Immunophenotyping of peripheral blood of infant at enrolment , 48-72 hrs of	To give more clarity to outcome and after consensus of investigators and PI

			study and discharge."	
9.	**Low body temperature (<35.5°C)**<br>Young infants can also respond to infection by dropping their body temperature to below 35.5ºC.<br>A digital thermometer that measures to a minimum of 35ºC will be used to measure temperature	Section 5.5.2, pages 27, 28	**Revised to "Low body temperature/ hypothermia (<35.5°C)**<br>Young infants can also respond to infection by dropping their body temperature to below 35.5ºC.<br>A digital thermometer that measures to a minimum of 35ºC will be used to measure axillary temperature"	To give more clarity and after consensus of investigators and PI
10.	To avoid including neonates or infants with birth asphyxia or prematurity who may have the signs of chest indrawing &/or poor feeding &/or lethargy, a criteria based on duration of the sign/ symptoms has been included.<br>**vi. Infant should have been well at some point in the first 48 hours of life.**	Section 5.5.2, page 29	To avoid including neonates or infants with birth asphyxia or prematurity who may have the signs of chest indrawing &/or poor feeding &/or lethargy, a criteria based on duration of the sign/ symptoms has been included.<br>**vii. Infant should have been well at some point from birth till the current episode of illness.**	To give more clarity and after consensus of investigators and PI

11.	Addition of paragraph	Randomization, Stratification, page 25	Addition of paragraph in the section "In addition, for the infants enrolled at VMMC & SJH, besides being stratified by hospital and presence of diarrhea, there will be additional strata of 'time of enrolment'. So separate randomization sequences will be generated for those enrolled between 5 am to 4 pm and those enrolled after 4 pm but before 5 am the next day. The infants enrolled at VMMC & SJH between 5 am and 4 pm will be eligible for the secondary objective of understanding the mechanism of how zinc is working in 'clinical severe infection' in infants. Peripheral blood collected from these infants at enrolment and discharge will be used for immunophenotyping. No additional blood will be collected for this purpose."	To explain explicitly how the objective of undertaking mechanistic studies of immune system will be limited to samples being collected from participants enrolled at one hospital.<br>Immunophenotyping and cell stimulation assays are very labour and cost intensive and therefore will be done in a limited number of participants.<br>With the proposed sample size we will have sufficient power to detect a difference in the two groups of zinc and placebo.

12.	Role and responsibility of "mother"	Section 5.5.2, page 27-28	Revised to role and responsibility of "mother/ caregiver"	
13.	The thermometer will be kept high in the axilla and the infant's arm will be held against her/ his body for full 3 minutes before reading the temperature.	Section 5.5.2a, page 28	Revised to "The thermometer will be kept high in the axilla and the infant's arm will be held against her/ his body till there is a beep, before reading the temperature."	Correction in the protocol for recording axillary temperature
14.	They will repeat the respiratory rate count if it is ≥ 60/min and the lowest of the two counts will be considered as the respiratory rate.	Section 5.5.5c, page 33	Revised to "They will repeat the respiratory rate count if it is ≥ 60/min and the second of the two counts will be considered as the respiratory rate."	Correction in the protocol for recording fast breathing
15.	**Laboratory procedures**	Section 5.7, page 35-38	Revisions made in<br>i. Immunobiological studies<br>ii. Volume of blood<br>iii. Tests to be performed with the separated sera<br>iv. Site for processing sample for blood culture	To give more clarity in the laboratory procedures
16.	Addition of section	Section 5.8, page 37<br>Section 14, page 56	Storage of leftover biospecimens	This section was mentioned in the informed consent document but had been inadvertently missed out in the protocol.
17.	Withdrawal Criteria	Section 7.0, page 41	Revised	Revised to give more clarity to withdrawal criteria
18.	Adverse Events	Section 8.1.1, page 41,42<br>Section 8.3, page 43	Revised	After consensus of site investigators and PI

19.	All adverse events will be graded for intensity and relationship to the intervention	Section 8.6, page 45	Revised to "All serious adverse events will be graded for intensity and relationship to the intervention"	After consensus of site investigators and PI
20.	(iv) in those with a positive septic screen (adapted from Center for Disease Control and Prevention criteria [42] and defined by a positive blood culture or presence of any two of the following parameters: total leucocyte count <5000/cmm; absolute neutrophil count <1500/cmm; band cell:neutrophil ratio >0.2; micro ESR >15mm at 1st hour and both of two C-reactive protein levels in specimens taken 24 h apart >1mg/dl).	Section 10.2.1e on subgroup analyses, page 49	Revised to "(iv) presence of sepsis, either 'culture positive' (true pathogen detected in blood culture) or 'clinical sepsis' defined by a negative blood culture but a positive septic screen with presence of any two of the following parameters: total leucocyte count <5000/cmm; absolute neutrophil count <1500/cmm; band cell:neutrophil ratio >0.2; micro ESR >15mm at 1st hour and C-reactive protein levels >1mg/dl (adapted from Center for Disease Control and Prevention criteria [42])."	To give more clarity in the way this subgroup analyses will be done
21.	Role of THSTI revised	Section 5.5.1, page 26<br>Section 18.1, page 65	Role of THSTI redefined and additional responsibility added "THSTI will also coordinate the immunological studies to explain potential mechanisms of clinical	After consensus of site investigators and PI

			severe infection and/or role of zinc in clinical severe infection."	
22.	Data capture in eCRF	Section 12.1, 12.2, page 53-54<br>Section 15, page 57-58	We have finalized the protocol for data collection by study staff.<br>All data screening and subsequent data capture except for SAE data will be in electronic CRFs.<br>The protocol has been modified to incorporate this	To reduce the time spent on query resolution and after consensus of other site investigators and PI
23.	Modifications in study organization and responsibilities of research staff	Section 16.2, pages 60-63	Modifications in study organization and responsibilities of research staff	To give more clarity and detailing and after consensus of investigators and PI
**Global changes in protocol**				
24.	Study duration	Section 3.2.2, page 20<br>Figure 1: Flow diagram of the trial, page 24	Change from 'after enrolment' to 'from the day of enrolment'	To be clear and consistent in our definitions
25.	Secondary objective no v	Trial synopsis, page 11<br>Section 3.2.2, page 21<br>Section 10.2.1c, page 48	Change from 'Time of death' to 'Time to death during hospitalization for the illness episode'	To be clear and consistent in our definitions
26.	Updation of terminology	-	The word 'caretaker' has been replaced with 'caregiver'	To be consistent
**Administrative modifications in protocol**				

27.	Investigators	Investigator names on page 3 and 4	Six investigators Drs Ajay Kumar, Rani Gera, Ratan Gupta, Sugandha Arya, Vijay Gupta, Karnika Saigal, B Talukdar have accepted to be site investigators. Their names and other details have been added to the list of investigators	After consensus of other site investigators and PI

Letter of Amendment # 4

Date: June 20, 2017

Re: Letter of Amendment #4 for Protocol No. RCN/ZINCSEVINF/02/2015 Version 3.0 dated Nov 20, 2016

Title: Zinc as an adjunct for the treatment of clinical severe infection in infants younger than 2 months

To: Institutional Ethics Committees participating in and reviewing Protocol No. RCN/ZINCSEVINF/02/2015, Site Investigators

From: Dr. Nitya Wadhwa, Principal Investigator

The following information impacts the RCN/ZINCSEVINF/02/2015 study and must be forwarded to concerned Institutional Ethics Committee (IEC) as soon as possible for their information and review. This must be notified to the THSTI IEC and the respective hospital site IECs before implementation.

Upon notifying the THSTI IEC and the respective hospital site IECs of this LoA, The amended protocol will be considered the updated final protocol to be followed at the sites.

The following modifications are made to Protocol No. RCN/ZINCSEVINF/02/2015 Version 3.0 dated Nov 20, 2016.

S No	Protocol No. RCN/ZINCSEVINF/02/2015 version 3.0 dated Nov 20, 2016	Section/ page no in protocol no. RCN/ZINCSEVINF/02/2015 version 3.1 dated June 20, 2017	Proposed change in protocol no. RCN/ZINCSEVINF/02/2015 version 3.1 dated June 20, 2017	Reason for proposed change
1.	Stabilization time/ Observation time	i. Figure 1: Flow Diagram of the, page 25<br>ii. Eligibility Criteria, page 31	Once we have determined eligibility in an infant and before randomization we see if the infant is in a stable condition to be administered	Inability to enroll some infants as they could not be stabilized within the initially proposed 8 hours to allow oral/ NG study medication.<br>We now propose to increase this

			the oral study medication. In case the infant is not found to be stable, the infant is put under an observation or stabilization phase. **The maximum duration allowed for an infant to stabilize and thus be enrolled, was 8 hours previously. We propose to extend this period to a maximum period of 24 hours.**	stabilization/ observation time to a maximum of 24 hours.<br>This gives ample time for stabilization and will not affect the safety of the infant.

Letter of Amendment # 5

Date: **March 07, 2018**

Re: Letter of Amendment #5 for Protocol No. RCN/ZINCSEVINF/02/2015 Version 3.1 dated June 20, 2017

Title: Zinc as an adjunct for the treatment of clinical severe infection in infants younger than 2 months

To: Institutional Ethics Committees participating in and reviewing Protocol No. RCN/ZINCSEVINF/02/2015, Site Investigators

From: Dr. Nitya Wadhwa, Principal Investigator

The following information impacts the RCN/ZINCSEVINF/02/2015 study and must be forwarded to concerned Institutional Ethics Committee (IEC) as soon as possible for their information and review. This must be notified to the THSTI IEC and the respective hospital site IECs before implementation.

Upon notifying the THSTI IEC and the respective hospital site IECs of this LoA. The amended protocol will be considered the updated final protocol to be followed at the sites.

The following modifications are made to Protocol No. RCN/ZINCSEVINF/02/2015 Version 3.1 dated June 20, 2017.

S No	Protocol No. RCN/ZINCSEVINF/02/2015 version 3.1 dated June 20, 2017	Section/ page no in protocol no. RCN/ZINCSEVINF/02/2015 version 4.0 dated March 07, 2018	Proposed change in protocol no. RCN/ZINCSEVINF/02/2015 version 4.0 dated March 07, 2018	Reason for proposed change
1.	Exclusion on weight criterion	i. Eligibility Criteria, 5.5.2b Exclusion criteria for participants, page 30	All infants with weight of <1500 gm at the time of enrolment will be excluded. Previously we were excluding all infants with weight-for-age z score of ≤ -4.5z	In an investigators meeting on study activities and assessment of preliminary data, there was a consensus decision that the trial is excluding a large number of low birth weight infants based on the weight-for-age criterion. This category of infants is not only at an increased risk of sepsis but sepsis in these infants carries a particularly high case fatality risk.<br>The investigators felt that by excluding this important patient category we may be depriving the most vulnerable babies of the benefits that zinc may give. We did not identify in the literature or in our implementation experience any safety concerns that would justify such exclusion, particularly if we continued to exclude babies with weight <1500gm.<br><br>Incidentally, in our initial submission to the Ethics Committee (Protocol Ver 1.0 dated 4-03-2016) we had weight of < 1500 gm as our criteria for excluding infants. This was subsequently modified in our protocol to the currently used exclusion criteria of weight-for-age z-scores ≤ -4.5, in response to one of the external reviewers who felt that the cut-off weight should vary according to age.<br><br>Based on our experience and the lack of safety concerns, we now propose to go back to our original weight based exclusion criteria of <1500 gm.

Letter of Amendment # 6

Date: **September 09, 2018**

Re: Letter of Amendment #6 for Protocol No. RCN/ZINCSEVINF/02/2015 Version 4.0 dated March 07, 2018

Title: Zinc as an adjunct for the treatment of clinical severe infection in infants younger than 2 months

To: Institutional Ethics Committees participating in and reviewing Protocol No. RCN/ZINCSEVINF/02/2015, Site Investigators

From: Dr. Nitya Wadhwa, Principal Investigator

The following information impacts the RCN/ZINCSEVINF/02/2015 study and must be forwarded to concerned Institutional Ethics Committee (IEC) as soon as possible for their information and review. This must be notified to the THSTI IEC and the respective hospital site IECs before implementation.

Upon notifying the THSTI IEC and the respective hospital site IECs of this LoA, The amended protocol will be considered the updated final protocol to be followed at the sites.

The following modifications are made to Protocol No. RCN/ZINCSEVINF/02/2015 Version 4.0 dated March 07, 2018.

S No	Proposed change in protocol No. RCN/ZINCSEVINF/02/2015 version 4.0 dated March 07, 2018	Proposed change	Section/ page no in protocol no. RCN/ZINCSEVINF/02/2015 version 4.1 dated Sep 09, 2018, where the changes have been proposed	Reason for proposed change
1.	Addition of Kalawati Saran Children's Hospital as study site in New Delhi, India	Kalawati Saran Children's Hospital has been added as a new site in New Delhi, India	i. List of study hospital sites in New Delhi, India, page 2<br>ii. Glossary, page 7<br>iii. Description of population; section 2.8, page 17<br>iv. Study setting; section 5.5.1, page 24<br>v. Contribution of each organization; section 18.1, page 64	Two of the hospital sites - Kanti Children's hospital, Nepal and Kasturba hospital, Delhi, India were closed in December 2018 and March 2019 following a consensus decision by all investigators based on the recommendation by the Data and Safety Monitoring Board for the study to review why the two sites were recruiting infants with milder sickness thus lowering event rates.<br>Following this the investigators agreed that an additional site should be initiated for recruitment of participants . Kalawati Saran Children's hospital (KSCH) in New Delhi was included as a site in India. The meeting of the KSCH IEC was held on 13th March 2019 and we received approval to conduct the study at the KSCH site on 13th April 2019. After the required site set up, recruitment and training of staff, the site was initiated on 8th July 2019.
**2.**	Blood sample collected for estimation of zinc, CRP and soluble	Word 'serum' replaced by 'plasma'	Laboratory procedure; section 5.7, pages 33, 34, 36	This was a typographical error. The blood sample for analysis of estimation of zinc, CRP and soluble markers of inflammation was always supposed to be processed for plasma

	markers of inflammation processed as 'plasma' instead of 'serum'			
3.	The list of Investigators has been moved from within the main content of protocol to Annexure I of the protocol. Subsequently, the numbering of the other annexures has been moved	• **Annexure I:** Addendum to Study Protocol with List of Investigators,<br>• **Annexure II**: Circular from DCGI for non-regulatory clinical trials & Gazette Notification from Ministry of Health & Family Welfare for Academic Clinical Trials<br>• **Annexure III**: DBT letter of approval to execute study under Programme of Cooperation<br>• **Annexure IV & V**: Site wise budget for study<br>• **Annexure VI:** Informed Consent	Page 44 and 65	For ease of access to the list of investigators
4.	Revised timelines of the study	Revised timelines of the study	Page 63	In view in closure of the Nepal sites and one site in India and start of an additional site in India, the timelines of the study were revised and updated timelines are on page 63 of the protocol.

Letter of Amendment # 7

Date: **January 18, 2021**

Re: Letter of Amendment # 8 for Protocol No. RCN/ZINCSEVINF/02/2015 Version 5.0 dated January 18, 2021

Title: Zinc as an adjunct for the treatment of clinical severe infection in infants younger than 2 months

To: Institutional Ethics Committees participating in and reviewing Protocol No. RCN/ZINCSEVINF/02/2015, Site Investigators

From: Dr. Nitya Wadhwa, Principal Investigator

The following information impacts the RCN/ZINCSEVINF/02/2015 study and must be forwarded to concerned Institutional Ethics Committee (IEC) as soon as possible for their information and review. This must be notified to the THSTI IEC and the respective hospital site IECs before implementation.

Upon notifying the THSTI IEC and the respective hospital site IECs of this LoA, The amended protocol will be considered the updated final protocol to be followed at the sites.

The following modifications are made to Protocol No. RCN/ZINCSEVINF/02/2015

**S. No**	**Application section (s)**	**Old text**	**New text**	**Reason for proposed change**
**1.**	**Protocol:** Proposed change in protocol No. RCN/ZINCSEVINF/02/2015 version 5.0 dated January 18, 2021			
	**i.** Addition of secondary objective:		x. Investigate whether the infants enrolled in the stratum with diarrhea more often have enteropathogens in their stools than those in the stratum without diarrhea, and characterize the intestinal microbiome/metagenome of the participating infants<br><br>**Section 1, pages 9**<br>**Section 3.2, page 19**	When we looked at yearly data on distribution of our enrolled infants with clinical severe infection that presented with associated diarrhea compared to those that presented without associated diarrhea, we observed a seasonal pattern. The proportion of infants presenting with associated diarrhea was higher in the summer and monsoon months. This seasonality mirrors the seasonality seen in the general population of children with acute diarrhea.<br>This observation led to the question: whether the infants enrolled in the stratum with diarrhea more often have enteropathogens in their stools than those in the stratum without and whether this reflects primary sepsis with GI manifestations or primary gastrointestinal infection with sepsis like symptoms?

				In our previous study (Bhatnagar et al, *Lancet,* 2012) conducted in 3 Delhi hospitals to measure efficacy of adjunct zinc administered to young infants with possible serious bacterial infection (PSBI), zinc had a 40% efficacy for reduction of treatment failure and most of the effect was actually seen among the babies who, in addition to PSBI, had diarrhea. The study did not encompass microbiological investigation of fecal specimens. To our knowledge, no study has adequately described the stool microbiology or the intestinal microbiome/metagenome of young infants with PSBI or sepsis.<br><br>In this study, for up to the last 500 infants that will be enrolled, we propose to describe the microbiology and characterize the intestinal microbiome/metagenome of the study participants, both to investigate whether the infants enrolled in the stratum with diarrhea more often have enteropathogens in their stools than those in the stratum without and to characterize the microbiome/metagenome of our infants with clinical severe infection'.<br>This will help us investigate whether diarrhea is caused by the known diarrheal enteropathogens or the diarrhoea in these cases is an associated symptom of sepsis and caused by pathogens other than enteropathogens
	**ii.** Addition of secondary outcome:		viii. Stool microbiology for enteropathogens and characterization of intestinal microbiome/metagenome.<br><br>**Section 3.3, pages 20**<br>**Section 5.5.4, page 31**	Same as above

	iii. The list of Investigators in Annexure I updated		Name of Dr Bhabatosh Das a from Translational Health Science and Technology Institute added	Dr Das will lead on the new secondary objective
	iv. Laboratory procedures		Addition of stool sample collection at baseline along with brief protocol for immediate processing and storage<br><br>**Section 5.7, page 33, 34 and 36**	Brief study protocol for collection, processing , shipment and storage added
	v. Analysis		Plan of analysis of stool sample added in section on Analyses of laboratory parameters<br><br>**Section 10.2, page 47**	
	vi. Revised timeline of study		Figure 2b added	To reconcile with study period extension till August 2021
**2.**	**Informed Consent Document**	Ver 3.0 dated 02-09-19	**Ver 4.0 dated 20-01-21 Following sentence added in section on study procedures:**<br><br>"Additionally, we will take a sample of your baby's	The sentences have been added to accommodate the addition of a secondary objective and collection of stool sample at baseline to fulfill the objective

			stool at admission to test for different germs that sometimes cause diarrhea and/ or severe infection. A diaper will be provided for easy collection of stool sample. Once your baby has passed stool, you will be requested to inform the study staff who will collect the fresh stool sample from the diaper. "<br><br>**Following sentence added in section on risks and discomfort:**<br><br>'At the time of admission, a sample of your baby's stool will be taken. This procedure will not cause any discomfort'	

Zinc as an adjunct for the treatment of clinical severe infection in infants younger than 2 months

Version 5.0
Dated: 18-01-2021
Protocol number: RCN/ZINCSEVINF/02/2015

Confidentiality Clause: The confidential information in this document is provided to you as an investigator for review by you, your staff and the applicable Institutional Review Board member. By accepting this document, you agree that the information contained herein will not be disclosed to others, without written authorization from Pediatric Biology Centre, Translational Health Science and Technology Institute, Faridabad

Funding:	Research Council of Norway (RCN) under the Research Grant on Global Health and Vaccination Research (GLOBVAC)<br>Research Council of Norway<br>P.O. Box 564<br>NO-1327 Lysaker, Norway<br><br>CISMAC (Centre for Intervention Sciences in Maternal & Child Health), Norway<br>Anchored at the Centre for International Health (CIH), University of Bergen, Norway

Partnering Institutes:	Pediatric Biology Centre, Translational Health Science and Technology Institute, Faridabad<br><br>Department of Child Health, Institute of Medicine, Tribhuvan University, Kathmandu, Nepal<br><br>Centre for International Health, Bergen, Norway<br>Innlandet Hospital Trust, Norway<br><br>Stanford University School of Medicine, USA<br><br>Radbound University, Nijmegen, The Netherlands

Coordinating Centre: Pediatric Biology Centre, Translational Health Science and Technology Institute, Faridabad

Study Hospital Sites

Delhi, India

- Maulana Azad Medical College (MAMC) and associated Lok Nayak Hospital
- Vardhman Mahavir Medical College & Safdarjung Hospital (VMMC & SJH)
- Chacha Nehru Bal Chikitsalaya (CNBC)
- Kasturba Hospital (KH) and
- Kalawati Saran Children's Hospital (KSCH)

Nepal

- Kanti Children's Hospital (KCH), Kathmandu and
- Institute of Medicine (IOM), Kathmandu.

Investigators:

List of Investigators in **Annexure I**

Table of Contents

GLOSSARY 6
1. TRIAL SYNOPSIS 9
2. BACKGROUND INFORMATION 11
2.1 Name and Description of the Investigational Product 11
2.2 Introduction 11
2.3 Relevant previous human studies 11
2.4 Rationale 12
2.5 Summary of known potential Risks and Benefits 12
2.6 Justification for Route of Administration, Dosage, Dose Regimen and Treatment Period 16
2.7 Rationale for Study Design 16
2.8 Description of Population 17
3. STUDY OBJECTIVES 17
3.1 Hypothesis 17
3.2 Objectives 18
3.3 Outcomes 19
4. TRIAL DESIGN 21
5. Randomization 23
5.1. Stratification: 23
5.2. Preparation and safe keeping of the randomization lists: 23
5.3. Allocation concealment: 23
5.4. Masking: 24
5.5. Study Procedures 24
5.6. Standard Case Management 32
5.7. Laboratory procedures 32
6. Supplies and Handling of Materials 37
6.2. Storage 38
6.3. Clinical Supplies Accountability 38
6.4. Source Documents 38
7. WITHDRAWAL CRITERIA 39
8. SAFETY MONITORING 39
8.1. Adverse Events 39
8.2. Relatedness to Intervention 41
8.3. Specification of Safety Parameters 41
8.4. Data Safety Monitoring Board 42

8.5. Independent study monitoring 42
8.6. Adverse event reporting 43
8.7. Follow Up After Adverse Events 43
8.8. Trial Termination 43
9. Ethical Issues and Approvals 44
9.1. Ethical issues: 44
9.2. Ethical Approvals 44
10. STATISTICS 44
10.1. Sample Size 44
10.2. Analysis 45
11. DIRECT ACCESS TO SOURCE DATA/ DOCUMENTS 49
12. QUALITY CONTROL AND QUALITY ASSURANCE 49
12.1. Staff Training 50
12.2. Site Monitoring 51
12.3. Auditing 52
13. ETHICS COMMITTEE CLEARANCES 53
14. CONSENT FOR PARTICIPATION 53
15. DATA HANDLING AND RECORD KEEPING 55
15.1. Data Management 55
15.2. Record Retention and Archival 56
16. STUDY ORGANIZATION 56
16.1. Governance 56
16.2. Study Organization 57
17. PROPOSED TIME LINE OF STUDY ACTIVITIES 62
18. CONTRIBUTION OF EACH ORGANIZATION 64
18.1. THSTI (PBC), SJH, MAMC, CNBC, KH 64
18.2. Tribhuvan University, Institute of Medicine, Nepal 64
18.3. Centre for International Health, University of Bergen, Norway (CHN-CIH) 64
18.4. Centres for Health Policy/Primary Care and Outcomes Research at Stanford University School of Medicine (CHP/PCOR) 64
18.5. Radbound University, Nijmegen, Netherlands 64
19. FINANCING AND BUDGET 64
20. PUBLICATION POLICY 65
21. REFERENCES 66

GLOSSARY

ANC: Absolute Neutrophil Count

CHP/PCOR: Centres for Health Policy/Primary Care and Outcomes Research

CIH: Centre for International Health

CISMAC: Centre for Intervention Sciences in Maternal & Child Health

CNBC: Chacha Nehru Bal Chikitsalaya

CRC: Clinical Research Coordinator

CRF: Case Report Form

DSMB: Data Safety Monitoring Board

EC: Ethics Committee

FT: Field Technician

GCP: Good Clinical Practice

GLOBVAC: Global Health and Vaccination Research

IEC: Institutional Ethics Committee

IOM: Institute of Medicine

IMCI: Integrated Management of Childhood Illnesses

IMNCI: Integrated Management of Childhood and Neonatal Illnesses

IRB: Institutional Review Board

KCH: Kanti Children’s Hospital

KH: Kasturba Hospital

KSCH: Kalawati Saran Children’s Hospital
MAMC: Maulana Azad Medical College
MO: Medical Officer
NHRC: Nepal Health Research Council
PAHS: Patan Academy of Health Sciences
PBC: Pediatric Biology Centre
PH: Patan Hospital
PI: Principal Investigator
PMT: Project Management Team
RCN: Research Council of Norway
REC: Regional Committees for Medical and Health Research Ethics
RO: Research Officer
SA: Study Assistant
SAE: Serious Adverse Event
SCU: Study Coordination Unit
SJH: Safdarjung Hospital
SN: Study nurse
SOP: Standard Operating Procedure
SRO: Senior Research Officer
THSTI: Translational Health Science and Technology Institute

TLC: Total Leukocyte Count

UiB: University of Bergen

VMMC: Vardhman Mahavir Medical College

WHO: World Health Organization

1. TRIAL SYNOPSIS

Study Title: Zinc as an adjunct for the treatment of clinical severe infection in infants younger than 2 months

Sponsor: Pediatric Biology Centre, Translational Health Science and Technology Institute, Department of Biotechnology, Ministry of Science and Technology, Govt of India

Funding Agency: Research Council of Norway (RCN) under the Research Grant on Global Health and Vaccination Research (GLOBVAC) and CISMAC (Centre for Intervention Sciences in Maternal & Child Health), Norway

Study Intervention: 10mg of elemental zinc per day given as oral zinc sulphate dispersible tablets Vs placebo dispersible tablets

Study design: Double-blind, randomized, placebo-controlled, parallel group, superiority trial.

Duration of study intervention: 5 mg elemental zinc given as dispersible tablets dissolved in expressed breast milk or distilled water twice a day 12 hours apart for a total of 14 days.

Objectives of study:

Primary objectives:

i. To estimate the efficacy of 10 mg elemental zinc administered orally as an adjunct to standard antibiotic therapy to infants aged 3 days (48 hours and more) up to 2 months (59 days) hospitalized with 'clinical severe infection' against **case fatality**.
ii. **Time to death until end of study period** defined as the time from enrolment to the time when an infant dies anytime until 12 weeks from the day of enrolment in the study.

Secondary objectives: Secondary objectives are to estimate the efficacy of 10 mg elemental zinc administered orally as an adjunct to standard antibiotic therapy to infants aged 3 days up to 2 months (59 days) hospitalized with 'clinical severe infection' against the following:

i. **Failure of treatment**
ii. **Time to cessation of symptoms and signs of clinical severe infection**
iii. **Time to failure of treatment**
iv. **Time to discharge**
v. **Time to death** during hospitalization for the illness episode
vi. **Death at any time after discharge from hospital until end of study period which is 12 weeks from the day of enrolment.**

vii. **Severe illness requiring hospitalisation at any time after discharge from hospital until end of study period which is 12 weeks from the day of enrolment.**

viii. **Cost-effectiveness analysis**

*(Refer to **Annexure VII** :addendum protocol on extended cost effectiveness analysis [ECEA] for the expanded scope of a secondary objective on Cost-effectiveness analysis)*

ix. **To undertake mechanistic studies of immune system**

Study Procedures: The trial will be conducted over a period of 3 years and 6 months. This is a multicenter study where recruitments will take place in 7 centres, 5 in New Delhi India and 2 in Kathmandu, Nepal. The trial will measure the efficacy of zinc administered orally as an adjunct to standard therapy to infants aged 3 days to 2 months hospitalized with clinical severe infection identified using an adaptation of the WHO Integrated Management of Childhood Illnesses (IMCI) criteria. The participants will be randomized to receive zinc or placebo in a 1:1 allocation ratio. The intervention (zinc/ placebo dispersible tablets) will be co-administered with the standard therapy which includes intravenous antibiotics and other supportive therapy like intravenous fluids, supplemental oxygen, etc. daily at 12 hrly intervals from the time of enrolment for 14 days. 4140 infants with clinical severe infection will be enrolled, given intervention for 14 days and followed up till discharge and until 12 weeks from the day of enrolment.

Biospecimen collection:
Blood sampling for the study:

i. At enrolment: 3 ml of venous blood will be collected at admission (enrolment) from the enrolled infants with clinical severe infection for study related investigations
ii. At 48-72 hours: 3 ml of blood will be collected
iii. At discharge: 3 ml of blood will be collected

2. BACKGROUND INFORMATION

2.1 Name and Description of the Investigational Product

Elemental zinc given as zinc sulphate dispersible tablets (manufactured by a GMP certified company L.P. Rodael, France and procured from Nutriset, France). These tablets are covered by an international patent Rodael/Nutriset.

2.2 Introduction

More than 3 of the 7.6 million global deaths in children under 5 years of age occur in the neonatal period[1] and about 75% in the first week of life.[2] More than 70% of the neonatal deaths occur in Africa and South East Asia.[1] Severe infections like pneumonia and sepsis contribute to 25% of these deaths [3] and are also a major cause of hospitalization in infants.[4] Despite appropriate antimicrobial therapy, the outcome of these severe infections in early infancy is poor.[5] Effective interventions that can be added to standard therapy for severe infections are required to improve clinical outcomes and to reduce case fatality.

Serious infections like pneumonia, sepsis, and meningitis contribute to more than 25% of the annual 1 million neonatal deaths in India.[6] Also in Nepal, sepsis is a leading cause of death in neonates, and the second most frequent reason for hospitalization.[7] While appropriate antibiotics are available in many hospitals in India and Nepal, second-line antibiotics may be unavailable or are prohibitively expensive in peripheral health facilities. It is important to develop inexpensive, effective and accessible interventions that can be added to standard therapy for severe infections to improve treatment outcomes and reduce case fatality.

2.3 Relevant previous human studies

In a recent randomized placebo-controlled trial conducted in 3 tertiary hospitals in New Delhi, it was found that 10 mg of elemental zinc given daily to 7 to 120 days old infants treated with antibiotics for probable serious bacterial infection (PSBI) carried a 40% (95% CI 10% to 60%) efficacy against treatment failure.[8] The absolute risk reduction was 6.8% (95% CI 1.5% to 12.0%), indicating that 15 (95% CI 8 to 67) infants would need to be treated with zinc in addition to antibiotics to prevent one treatment failure. This study of 700 infants is, to our knowledge, the first report of the efficacy of zinc in the treatment of PSBI in infants younger than 4 months. An even larger efficacy against treatment failure (54% [95% CI 20% to 74%; P=0.005)] was seen when analysis was restricted to 1 week to 2 month old infants, among whom only 11 (95% CI 6 to 37) would need to receive adjunct zinc treatment to prevent one treatment failure. The point estimate for the efficacy of adjunct zinc therapy against death (43%) was the same as that against treatment failure, albeit with poorer precision (95%CI -23% to 73). However, the study was not powered to estimate the effect of zinc on case fatality.

Our main hypothesis that zinc will reduce the risk of treatment failure in infants with clinical severe infection was based on publications showing that zinc is crucial for immune function [9-11] and the benefit of oral zinc in infectious diseases like acute childhood diarrhoea.[12-15]

2.4 Rationale

More than 3 of the 7.6 million global deaths in children under 5 years of age occur in the neonatal period[1] and about 75% in the first week of life.[2] More than 70% of the neonatal deaths occur in Africa and South East Asia.[1] Severe infections like pneumonia and sepsis contribute to 25% of these deaths [3] and are also a major cause of hospitalization in infants.[4] Despite appropriate antimicrobial therapy, the outcome of these severe infections in early infancy is poor.[5] Effective interventions that can be added to standard therapy for severe infections are required to improve clinical outcomes and to reduce case fatality.

Based on the promising results of the above-mentioned trial, [8] a large, multicentre study powered to examine the effect of zinc on case fatality from clinical severe infection would contribute evidence towards revising treatment recommendations for low resource settings in South Asia and elsewhere.

2.5 Summary of known potential Risks and Benefits

Risks

Zinc is a micronutrient that can be found in all tissues of the body and is essential for cell growth, cell differentiation and DNA synthesis.[16] It is also essential for the maintenance of a healthy immune system.[17] Human zinc deficiency was first identified by Prasad et al. in the 1960s in growth-retarded adolescent boys in Egypt .[18] A population level analyses from food balance sheets have estimated that 21% of the world's population is at risk of zinc deficiency.[19] Children in many developing countries around the world typically consume fewer animal products than adults, especially in the developing world, which results in both stunting and zinc deficiency. A high rate of stunting is considered indicative of zinc deficiency among children less than 5 years of age.[19] WHO has identified zinc deficiency as a major risk to child health, and has linked it to morbidity from diarrhoea, lower respiratory tract infections and malaria, accounting for 0.8 million-child deaths per year.[20]

The International Zinc Consultative Group (IZiNCG) revised the recommended dietary allowances (RDAs) in 2004.[21] These recommendations suggest the following:

Group	RDA of zinc
Infants	4–5 mg
Children 1–3 years of age	3 mg
Children 4–8 years of age	4–5 mg
Non-pregnant women	8–9 mg
Pregnant and lactating women	9–13 mg
Men	13–19 mg

These recommendations take into account differences in diet and are based on a standard reference body weight. Children receiving diets higher in phytate, which is

found in unrefined cereals, will need to consume more zinc each day to achieve the physiological requirement. In addition, these guidelines are for healthy children and do not take into account the excess zinc losses during an episode of diarrhoea[22, 23] or the extra zinc required for catch-up growth and development.

In extensive safety studies undertaken in laboratory animals, zinc has been shown *not* to be carcinogenic, mutagenic or teratogenic .[24] In addition, the human body has efficient homeostatic mechanisms that regulate the absorption and retention of zinc, and these reduce the likelihood of toxic build-up in the body .[25] Zinc toxicity in adults can occur following moderately high intakes of zinc (>150 mg/day or approximately 10 times the RDA) over a long period of time or from ingestion of >1 g of zinc (more than 60 times the RDA) by overdose via supplementation or intravenous feeding .[26] Ingesting too much zinc at once can cause gastric distress and the typical signs and symptoms often associated with food poisoning.

High doses of zinc for long periods may lead to a lower concentration of plasma lipoproteins and decreased copper absorption.[26] A lower copper status may also inhibit the transport of iron and result in anaemia.[27] Although zinc-induced copper deficiency and the resulting anaemia are serious, they occur only after excessive zinc intake over a long period and are easily corrected by adjusting the intake of zinc and copper accordingly.[25] Supplements of zinc and iron may also compete for absorption in the body.

Cases have been reported of acute, though reversible, adverse reactions from inhaling zinc vapours – a condition known as “zinc fume fever” – and from ingesting food or drink stored in galvanized containers.[25] There are also a number of reported cases of adverse effects due to excessive zinc intake. The majority of these cases involved adults who knowingly ingested many times the normal daily dose of zinc over a long period. Even in the most extreme cases (more than 1 g/day for many months), patients recovered from all signs and symptoms, including fatigue, gastrointestinal discomfort and anaemia, as soon as zinc intake was decreased and serum zinc returned to the normal range.

In the report of the WHO Collaborating Centre for International Drug Monitoring in Uppsala, Sweden, there were 50 cases of adverse effects of oral zinc sulphate, including 56 clinical signs and symptoms. The majority of these cases involved the patient taking or being given several drugs at the same time, so it was not always possible to identify the cause of the observed sign(s) and/or symptom(s). The cases documented in the report all varied with respect to dosage, patient age, certainty of causality of association and number of additional drugs. **There were only 2 reported cases in which the likelihood of causation by zinc was “certain”, and only 1 in a child (side-effect: epistaxis) There were 20 reported cases where the likelihood was “possible” and 9 where it was “probable.”** The level of causation could not be established in the other reported cases. There were four reports of possible adverse responses to zinc ingestion among children less than 10 years of age.

Short-course supplementation trials

After more than 20 years of extensive research, zinc supplementation for the treatment of diarrhoea is now recommended by WHO and UNICEF .[28] Current recommendations are for 10–14 days of supplementation for all episodes of diarrhoea among children less than 5 years of age. Infants less than 6 months of age are to receive 10 mg daily and

children aged between 6 months and 5 years are to receive 20 mg daily. These doses have been proven to be both effective and safe for treatment during diarrhoea.

To date there have been no reports of severe adverse reactions from any form of zinc treatment for diarrhoea. Trials have included more than 9100 children who have participated in efficacy trials in both the placebo and zinc study arms, and nearly 12 000 child-years of observation from one large effectiveness trial. **The zinc doses ranged from 5 to 45 mg/day and were well tolerated in diverse settings. No differences in adverse reactions were found based on the different zinc salts used in supplementation trials, i.e. the sulphate, acetate and gluconate.**

At present, the only reported side effect of zinc treatment has been vomiting. Of the seven trials that have reported incidences of vomiting, only two reported more vomiting in the zinc-treated children compared with those given a placebo.[29, 30] One trial reported more vomiting than in control children when zinc was given with multiple micronutrients but not when given alone.[31]

Copper status has been evaluated in four trials. In three of these, no difference in serum copper status was found after supplementation.[29, 32,33] In the fourth trial, a significant trend towards lower copper status was found in zinc-treated compared to untreated children[34]; however, these children were malnourished with persistent diarrhoea at baseline. Overall, there is no substantial evidence that short-term zinc administration for the treatment of diarrhoea adversely affects copper status.

In addition to trials on the treatment of diarrhoea, there have been several trials assessing the efficacy of zinc for the treatment of pneumonia, malaria, measles and the common cold. Treatments have typically included approximately 20 mg/day for the duration of the illness, which is typically less than two weeks. No serious adverse effects linked to zinc have been reported in these studies.

In a **recent clinical trial in India** where 700 infants aged 7d -120d were given either 10 mg elemental zinc or placebo, no serious adverse events were demonstrated to be associated with oral zinc supplementation when given in a dose of 10mg elemental zinc per day till recovery and hospital discharge. The risk of adverse events that were not components of the clinical outcomes was similar in the two trial arms

**Adverse events***	**Zinc n=352**	**Placebo n=348**	**RR (95% CI)**
Vomiting related to the time of intervention:	3 (0·85)	3 (0·86)	0·99 (0·20, 4·86)
Vomited the first dose of intervention within 30 minutes	6 (1·70)	6 (1·72)	0·99 (0·32, 3·04)
Vomited any subsequent intervention within 30 minutes			
Vomited once or more times during the study period unrelated to time of intervention	41 (11·65)	36 (10·34)	1·13 (0·74, 1·72)
Abdominal distension lasting $\geq$ 24 hours at any time during the study period	13 (3·69)	10 (2·87)	1·28 (0·57, 2·89)

Long-term supplementation trials

A number of long-term supplementation trials have been carried out among young children and pregnant women. Children have been given zinc supplements to improve growth and to prevent pneumonia, diarrhoea and malaria. In 1999, a review of seven zinc supplementation studies for the prevention of diarrhoea and pneumonia was published .[15] These trials were conducted in a variety of study populations with different baseline nutritional status. Supplementation with zinc ranged from 5 to 20 mg/day for up to a year. No adverse effects were reported in these studies. There have been two studies in which children were given zinc supplements for the prevention of malaria. Children were given supplements of up to 70 mg zinc, twice a week for up to 15 months, and no adverse effects were reported.

One supplementation trial of low-birth-weight infants demonstrated not only the safety of zinc but the benefits of daily supplementation among these vulnerable babies .[35] A total of 581 Indian infants aged 30–284 days were each given 5 mg zinc. Those who received the zinc supplementation had a two-thirds lower risk of dying over the study period. There were 5 deaths among infants receiving zinc and 15 deaths among infants who did not receive zinc. There were no adverse events linked to zinc supplementation in this trial.

There have been several trials of zinc supplementation among pregnant women .[36] Because the demands of zinc increase during pregnancy, zinc supplementation may provide benefits to the pregnant mother and the growing fetus, especially in countries where zinc intake is lower than the recommended standards. Although the benefits of providing pregnant women with zinc supplements have yet to be confirmed, there are no published reports of adverse effects during these trials in either the pregnant women or their infants.

Further studies on **zinc supplementation** have been completed in the past two years, involving thousands of children who received either **10 mg elemental zinc per day for up to two years**[37] or 40 mg zinc for up to 14 days for the management of acute diarrhoea .[38] Except for mild regurgitation in a few studies, no serious adverse effects of oral zinc were seen in any of the studies.

Precautions

As with any treatment, zinc supplements should be kept in a safe place to prevent the accidental ingestion of more than the recommended dose. In the unlikely event that a child consumes several daily doses of the supplements, he/she would probably vomit quickly. There is no evidence to suggest that further adverse events would occur but, as in the case of any accidental ingestion of medication, the child should be taken to a health care provider.

Conclusion

Zinc supplementation is a safe and effective treatment for diarrhoea. Zinc has also been shown to be safe in long-term supplementation studies. The most severe adverse effects noted in supplementation trials have been vomiting in some cases and a slight reduction in copper status in some children. Neither has been shown to cause any long-term harm. Although there have been case reports in adults of excessive zinc intake,

the adverse effects even in these cases have been limited to short-term morbidity, and few have resulted in any long-term sequelae.

Potential benefits to enrolled participants in the current clinical trial could be reduced risk of case fatality, need for changing antibiotics, prolonged hospital stay and use of high-generation antimicrobials; education on personal hygiene and access to medical care for the participant for any illness occurring during the study period.

2.6 Justification for Route of Administration, Dosage, Dose Regimen and Treatment Period

2.6.1 Route of Administration: The ease of administration by oral route makes this route a preferred choice. If zinc is proven to be effective in reducing case fatality in infants with clinical severe infection, care providers at small health-care facilities can be trained to initiate treatment of clinical severe infection with antibiotics (as recommended by IMNCI) and oral zinc before transferring infants to appropriately equipped facilities.

2.6.2 Dosage: In a recent randomized placebo-controlled trial conducted in 3 tertiary hospitals in New Delhi, it was found that 10 mg of elemental zinc given daily to 7 to 120 days old infants treated with antibiotics for probable serious bacterial infection (PSBI) carried a 40% (95% CI 10% to 60%) efficacy against treatment failure. In this study about 43%-enrolled infants were found to have low serum zinc at enrolment. After supplementation there was a 39% increase in serum zinc levels at discharge suggesting that this dose is adequate.

In addition to being efficacious, no serious adverse events were demonstrated to be associated with oral zinc supplementation when given in a dose of 10mg elemental zinc per day till recovery and hospital discharge.

2.6.3 Dose Regimen: In the above-mentioned placebo-controlled trial conducted in New Delhi, oral zinc was given in the dose of 10mg elemental zinc divided in two doses given 12 hours apart.

We will replicate the same dose regimen of 5mg of elemental zinc given 12 hourly in our current study

2.6.4 Treatment Period: Once enrolled in the study oral zinc will be given in the dose of 10mg elemental zinc for a total of 14 days. This is based on previous studies where beneficial effects of zinc beyond the supplementation period have been seen when it was given for 14 days.

2.7 Rationale for Study Design

The study has been designed as a placebo-controlled randomized trial similar to the design of our previous study on the efficacy of zinc used as an adjunct to standard antibiotic therapy in reducing treatment failure in young infants with probable serious bacterial infection. The study has similar safeguards for standard medical care in place to minimize risk of adverse events for the participants.

If the results of this study are consistent with our earlier trial, this would substantially strengthen the evidence for recommending zinc as an adjunct to standard therapy for

clinical severe infection in young infants. Further, as we propose to identify young infants with clinical severe infection using an adaptation of IMNCI criteria, it would be easier to justify introduction of zinc in the national programs where IMNCI is followed.

2.8 Description of Population

The patient recruitment will be done in 7 centres: 5 secondary level hospitals in Delhi, India (Maulana Azad Medical College (MAMC) and associated Lok Nayak Hospital, Vardhman Mahavir Medical College & Safdarjung Hospital (VMMC & SJH), Chacha Nehru Bal Chikitsalaya (CNBC), Kasturba Hospital (KH) and Kalawati Saran Children's Hospital (KSCH)), and 2 centres in Nepal (Kanti Children's Hospital (KCH), Kathmandu and Institute of Medicine (IOM), Kathmandu), The study will be coordinated by PBC at THSTI.

India centres: The selected hospitals in North India are secondary referral level hospitals that are responsible for a defined geographical area. Their pediatric department bed strength is around 150-250 beds depending on the size, terrain and population of the area covered by the hospital. Secondary level hospitals provide all basic specialty services. Almost 25% of pediatric admissions at these hospitals are for infant sepsis.

Nepal centres: KCH has a capacity of 300 beds. About 50% of the referrals are from outside the Kathmandu valley. Neonatal sepsis accounts for 50% of the total admissions in the neonatal intermediate care unit of this hospital. IOM, with a 60-bedded pediatric ward, recently opened a neonatal nursery and separate neonatal and pediatric intensive care units and admits some 300 patients each month, of whom almost 50 have sepsis.

The selected clinical sites are secondary level hospitals with similar standard of care. This will make it easier to standardize study protocol across the hospitals. Each of the seven hospitals has a large patient load particularly of young infants with clinical severe infection.

The above hospitals have been selected because they get a large number of young infants with clinical severe infection and are therefore most relevant for this intervention.

3. STUDY OBJECTIVES

3.1 Hypothesis

Our hypothesis is that daily administration of 10 mg elemental zinc orally as adjunct to standard therapy to infants aged 3 days up to 2 months (59 days) hospitalized with clinical severe infection will lead to a relative case fatality risk reduction of at least 30%.

3.2 Objectives

3.2.1 Primary objectives:

i. To estimate the efficacy of 10 mg elemental zinc administered orally as an adjunct to standard antibiotic therapy to infants aged 3 days (48 hours and more) up to 2 months (59 days) hospitalized with ‘clinical severe infection’ against **case fatality**.
   The case fatality risk is the proportion of children with ‘clinical severe infection’ who die due to any cause and at any time from the day of enrolment and till hospitalization for the illness episode.

ii. **Time to death until end of study period** defined as the time from enrolment to the time when an infant dies anytime until 12 weeks from the day of enrolment in the study.

3.2.2 Secondary objectives:

Secondary objectives are to estimate the efficacy of 10 mg elemental zinc administered orally as an adjunct to standard antibiotic therapy to infants aged 3 days up to 2 months (59 days) hospitalized with ‘clinical severe infection’ against the following:

i. **Failure of treatment**. There is failure of treatment if one or more of the following events occur:

   a) Death at any time during hospitalization for the illness episode starting from the day of enrolment, i.e. as primary objective i.,

   or

   b) Initiation of life support at any time after enrolment until hospital discharge. Need for life support will be defined as a need for ventilation or vasoactive drugs at any time from the day of enrolment and till hospitalization for the illness episode,

   and/or

   c) A change in antibiotics for one of the following circumstances:

      i) Persistence of signs that indicate ‘clinical severe infection’ present at the time of enrolment anytime after 48 hours of enrolment and prior to discharge.

      ii) Worsening of existing signs or appearance of new signs of ‘clinical severe infection’ any time after enrolment and prior to discharge

      iii) Reappearance of signs of ‘clinical severe infection’ that the infant presented with at time of enrolment anytime after 48 hours of disappearance and prior to discharge.

ii. **Time to cessation of symptoms and signs of clinical severe infection**: This will be defined as the time from enrolment to the beginning of a 48 hour period with none of the signs of ‘clinical severe infection’.

iii. **Time to failure of treatment**: This will be defined as the time from enrolment to the time when an infant is deemed to have failed treatment as per the definition of **secondary objective i**.

iv. **Time to discharge**: The time from enrolment to discharge from the hospital.

v. **Time to death during hospitalization for the illness episode**: This will be defined as the time from enrolment to the time when an infant dies while admitted in the hospital for 'clinical severe infection'.

vi. **Death at any time after discharge from hospital until end of study period which is 12 weeks from the day of enrolment.**

vii. **Severe illness requiring hospitalisation at any time after discharge from hospital until end of study period which is 12 weeks from the day of enrolment.**

viii. **Cost-effectiveness analysis:** To measure the incremental cost effectiveness ratio of zinc supplementation as an intervention for 'clinical severe infection' in infants < 2 months.

*(Refer to **Annexure VII** :addendum protocol on extended cost effectiveness analysis [ECEA] for the expanded scope of a secondary objective on Cost-effectiveness analysis)*

ix. **To undertake mechanistic studies of immune system:** Change in Immunocytome of peripheral blood of infant from the time of enrolment to discharge.

3.3 Outcomes

3.3.1 Primary Outcomes

i. Case fatality, which is death due to any cause and at any time from enrolment until infant is in hospital for this episode of illness.

ii. **Time to death until end of study period** defined as the time from enrolment to the time when an infant dies until 12 weeks from the day of enrolment in the study.

3.3.2 Secondary Outcomes

i. **Failure of treatment**. There is failure of treatment if one or more of the following events occur:

a) Death at any time during hospitalization for the illness episode starting from the day of enrolment, i.e. as primary outcome i.,

or

b) Initiation of life support at any time after enrolment until hospital discharge. Need for life support will be defined as a need for ventilation or vasoactive drugs at any time after enrolment until hospital discharge,

and/or

c) A change in antibiotics for one of the following circumstances:

i) Persistence of signs that indicate 'clinical severe infection' present at the time of enrolment anytime after 48 hours of enrolment and prior to discharge.

ii) Worsening of existing signs or appearance of new signs of 'clinical severe infection' any time after enrolment and prior to discharge.

iii) Reappearance of signs of 'clinical severe infection' that the infant initially presented with at time of enrolment anytime after 48 hours of disappearance and prior to discharge

ii. **Cessation of signs of clinical severe infection**: This will be defined as the beginning of a 48 hour period with none of the signs of 'clinical severe infection'.

iii. **Discharge**: Defined as discharge from the hospital.

iv. **Death at any time after discharge from hospital until end of study period which is 12 weeks from the day of enrolment.**

v. **Severe illness requiring hospitalisation at any time after discharge from hospital until end of study period which is 12 weeks from the day of enrolment.**

vi. **Cost-effectiveness analysis:** incremental cost effectiveness ratio of zinc supplementation as an intervention for 'clinical severe infection' in infants < 2 months.

*(Refer to **Annexure VII** :addendum protocol on extended cost effectiveness analysis [ECEA] for the expanded scope of a secondary objective on Cost-effectiveness analysis)*

vii. **Immunobiological readouts:** Immunophenotyping of periphral blood of infant at enrolment , 48-72 hrs of study and discharge.

4. TRIAL DESIGN

The trial will be conducted in compliance with the protocol and ICH (http://www.ich.org/home.html) guidelines.

The trial is a double-blind, randomized, placebo-controlled, parallel group, superiority trial. The trial will measure the efficacy of zinc administered orally as an adjunct to standard therapy to infants aged 3 days to 2 months hospitalized with clinical severe infection identified using an adaptation of the WHO Integrated Management of Childhood Illnesses (IMCI) criteria on reducing case fatality. The trial will also test the efficacy of zinc administered orally as an adjunct to standard therapy to infants aged 3 days to 2 months hospitalized with clinical severe infection on increasing time to death until end of the study period which is 12 weeks from the day of enrolment as another primary outcome.

This trial will also assess (i) failure of treatment, defined as a need to change antibiotics or requirement for life support or death, (ii) time to cessation of clinical symptoms and signs of clinical severe infection, (iii) time to failure of treatment and (iv) time to discharge (v) time to death (vi) Death at any time after discharge from hospital until end of study period which is 12 weeks from the day of enrolment (vii) Severe illness requiring hospitalization at any time after discharge from hospital until end of study period which is 12 weeks from the day of enrolment. An evaluation of the incremental cost effectiveness of zinc supplementation will be conducted to inform policy. This trial will also include mechanistic studies of the immune system during the episode of 'clinical severe infection'.

The participants will be randomized to receive zinc or placebo in a 1:1 allocation ratio. The intervention (zinc/ placebo dispersible tablets) will be co-administered with the standard therapy which includes intravenous antibiotics and other supportive therapy like intravenous fluids, supplemental oxygen, etc. daily at 12 hrly intervals from the time of enrolment for 14 days. **4140 infants with clinical severe infection will be enrolled, given intervention for 14 days and followed up till discharge and until 12 weeks from the day of enrolment.**

Figure 1: Flow diagram of the trial

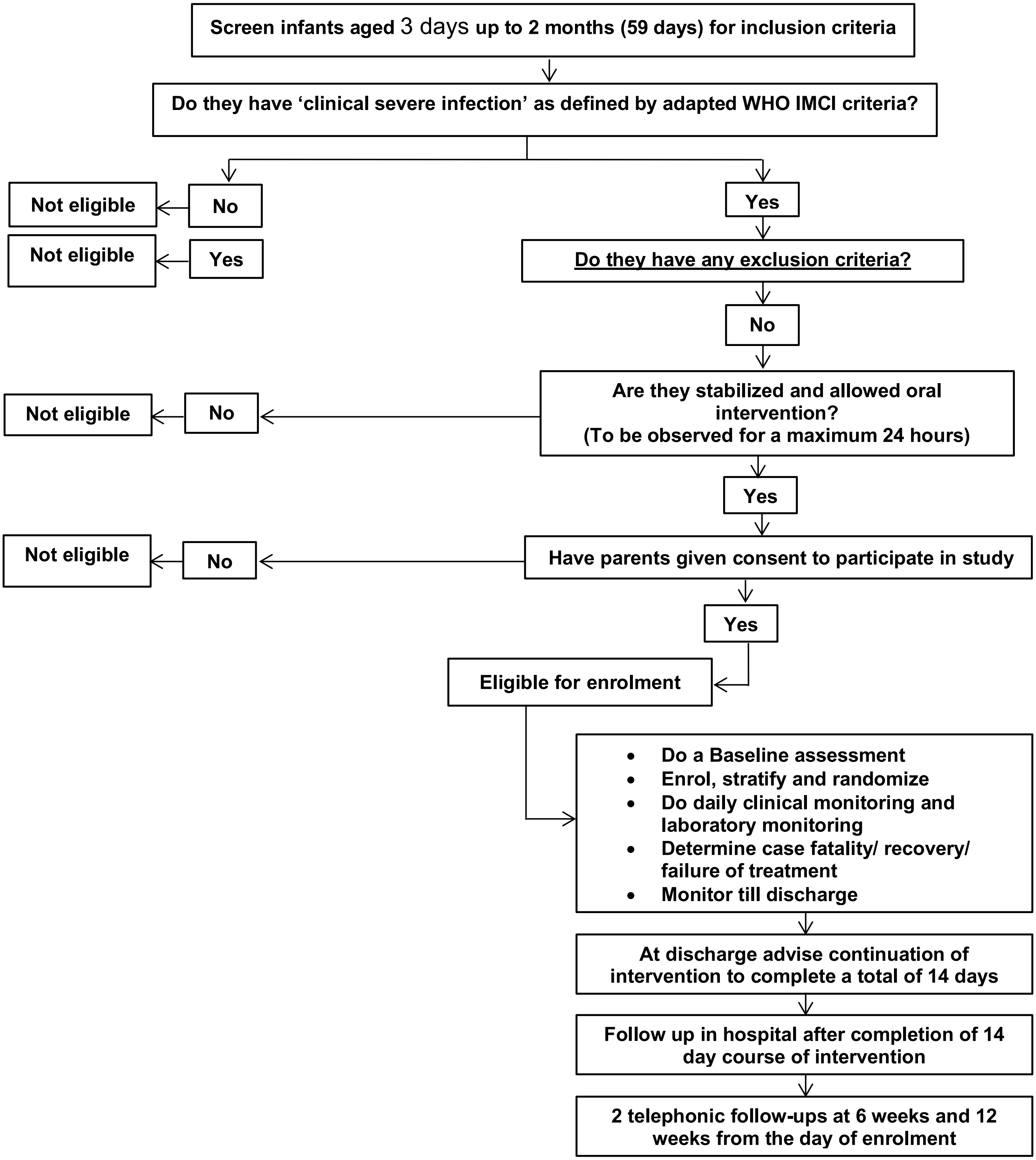

5. Randomization

5.1. Stratification:

The infants fulfilling eligibility criteria will be **stratified by hospital and by the presence or absence of diarrhea**.

Diarrhea will be defined as passage of 3 or more liquid or watery stools per day. The normally frequent or loose stool of a breastfed baby is not diarrhea. The mother of a breastfed baby can recognize diarrhea because the consistency or frequency of the stools is different from normal.

In addition, for the infants enrolled at VMMC & SJH, besides being stratified by hospital and presence of diarrhea, there will be additional strata of ‘time of enrolment’. So separate randomization sequences will be generated for those enrolled between 5 am to 4 pm and those enrolled after 4 pm but before 5 am the next day. The infants enrolled at VMMC & SJH between 5 am and 4 pm will be eligible for the secondary objective of understanding the mechanism of how zinc is working in ‘clinical severe infection’ in infants. Peripheral blood collected from these infants at enrolment and discharge will be processed for immunophenotyping using fresh blood. No additional blood will be collected for this purpose.

5.2. Preparation and safe keeping of the randomization lists:

A statistician/ scientist not otherwise involved in the trial will generate the allocation sequences using STATA (StataCorp, College Station, Texas, USA) or equivalent software such that, within each hospital and stratum, patients are randomized in the ratio of 1:1 in permuted blocks of 8 to receive zinc or placebo. The randomization list will be generated for 4200 infants. A copy of the generated sequence will be kept with this statistician.

Duplicate copies of the randomization sequence will be kept safely with 2 more scientists not involved in the study and will not be available to any of the investigators until all data have been collected and cleaned, and the database is locked.

Procedures for breaking the randomization codes:

- The randomization codes and allocation sequence will be generated by an independent statistician/ scientist not otherwise related to the study.
- The DSMB will be authorized to unblind the treatment allocation if required during the trial.
- The DSMB may unblind under the following conditions:
  - An unanticipated/Unsolicited serious adverse event (SAE) that has been judged to be related to the intervention by either the principal investigator/ designee.
  - A SAE that has been judged to be related to the intervention and is of a severity or frequency higher than anticipated.
  - An anticipated or other SAE where the management of the adverse event may depend on the intervention received.
  - Other conditions where the DSMB may feel the need to unblind.

5.3. Allocation concealment:

Identically looking zinc sulphate and placebo dispersible tablets will be provided in identically looking blister packs. Each blister pack will contain 10 tablets of 5 mg each. Four such blister packs will be packaged in a zip-lock bag for each enrolled participant.

The blister packs and the zip-lock bag will be labeled with a unique serial number according to the randomization list.

The study nurse/ medical officer will, after obtaining an initial verbal consent for the screening, screen all infants coming to the emergency rooms of the hospitals. Once eligibility has been determined and informed consent for participation in the trial obtained, a unique ‘participant ID’ will be assigned to the participant. The study nurse/ medical officer will be provided with the intervention pack labelled with the unique participant ID and containing 4 blister packs of 10 dispersible tablets each. Each individual blister pack will also have the same unique participant ID as the main intervention pack/ zip-lock bag.

Once the assigned zip-lock bag/ pack of intervention has been given to the participant, the study nurse/ medical officer will give one tablet of zinc (i.e. 5 mg elemental zinc) or placebo dissolved in 2.5 mL expressed breast milk or distilled water (particularly in non-breast fed), orally every 12 hours till discharge. At discharge the parents/ caregiver will be advised to continue with the intervention in the same dose to complete a total of 14 days of intervention

5.4. **Masking**:

The packing and appearance of the zinc sulphate and placebo tablets will be identical. In addition the zinc sulphate and placebo tablets will be similar in taste and appearance. This will ensure that the study team administering the intervention and ascertaining the outcome are unaware of group allocation.

Masking will be maintained during data analysis by coding the treatment allocation with two letters.

5.5. Study Procedures

5.5.1. Study setting:

The trial will be conducted over a period of 3 years and 6 months. We will recruit the infants in 7 centres: **5 centres in Delhi, India, Maulana Azad Medical College (MAMC) and associated Lok Nayak Hospital, Vardhman Mahavir Medical College & Safdarjung Hospital (VMMC & SJH), Chacha Nehru Bal Chikitsalaya (CNBC), Kasturba Hospital (KH) and Kalawati Saran Children’s Hospital (KSCH)**; and **2 centres in Nepal, Kanti Children’s Hospital (KCH), Kathmandu and Institute of Medicine (IOM), Kathmandu**. The selected hospital sites are secondary level hospitals with similar standard of care. This will make it easier to standardize study protocol across the hospitals. Each of the seven hospitals has a large patient load particularly of young infants with clinical severe infection. The implementation of the study will be coordinated by the Pediatric Biology Centre (PBC) at the Translational Health Science and Technology Institute (THSTI), in the National Capital Region (NCR), Delhi. THSTI will also coordinate the immunological studies to explain potential mechanisms of clinical severe infection and/or role of zinc in clinical severe infection. The other collaborating centres will be the Tribhuvan University, Institute of Medicine, Kathmandu, Nepal, Centre for International Health, University of Bergen, Norway (CHN-CIH), Innlandet Hospital Trust, Norway, Centres for Health Policy/Primary Care and Outcomes Research at Stanford University School of Medicine (CHP/PCOR) and Radbound University, Nijmegen, Netherlands. Tribhuvan University, Institute of Medicine, Kathmandu, Nepal will coordinate the implementation of the study at the Nepal hospital sites. CHN-CIH will play an advisory role and will help in establishing the

‘Data Management Centre’ for the study at THSTI and in data analysis. CHP/PCOR will collaborate with THSTI to measure the incremental cost effectiveness ratio of zinc supplementation as an intervention for clinical severe infection in infants <2 months. Radbound University will collaborate with THSTI, Institute of Medicine Tribhuvan University and CHN-CIH for the immunological studies to explain potential mechanisms.

5.5.2. Eligibility criteria:

5.5.2.a. Inclusion criteria for participants

We have adapted the inclusion criteria from the WHO IMCI[39] and IMNCI[40] to identify very sick infants with clinical severe infection.[41] Infants aged 3 days (48 hours and more) and up to 2 months attending the emergency services of the hospitals will be screened by a study nurse (at the Indian hospital sites)/ medical officer (at the Nepal hospital sites) for the following signs and will be eligible for enrolment if **<u>any</u>** of these five signs are present:

i. **Low body temperature/ hypothermia (<35.5°C)**
Young infants can also respond to infection by dropping their body temperature to below 35.5ºC.
A digital thermometer that measures to a minimum of 35ºC will be used to measure axillary temperature.

ii. **Movement only when stimulated**.
Young infants often sleep most of the time, and this is not a sign of illness. Even when awake, a healthy infant will usually not watch his mother and a physician/health worker while they talk, as an older infant or young child would.
A lethargic young infant who moves only when stimulated will *not be awake and alert when she/ he should be*. She/ He may be drowsy and may not awake after a disturbance.
If a young infant does not awake up during the assessment, the study nurse/ medical officer will flick the soles 2-3 times. She/ he will then look to see if the infant wakens and stays awake. If the young infant does not stay awake after some response, he is lethargic or said to have movement only when stimulated**.**

iii. **Stopped feeding well, defined as ‘poor feeding on observation’**

An infant is said to have poor feeding on observation if:
<u>Exclusively breast-fed infant has</u>:
- Poor attachment to breast
- Suckling is not effective

<u>Not exclusively breast-fed/ non-breast fed infant</u>**:**
On observation of a feed given with cup and spoon, the infant does not accept the feed as vigorously as before (as per the mother’s/ caregiver’s assessment)

iv. **Severe chest in drawing**:
When chest indrawing is present, the lower chest wall goes IN when the infant breathes IN.

Before the study nurse/ medical officer looks for chest indrawing she/ he must watch the infant to determine when the infant is breathing IN and when the infant is breathing OUT. If the infant's shirt was not lifted when she/ he counted the infant's breathes, the mother will be asked to lift it now. Study nurse/ medical officer will look for chest indrawing when the infant breathes IN. She/ he will look at the lower chest wall (lower ribs). The infant has chest indrawing if the lower chest wall goes IN when the infant breathes IN. Chest indrawing occurs when the effort the infant needs to breathe IN is much greater than normal. In normal breathing, the whole chest wall (upper and lower) and the abdomen move OUT when the infant breathes IN. When chest indrawing is present, the lower chest wall goes IN when the infant breathes IN. If the study nurse/ medical officer is not sure that chest indrawing is present, she/ he will look again. If the infant's body is bent at the waist, it is hard to see the lower chest wall move. The mother/ caregiver will be asked to change the infant's position so he is lying flat in her lap. If she/ he still does not see the lower chest wall go IN when the infant breathes IN, the infant does not have chest indrawing. *For chest indrawing to be present, it must be clearly visible and present all the time.* If the study nurse/ medical officer see chest indrawing when the infant is crying or feeding, the infant does not have chest indrawing. *If only the soft tissue between the ribs goes in when the infant breathes in (also called intercostal indrawing or intercostal retractions), the infant does not have chest indrawing.* In this assessment, chest indrawing is lower chest wall indrawing. It does not include "intercostal indrawing."

v. **Axillary temperature of ≥38.0°C on measurement,**

Fever with an axillary temperature ≥ **38.0°C** is uncommon in the first two months of life. If a young infant has fever, this may mean the infant has a severe infection. In addition, fever may be the only sign of a serious bacterial infection.

If the mother/ guardian gives a history of fever or low body temperature and on measurement the axillary temperature is indeed $\geq 38^0$C or < 35.5^0C respectively, the infant will be kept under observation in a thermo neutral environment for a maximum of 2 hours. Temperature measurement will be repeated every hour or SOS during the observation period. If there is a documentation of fever with axillary temperature of $\geq 38.0^o$C or low body temperature/ hypothermia at the end of 2 hours of the observation period, the infant will be said to have fulfilled this inclusion criteria.

The thermometer will be kept high in the axilla and the infant's arm will be held against her/ his body till there is a beep, before reading the temperature.

To avoid including neonates or infants with birth asphyxia or prematurity who may have the signs of chest indrawing &/or poor feeding &/or lethargy, a criteria based on duration of the sign/ symptoms has been included.

vi. **Infant should have been well at some point from birth till the current episode of illness.**

By 'infants being well' we mean infant should be active, feeding well and with no difficulty in breathing

Infants who fulfill the age criteria, have any of the signs i to v, fulfill criteria vi and whose parents/ guardians have given written informed consent for participation will fulfill the inclusion criteria for enrolment.

5.5.2.b. Exclusion criteria for participants

The infants fulfilling the inclusion criteria **will be excluded** if they have the following conditions:

i. **Weight at presentation < 1500 gm**

The infant's current weight will be measured on an electronic infant weighing scale after the treating physician has examined the infant and the vitals are stable.

All infants with weight of <1500 gm at the time of enrolment will be excluded.

ii. **Surgical or other conditions that interfere with oral/ nasogastric administration of study medication at admission.** For eg

a) Known structural defects, which interfere with feeding, e.g.

- esophageal abnormalities like esophageal atresia
- intestinal atresia and stenosis
- malrotation of the gut
- anorectal malformation like imperforate anus

b) Major congenital anomaly that interferes with **administration of study medication**. Eg. anencephaly, omphalocele

c) Infants requiring mechanical ventilation for respiratory failure, shock

d) Infants started on inotropic support

iii. **Infants requiring surgical intervention or admission outside of pediatric ward for management** eg Open fractures, large meningomyelocele, massive burns, etc

iv. **Infants requiring exchange transfusion on admission**

v. **History/ documented evidence of having received zinc in the last 48 hours**

We will examine the dose of elemental zinc that young infants in our area who receive multivitamin/micronutrient supplements (e.g. in the form of drops) are likely to ingest. If the likely dose exceeds 1 mg elemental zinc per day, babies receiving such supplements will not be included in the trial.

Any baby who has been started on zinc treatment for the current illness will not be included in the trial

vi. **History/ documented evidence of having received injectable antibiotics for the last 48 hours or more before admission**

Note: The screening for eligibility will be done rapidly (within 5-10 minutes) so that very little time is lost before starting the standard therapy for the 'clinical severe infection'.

Those infants who fulfill the criteria of 'clinical severe infection' and do not have any exclusion criteria but are very sick and not allowed oral/ nasogastric

intervention will not be enrolled immediately but will be observed during a 'stabilization period' when they will be observed by the study nurse/ medical officer every 4-8th hourly for a maximum period of 24 hours of stabilization. Infants who have been stabilized and allowed study medication orally, anytime within the 24 hour stabilization period; and who after stabilization continue to have at least one sign of 'clinical severe infection', no exclusion criteria and whose parents/ guardians have given written informed consent for participation will now fulfill the eligibility criteria.

5.5.3. Intervention and Co-interventions

5.5.3.a. Intervention: Identically looking zinc sulphate tablets (each dispersible tablet containing 5 mg of elemental zinc) and indistinguishable placebo tablets will be packaged in identically looking blister packs. Each blister pack will have 10 such dispersible tablets. Four such blister packs will be put in a zip-lock bag (intervention pack) for each enrolled participant. The zip-lock bag containing the four blister packs of 10 tablets each as well as each blister pack will be labeled with a 'unique serial number' which is the 'unique participant ID' according to the randomization list. The zip-lock bag containing the blister packs will be stored in a cool, dry place.

Once eligibility has been determined and informed consent for participation in the trial obtained, the infant will be randomized and assigned a unique 'participant ID' depending on the strata into which the infant is enrolled. The study nurse/ medical officer will take the intervention pack (zip-lock bag) labelled with the same unique participant ID and assign it to the enrolled infant.

Once the assigned intervention pack has been allocated and given to the participant, the study nurse/ medical officer will give one tablet of the intervention (i.e. 5 mg elemental zinc) or placebo dissolved in 2.5 mL expressed breast milk or distilled water (particularly in non-breast fed) orally to the infant. The study nurse/ medical officer will be responsible for giving the assigned intervention to the infant at enrolment and then twice a day (every 12 hourly) till hospital discharge. The infant will be **observed for 30 min after each dose of intervention** that is administered. In case the infant vomits within the observation period, the intervention will be repeated. A maximum of two attempts will be made to administer each dose. If an infant is unable to tolerate two consecutive doses due to vomiting, that dose will be skipped and the next and subsequent doses will be given to the enrolled infant.

In case the enrolled infant is not allowed oral feeds and therefore cannot be given the intervention orally, the intervention will be given using a nasogastric tube as will the required feeds. The 5 mg dispersible tablet of the study medication dissolved in 2.5 ml of expressed breast milk/ distilled water is minimal and can be given without any risk. The study staff will give the dissolved tablet orally every 12 hours till discharge. Each of the 4 blister packs will be saved and kept in the zip-lock bag even after all the tablets in the pack have been used. This will help in the pill count and help keep a tally of the study medication used.

At discharge the parent (s)/ caregiver will be advised to continue with the intervention in the same dose to complete a total of 14 days of intervention. A maximum period of 14 days is based on previous studies where beneficial effects of zinc therapy beyond the period of administration have been seen when given for 14

days. The parent (s)/ caregiver will be advised to repeat the intervention if their baby vomits within 30 minutes of administration of the intervention.

The dispersible tablets will be manufactured by a GMP certified company L.P. Rodael, France and procured from Nutriset, France (www.nutriset.fr) at a reasonable price. An international patent Rodael/Nutriset covers these tablets.
The study nurse/ medical officer will be responsible for administering each dose of zinc or placebo till discharge. This will ensure high compliance and small loss to follow-up till discharge.

In the few cases the enrolled infant develops necrotising enterocolitis or abdominal distension with tense abdomen or any such condition where the treating physician keeps the infant nil per orally and does not allow the intervention to be given either orally or by nasogastric route, the intervention will be withheld until there is clinical improvement. Infants who receive 50% or more of the projected doses in the first five days after enrolment will be included in the per protocol analysis.
Our strategy of monitoring enrolled subjects at 6 hourly intervals and close interaction of the caregiver and the study staff will ensure compliance and minimize the loss to follow up and withdrawals. It will also ensure that the babies receive excellent clinical care. Close supervision by the research officers (ROs)/ senior research officers (SROs) / clinical research coordinator (CRC) and site investigators will minimize protocol deviation.

5.5.3.b. Co-interventions: The treatment of enrolled infants of 'clinical severe infection' with standard antibiotics and other medications will be given based on standard procedures. These procedures will be standardized across the 7 centres. The antibiotics received by the enrolled participant will be documented in the case report forms along with the route, dose and frequency.

All other therapy prescribed to the enrolled infant by the treating physician like supplemental oxygen, intravenous fluids, multivitamins, etc. will also be documented in the case reporting forms along with the route, dose and frequency.

Care will be taken that the enrolled infant is not prescribed 'zinc' inadvertently particularly in the multivitamin drops or the creams for local application for perianal rash.

Note: Zinc is not recommended as standard treatment for diarrhea to infants< 2 months of age

5.5.3.c. Contraindications to subsequent doses of intervention

The infant requires life support or develops signs of 'critical illness', which precludes giving the intervention. The intervention will be restarted once the condition improves.

5.5.3.d. Procedures for Monitoring safety and participant compliance

Refer to sections 5.5.5.c and 8

5.5.4. Outcomes

5.5.4.a. <u>Primary Outcomes</u>

i. Case fatality, which is death due to any cause and at any time from enrolment until infant is in hospital for this episode of illness

ii. **Time to death until end of study period** defined as the time from enrolment to the time when an infant dies until 12 weeks from the day of enrolment in the study.

5.5.4.b. <u>Secondary Outcomes</u>

i. **Failure of treatment**. There is failure of treatment if one or more of the following events occur:

  a) Death at any time during hospitalization for the illness episode starting from the day of enrolment, , i.e. as primary outcome i., or

  b) Initiation of life support at any time after enrolment until hospital discharge. Need for life support will be defined as a need for ventilation or vasoactive drugs at any time after enrolment until hospital discharge, and/or

  c) A change in antibiotics for one of the following circumstances:

    i) Persistence of signs that indicate 'clinical severe infection' present at the time of enrolment anytime after 48 hours of enrolment and prior to discharge.

    ii) Worsening of existing signs or appearance of new signs of 'clinical severe infection' any time after enrolment and prior to discharge.

    iii) Reappearance of signs of 'clinical severe infection' that the infant initially presented with at time of enrolment anytime after 48 hours of disappearance and prior to discharge

ii. **Cessation of signs of clinical severe infection**: This will be defined as the beginning of a 48 hour period with none of the signs of 'clinical severe infection'.

iii. **Discharge**: Defined as discharge from the hospital.

iv. **Death at any time after discharge from hospital until end of study period which is 12 weeks from the day of enrolment.**

v. **Severe illness requiring hospitalisation at any time after discharge from hospital until end of study period which is 12 weeks from the day of enrolment.**

vi. **Cost-effectiveness analysis:** incremental cost effectiveness ratio of zinc supplementation as an intervention for 'clinical severe infection' in infants < 2 months.

*(Refer to **Annexure VII** :addendum protocol on extended cost effectiveness analysis [ECEA] for the expanded scope of a secondary objective on Cost-effectiveness analysis)*

vii. **Immunobiological readouts:** immunophenotyping of peripheral blood of infant at enrolment , 48-72 hrs of study and discharge

5.5.5. Data collection

5.5.5.a. Description of data arising from proposed research: The study nurses/medical officers will collect and safely store the data including patient eligibility, informed consent statements and clinical outcomes**.** The data will consist of baseline information on clinical and demographic characteristics of infants screened and enrolled in the study and details of 6 hourly progress including intervention, co-interventions, progress of clinical illness resulting in recovery, treatment failure or death during the hospitalization.

5.5.5.b. Data collection, including type, format, scale and standards for data: All the data will be collected in standard forms electronically/ paper CRFs at the seven sites by the study nurses/ medical officers supervised by the study physician (research officer/ senior research officer). The CRF template forms will be developed by the data management centre (DMC) and will consist of details of all the variables with standard definitions and labels.

5.5.5.c. Clinical Data Collection

Study nurses/ medical officers will examine the enrolled infants for all clinical features of ‘clinical severe infection’ every 6 hours or more often if clinically indicated, until discharge from the hospital. They will repeat the respiratory rate count if it is $\geq$ 60/min and the second of the two counts will be considered as the respiratory rate. They will also record nude weights at enrolment and every 24 hours till discharge. The research officers/ senior research officers (doctors) will oversee at least one daily monitoring conducted by each study nurse/ medical officer. The decisions for all outcome measures will be made by the RO/SRO in consultation with an experienced pediatrician (site investigator) based on the closely supervised 6 hourly monitoring done by the study nurse/ medical officer.

At the time of discharge the parents/ caregiver will be given a participant booklet and advise on the following:

i. To continue the intervention in the same dose of one tablet dissolved in 2.5 ml of expressed breast milk or distilled water given orally twice a day to complete a total of 14 days of the intervention. The participant booklet will have a log, which the parent/ caregiver will be asked to fill each time they administer the intervention.
ii. To repeat the dose in case the infant vomits within 30 min of having received a dose of intervention. In case there is vomiting within 30 min of repeat dose of intervention, the parent/ caregiver will be advised to skip that dose.
iii. They will be asked to make a follow up visit to the hospital site after they have completed the 14 day course of intervention (around day 15 of enrolment) and will be asked to get the zip-lock bag containing the 4 blister packs with them and the participant booklet. At the follow up visit data on the infant’s health will be collected and a ‘residual pill count’ will be made to check the compliance.
iv. They will also be advised that the study staff will be contacting them twice telephonically in the next 3 months to collect data on the infant’s health.
v. List of situations when the parent/ caregiver would need to contact the study team
vi. Contact numbers of the study team
vii. Advise on immunisation schedule

Once the enrolled infant with 'clinical severe infection' has recovered and been discharged from the hospital, the parents of the infant will be asked to get the infant to the hospital for a follow up visit (after completion of the 14 day course of intervention, around day 15 of enrolment). After this follow up visit they will be contacted on telephone at one and a half month and again at three months from the day of enrolment. During the post discharge follow up visit in the hospital and telephonic contact the following information will be collected using structured forms

i. Infants' health and well-being including the feeding pattern, any difficulties in feeding, frequency of urine and sleep pattern and routine immunization.
ii. Any illness in form of signs and symptoms of 'clinical severe infection' since last contact
iii. Any severe illness requiring hospitalisation since last contact. If yes, then details of hospitalization-diagnosis and duration of hospitalization. This will help us differentiate between accidents/ non-accidental indications for hospitalization.
iv. Death of infant. In case of death, validated verbal autopsy questionnaires will be used to essentially help us differentiate between death due to an accident or an illness/ non-accidental cause.

The study nurses/ medical officers will collect all this post discharge information. They will be trained in establishing telephonic contact with the parents and collecting all the relevant information as per the questionnaire.

Parents/ caregivers of infants will be encouraged to report any hospitalization within the 3-month study period post discharge to the study team through a visit to the hospital site or a call made to the team.

5.6. Standard Case Management

Each hospital site has a fixed antibiotic treatment algorithm and will follow the protocol strictly. Additionally, we will stratify the enrolled infant by hospital site and standardize the antibiotic treatment protocol across all hospital sites. Recommended doses of intravenous ampicillin or amoxicillin-clavulinic acid and an aminoglycoside (amikacin or gentamicin) will be given when infants come without antibiotic treatment (or on oral antibiotics) to the study hospitals, or a third generation cephalosporin (cefotaxime or ceftriaxone) and an aminoglycoside (amikacin or gentamicin) when infants have already been treated with these injectable antibiotics (for $\geq$ 48 hours) prior to admission. In case of suspected meningitis ceftriaxone or cefotaxime and amikacin will be started on admission. In case of suspected staphylococcal infection, cloxacillin or amoxicillin-clavulinic acid will be given with an aminoglycoside and in suspected staphylococcal meningitis vancomycin and an aminoglycoside will be administered. The duration of antimicrobial therapy will be 7-10 days, extendable to three weeks in meningitis. Intravenous fluids, temperature maintenance and oxygen will be provided as supportive therapy. The treating physicians who are also site investigators will be responsible for the medical treatment of the enrolled infants with clinical severe infection. The study staff will be responsible for clinical and safety monitoring as described later in the protocol.

5.7. Laboratory procedures

Bio-specimens

Study nurses/ medical officers will collect blood specimens at baseline (enrolment), at 48-72 hours of enrolment and at discharge.
The following is the schedule for peripheral blood collection for each enrolled participant at different time points:

i. At enrolment: 3-5 ml of blood will be collected intravenously for
   1. Blood culture by BACTEC
   2. Septic screen which includes
      i) total leukocyte count (TLC),
      ii) absolute neutrophil count (ANC),
      iii) C-reactive protein (CRP),
      iv) micro ESR and
      v) band cell: neutrophil count ratio (I:T ratio).
   3. Baseline serum zinc estimation
   4. Soluble markers of inflammation in serum
   5. Immunobiological studies
      i) Immunophenotyping of peripheral blood/ cell stimulation assays for measuring inflammatory response (cytokine levels)
      ii) DNA isolation and storage (for future methylation studies)
      iii) RNA isolation and storage (for future transcriptional studies)

A total of up to **5 ml of blood** will be collected at admission (enrolment). This will include 2 ml of the blood required for standard of care like **1 ml blood for blood culture** by BACTEC, and another **1 ml blood for septic screening** which includes TLC, ANC, mESR and I: T ratio. If CRP estimation is part of standard care management of infants with clinical severe infection, it will also be done real time. All investigations necessary for standard care will be done real time with techniques that use smaller volumes of blood.
The remaining 3 ml blood will be used for research objectives mentioned above.
1 ml blood will be used for isolation of plasma for estimation of **zinc, CRP and soluble markers of inflammation.**
1 ml fresh blood will be used for immunophenotyping of peripheral blood/ **cell stimulation assays** to be done using either fresh whole blood or by isolating PBMCs within 16 hours of collection. The culture supernatant from cell stimulation assays will be stored for cytokine level estimation.
The **remaining 1 ml** will be used for **DNA isolation for methylation studies (0.5 ml) and RNA isolation for transcriptional studies (0.5 ml)**.

Indian hospital sites: Blood samples for blood culture by BACTEC and septic screen will be processed and analysed at the respective Indian hospital sites or at an accredited laboratory in the close vicinity of the hospital.
Blood for the immunophenotyping will be transported on ice (4^0C); whereas blood for the cell stimulation assay studies will be transported at room temperature (37^0C) to the central laboratory at THSTI in the National Capital Region (NCR) at the earliest and definitely within 16 hours of collection and after the stimulation assays the culture supernatant will be stored in -70^0C to -80^0C deep freezers till analysis for analysis of inflammatory markers (cytokine levels).

The plasma for zinc, CRP and soluble markers of inflammation will be separated at the hospital site and then transported on ice to the central laboratory at THSTI where it will be stored at -70^{0}C to - 80^{0}C till analysis.

Nepal hospital sites: Blood samples for blood culture by BACTEC and septic screen will be processed and analysed at the respective Nepal hospital sites or at an accredited laboratory in the close vicinity of the hospital.

Blood for the immunobiological studies will be transported at room temperature (37^{0}C) to the central laboratory at Institute of Medicine (IOM) in Kathmandu, Nepal at the earliest and definitely within 16 hours of collection and after the stimulation assays the culture supernatant will be stored in -70^{0}C to - 80^{0}C deep freezers till analysis for cytokine levels.

The plasma for zinc, CRP and soluble markers of inflammation will be separated at the hospital site and then transported on ice to the central laboratory at IOM, Kathmandu, Nepal.

The samples for CRP, zinc estimation, soluble markers for inflammation and cell stimulation assays for cytokine levels will be processed on site/ at the central facility at IOM and transported for temporary storage in the -70^{0}C to - 80^{0}C deep freezer at the Institute of Medicine. These samples will be transported to the central laboratory at THSTI in India twice a year.

ii. At 48-72 hrs of enrolment: 3 ml of blood will be collected intravenously for
   a) Soluble markers of inflammation in plasma
   b) Immunobiological studies
      i) Cell stimulation assays for measuring inflammatory response (cytokine levels)
      ii) DNA isolation for methylation studies
      iii) RNA isolation for transcriptional studies

The study team will try to coincide the blood sampling for the 48-72 hours' time point with the routine sampling done in case of treatment failure or to see the progress in biochemical parameters of the sick infant. In case the participant does not require undergoing a routine sampling by 72 hours of enrolment, the study sample will be collected without any further delay.

iii. At discharge: 3 ml blood will be collected intravenously for
   a) Soluble markers of inflammation in plasma
   b) Immunobiological studies
      i) Immunophenotyping of peripheral blood/ cell stimulation assays for measuring inflammatory response (cytokine levels)
      ii) DNA isolation and storage (for future methylation studies)
      iii) RNA isolation and storage (for future transcriptional studies)

Plasma concentrations of zinc will be measured in a subset of the enrolled infants using standard procedures. The zinc concentration will be analyzed using a flame furnace atomic absorption spectrophotometer (Thermo Scientific) using standard techniques. Plasma CRP concentration will be measured using a commercial ELISA kit at the micronutrient lab at THSTI. Blood cultures will be done at enrolment using BACTEC (Becton Dickinson, Sparks, 152 MD, U.S.A.) at each hospital site or at an accredited laboratory in the close vicinity of the hospital. Blood collection for immunobiological

studies and for soluble markers of inflammation will be done at enrolment, 48-72 hrs and discharge.

5.8 Storage of leftover biospecimens

After all the processing and analysis of the collected bio specimens has been done for the stated objectives in the study, the excess or leftover bio specimen will be stored for future use. These samples may be used in the future to answer other important questions in medical research and/or for re-running the sample, if needed. The consent of the parent of the enrolled infant will be taken before any bio specimen is stored.

We will also seek permission from the concerned institutional ethics committee for the same. The identity of the enrolled infant whose sample has been stored will be protected by storing the samples without the participant's name or any contact information on them (de-identified). These specimens will be stored at the Pediatric Biology Centre of the Translational Health Science and Technology Institute, Govt. of India for a period of 10 years.

The participant will not receive any monetary or other benefits for the use of the stored specimens, nor will the institute sell or utilize the leftover samples for financial gain.

INVESTIGATION CHECKLIST

(Please tick and write date whenever any investigation is sent)

BIOSPECIMEN COLLECTION	0 HOURS<br>/ /	48-72 HOURS<br>/ /	DISCHARGE<br>/ /
	Volume of blood 3-5ml	Volume of blood 2-3ml	Volume of blood 2-3ml
**1.** Blood culture by BACTEC			
**2. Septic Screening**			
(i) TLC			
(ii) ANC			
(iii) CRP			
(iv) mESR			
(v) 1: T ratio			
**3.** Immunophenotyping/ cell stimulation assay for cytokine levels			
**4. Storage of bio specimens**			
(i) Zinc estimation			
(ii) Soluble markers of inflammation in plasma			
(iii) DNA isolation from blood for methylation studies			
(iv) RNA isolation from blood for transcriptional studies			

6. Supplies and Handling of Materials

Intervention: Zinc/Placebo

Zinc and placebo dispersible tablets will be manufactured by L.P Rodael (a French GMP certified company) for Nutriset. These tablets are covered by an international patent Rodael/Nutriset.. Each dispersible tablet of the intervention will contain 5 mg of elemental zinc as zinc sulphate. The placebo preparation will contain the same ingredients as the zinc sulphate preparation. The only way it will differ from the zinc sulphate preparation will be the zinc content. The zinc or placebo tablets will be similar in color, taste and appearance. They will be packaged in similar looking blister packs labeled with similar labels except for the 'unique ID' on each pack. Each blister pack will have 10 dispersible tablets of either zinc or placebo. Four such blister packs will be packaged in a zip-lock bag (intervention pack) for each enrolled participant. The zip-lock bag containing the 4 blister packs as well as each individual blister pack will be labeled with a 'unique serial number' which is the 'unique participant ID' and this will correspond to the randomization list.

The intervention will be shipped/ couriered by Nutriset to the coordination centres at THSTI, India / IOM, Nepal at room temperature with a data logger. In addition each shipment will contain the following information:

i. Title of study
ii. Protocol number: RCN/ZINCSEVINF/02/2015
iii. A caution that the drug is limited for investigational use
iv. Storage instructions: in dry and cool place
v. Strength of each dispersible tablet
vi. Total number of tablets in each blister pack
vii. Manufacturing license No
viii. Batch No
ix. Production date
x. Date of expiry
xi. Manufacturer's name, address, telephone number
xii. For oral administration only
xiii. Name of contact person

At the coordination centre a team not involved in the trial otherwise will do the labeling of the zip-lock bags containing the 4 blister packs and of each individual blister pack with the generated unique IDs. This team will receive a copy of the randomization sequence from the scientist/ statistician involved in generating the allocation sequence for the study.

Once the labeling is complete, the intervention packs will be stored in the intervention room at the Pediatric Biology Centre, THSTI, India / Institute of Medicine, Kathmandu, Nepal.

The required number of intervention packs will be transported from the coordination centre at THSTI/ IOM to the hospital sites in New Delhi, India and Kathmandu, Nepal at room temperature with a data logger. For the study purpose 28 doses of 5 mg tablet each will be utilized from the 4 blister packs (28 of the total 40 tablets) over a period of 14 days for oral administration and the remaining will be stored by the study team at the coordination centres for intervention accountability verification.

6.1. Labeling

The text of the label will be common to both the packs containing zinc or placebo. The label text will be based on masking requirements and have the following:

i. Protocol number
ii. Unique Enrolment/ Participant ID
iii. A caution that the drug is limited for investigational use
iv. Storage instructions: in dry and cool place
v. For oral administration only
vi. Name of contact person (site investigator)

6.2. Storage

The intervention (zinc / placebo) will be stored at room temperature in a dry and cool place. All the supplies will be stored at the central storage facility at the coordination centre at THSTI, India/ IOM, Nepal.

Each blister pack of intervention and its accompanying zip-lock bag will be labeled with a unique ID. Once an infant is enrolled in the study and assigned a unique participant ID, the corresponding intervention pack and each individual blister pack will be marked with the date and time of randomization.

Once assigned to a participant, the intervention will be kept in a dry place.

6.3. Clinical Supplies Accountability

The details of the intervention blister packs received at the coordination centre will be recorded at receipt twice- first from the manufacturer and then from the team that has labeled the blister packs and zip-lock bags. An inventory of the intervention packs will be maintained along with the remaining supplies by the ‘coordination centre team’.

This team will also maintain a log of intervention allocated.

The used intervention packs will be stored in a locked cupboard for intervention accountability verification by monitors. These packs will be stored till the study is complete and the data is published. The packs will be destroyed after instructions from the project management team (PMT).

6.4. Source Documents

The source documents for all the clinical data till the participant is enrolled in study and in the hospital till recovery and discharge, will be the eCRFs filled by the study staff after talking to the parent (s)/ caregiver and after examining the infant. The data entered into the eCRF shall be cross-verified by the Research Officer/ Senior Research Officer who will supervise the monitoring of the infant by the study staff at least once in a day and Quality Management team personnel visiting the site. In addition the laboratory forms from the hospital records will serve as source documents for the routine/ standard care laboratory reports. After discharge OPD slips, hospitalization summaries and laboratory reports will serve as source documents for some of the follow up data collection apart from the telephonically collected data on eCRFs. The database will not include

participant name or personal identifying information other than the participant ID, age of infant and initials. The eCRFs will have an identification form with the name of the participant, address, contact details, etc. This form will be used at the time of follow up, post discharge. This information will not be linked to the other data captured from the participant

eCRF should have the parameters defined in the source documents. Patient information except ID and Initials will be defined in the eCRF.

The laboratory data generated for the study will be imported into the database and the hard copies of the laboratory reports shall be the maintained at sites as source document. This shall be maintained securely in a cabinet, which will be strictly accessed by site study staff only.

7. WITHDRAWAL CRITERIA

Censorship/Withdrawal Criteria

The enrolled participants will be censored / considered to be withdrawn from the study in the following circumstances:

i. The family of the participant may wish to withdraw consent from the trial at any point during the study period, which is upto 12 weeks from the day of enrolment.
   <u>Note:</u> In case the family withdraws consent for blood samples to be taken for the purpose of the study but not for clinical monitoring and outcome assessment, the participant will not be considered to be withdrawn from study. Such a participant will be considered to be a protocol deviate.
ii. Participant may take discharge against medical advice and leave the hospital before outcome assessment (death or recovery). The study team will attempt to contact the parent (s) on telephone or by making a home visit and collect information on the primary outcome of death. But it may not always be feasible to follow up such participants for the outcome assessment. In case the study team is not able to establish contact with the study participant and is therefore not able to collect data on the outcome, the participant will be censored from study at this point.
iii. Completion of study period as defined by the protocol that is end of 12 weeks from the day of enrolment with or without outcome (that is either recovery from 'clinical severe infection' or death).

For participants who are withdrawn from the trial prior to completion of follow up, attempts will be made to assess the outcome and/or collect blood specimens as far as feasible, in addition to information on the reason for discontinuation.

8. SAFETY MONITORING

8.1. Adverse Events

8.1.1. Adverse Events (AE)

ICH E6 Good Clinical Practice Guidelines defines an **adverse event (AE)** as any untoward medical occurrence in a patient or clinical investigation participant, administered a pharmaceutical product that does not necessarily have a causal relationship with this treatment. An AE can therefore be any unfavorable and

unintended sign (including an abnormal laboratory finding), symptom or disease temporally associated with the use of intervention whether or not it is related to the intervention.
These adverse events may be **expected or unexpected**. The former is any adverse reaction whose nature and severity have been previously observed and documented for the intervention. Although for this intervention, there are no expected adverse events, the commonly reported adverse events will be documented. An **unexpected adverse event** is any adverse event not previously reported.

In this trial the following will be included as adverse events:

i. Regurgitation or vomiting within 30 minutes of administration of first dose of intervention
ii. Regurgitation or vomiting within 30 minutes of administration of subsequent dose/s? of intervention
iii. Vomiting any time during the study period
iv. Abdominal distension lasting > 24 hours any time during the study period
v. Change of antibiotics due to persistence of signs of 'clinical severe infection' anytime after 48 hours of enrolment and prior to discharge
vi. worsening of signs of clinical severe infection;
vii. development of signs of critical illness like unconsciousness, convulsions, unable to feed at all, severe dehydration, persistent vomiting
viii. appearance of new signs of clinical severe infection ;
ix. reappearance of signs of clinical severe infection after a 48 hours symptom free period

8.1.2. Serious Adverse Event (SAE)

A Serious Adverse Event (study specific) for the enrolled and hospitalized infant is defined as any untoward medical occurrence in the enrolled participant, that at any dose:

i. Results in death anytime during the study period
ii. Is life threatening (defined as a participant at immediate risk of death at the time of the event). For eg Initiation of life support
iii. Requires prolongation of existing hospital admission

Prolongation of hospital admission during the study period can be due to

a) development of signs of critical illness where life support is required like apnea, shock with low pulse volume and/ or delayed capillary refill time
b) development of necrotizing enterocolitis
c) appearance of new symptom or any unexpected event which could lead to prolongation of hospital stay like injection abscess or fall from cot leading to fracture or head injury

A Serious Adverse Event (study specific) for the enrolled infant who has been discharged from hospital but continues to be a part of the study till completion of the study duration (which is upto 12 weeks from day of enrolment is still in the study till 12 weeks from day of enrolment) is defined as any untoward medical occurrence in the enrolled participant, that results in:

iv. Death anytime during the study period
v. Serious illness leading to hospitalization

The study staff will be responsible for monitoring of all SAEs and AEs. SAEs to be reported to DSMB / Independent Ethics Committee within 24 hrs of occurrence by the responsible study site personnel.

The serious adverse events will be further refined and defined in the charter of the Data and Safety Monitoring Board (DSMB).

8.2. Relatedness to Intervention

Very likely/Certain: A clinical event with a plausible time relationship to administration of intervention and which cannot be explained by concurrent disease or other drugs or chemicals

Probable: A clinical event with a reasonable time relationship to administration of intervention; is unlikely to be attributed to concurrent disease or other drugs or chemicals.

Possible: A clinical event with a reasonable time relationship to administration of intervention, but which could be explained by concurrent disease or other drugs or chemicals

Unlikely: A clinical event whose time relationship to administration of intervention makes a causal connection improbable, but which could be plausible explained by underlying disease or other drugs or chemicals.

Unrelated: A clinical event with an incompatible time relationship and which could be explained by underlying disease or other drugs or chemicals.

Unclassifiable: A clinical event with insufficient information to permit assessment and identification of the cause.

8.3. Specification of Safety Parameters

The study nurse/ medical officer will be responsible for monitoring and recording of all AEs. All immediate adverse events following each dose of intervention (including regurgitation or vomiting within 30 minutes of administration of first or subsequent dose of intervention, vomiting any time during the study period, abdominal distension lasting > 24 hours any time during the study, appearance of new symptom, reappearance of signs of clinical severe infection after a 48 hours symptom free period, etc) will be documented for all infants. During the pre-defined 6 hourly clinical monitorings any development of signs of critical illness or any unexpected event which could lead to prolongation of hospital stay like injection abscess or fall from cot leading to fracture or head injury will be recorded in the CRF. The study nurse/ medical officer will also make three follow-up contacts after discharge of infant from hospital. The first follow up will be in the hospital and the subsequent two follow up contacts will be on phone. During each contact the study staff will collect data to ascertain SAEs, illness requiring hospital referral. In case the infant has an illness that warrants the infant to be seen by a pediatrician at the hospital, the infant will be advised to visit the hospital site for management.

Serious adverse events will be documented from the time of enrolment, throughout the study period, and reported to the coordinating centre IEC and the site IEC as per their requirements and to the sponsor/designee within 24 hours of being aware of the event. The coordinating centre IEC at THSTI/ site IEC will send the causality assessment of the SAE and compensation if any (in case of relatedness) to sponsor/ site PI within 30 days of the event.

SAE relatedness will be judged by the investigator/ designee and the DSMB who will have access to all relevant investigations, clinical assessments and management details.

8.4. Data and Safety Monitoring Board

There will be two DSMBs, one for the Indian hospital sites and one for the Nepal hospital sites. The DSMB for the Indian hospital sites will comprise of a pediatrician, intensive care physician, an epidemiologist and a biostatistician. The DSMB for the Nepal hospital sites will comprise of three members including a statistician and a pediatrician. One of the DSMB members can be overlapping in both the India and Nepal DSMBs. These DSMBs will be independent from the sponsor and will have no competing interests. The DSMBs will prepare a charter and decide *a priori* on study stopping rules, and will review SAE and AE reported in the study periodically. They will examine all infant deaths and other SAEs to decide if the study should be continued, based on the pre-decided stopping rules. After one third of the study participants have been enrolled and completed follow-up, the independent DSMBs will review the data and make recommendations concerning continuation, modification or termination of the study due to unexpectedly large beneficial effects or serious side effects.

Feedback to Data and Safety Monitoring Board (DSMB): A feedback form for the DSMBs containing relevant information will be prepared at the data management centre in consultation with the CRC, PBC PI, Nepal PI and the site investigators, (DMC) and sent to the DSMB at regular intervals.

8.4.1. Safety Advisor

A pediatrician will be designated as the safety advisor for this trial. All immediate adverse events and deaths will be reviewed by the safety advisor. Also, the team may obtain his/her opinion on any other issue related to the safety of enrolled infants. The safety advisor will be a part of the DSMB.

8.5. Independent study monitoring

An independent monitor chosen by and paid for by CISMAC will be responsible for the monitoring of the study. The monitoring plan will include predefined periodic visits to all recruitment sites by the independent monitors who will review every aspect of the study using standardized formats. The conclusions/recommendations arising from these monitoring visits will be communicated to the clinical research coordinator (CRC), PBC PI, Nepal PI and site PIs for corrective and preventive action (CAPA).

8.6. Adverse event reporting

Zinc has been shown to be safe in doses ranging from 5–45 mg per day when it has been used in both the treatment of acute diarrhea as short-term therapy and also as long-term supplementation in infants and young children in Asia, Latin America, and Africa. [18-25] All zinc salts (sulfate, acetate, and gluconate) have been found to be safe.[24] Zinc-treated infants in our own earlier trial [8] for treatment of probable serious bacterial infection did not demonstrate any adverse effects events. In addition to standard care provided by the hospital physicians the study physicians will examine the enrolled infants for any related adverse effects as given below at the time of giving the intervention and every 6 hours, or more often if indicated, until discharge from hospital and also till the infant completes the 14 days of the intervention.

All **serious adverse events (SAE)** of death, SAE other than death and initiation of life support (which are also the study outcomes) and **adverse events (AE)** as described above will be reported to the Ethics Committees, the DSMB (see below) and the sponsors of the study within the specified time period.

Serious adverse events will be documented from the time of enrolment, throughout the study period, and reported to the coordinating centre IEC and the site IEC as per their requirements and to the sponsor/designee and DSMB within 24 hours of being aware of the event and a due analysis report within 14 days of being aware of the SAE. The coordinating centre IEC at THSTI/ site IEC will send the causality assessment of the SAE and compensation if any (in case of relatedness) to sponsor/ site PI within 30 days of the event. SAE relatedness will be judged by the investigator/ designee and the DSMB who will have access to all relevant investigations, clinical assessments and management details. Information that will be collected includes event description, time of onset, investigator assessment of severity, relationship to study product, time of resolution of the event, seriousness, and outcome. All serious adverse events occurring in study participants will be documented appropriately regardless of relationship and followed up to adequate resolution or stabilization as judged by the treating physician and the investigator of the site.

All adverse events not meeting the criteria for “serious adverse events” will be captured on the appropriate case report form. All serious adverse events will be graded for intensity and relationship to the intervention. In addition each adverse event will be assessed to be serious (SAE) or not. Adverse events will be documented and reported to the coordinating centre IEC and the site IEC periodically and to the DSMB as per their requirement.

8.7. Follow Up After Adverse Events

All adverse events will be followed up till resolution or stabilization as judged by the treating pediatrician (site investigator) and the principal investigator. All SAEs will be followed up until satisfactory resolution or until the treating pediatrician and the principal investigator deems the event to be chronic or participant to be stable.

8.8. Trial Termination

The study may be stopped for ethical reasons at the recommendation of the institutional ethics committees of any of the partner institutions/ hospital sites or for safety reasons

at the recommendations of the DSMB, or sponsor /funding agencies in which case no further dose of the intervention will be given but follow up of enrolled participants will continue till the end of study period as scheduled.

9. Ethical Issues and Approvals

9.1. Ethical issues:

i. The drug which is the study intervention (elemental zinc given as zinc sulphate tablets orally in the dose of 10 mg per day for 14 days) is already approved for use in infants with diarrhea. This study extends the use of the intervention to other illnesses at the same dosage of 10 mg per day and for the same duration. Therefore there is no ethical issue about its use. Additionally, most infants with diarrhea in this age group of less than 2 months have systemic illness.

ii. As mentioned above, the study intervention (elemental zinc given as zinc sulphate tablets orally in the dose of 10 mg per day for 14 days) is already approved for use in infants with diarrhea. Therefore, as per the Circular (File No. 12-01/14-DC (pt. 47)) from the DCGI dated 10-11-2015 and the Gazette Notification (Regd. No. D.L.-33004/99) from the Ministry of Health and Family Welfare dated 16-03-2016, since this trial is not being done for claiming permission of New Drug for marketing as per Drugs and Cosmetic Rules, permission from DCG (I) shall not be required provided that the trial has been approved by the respective Ethics Committee. (Circular and Gazette Notification attached as **Annexure II**).

9.2. Ethical Approvals

The Investigators will initiate the study at each site only after securing approval from the Institutional Ethics Committees of the hospital in which the study is to be carried out as well as from THSTI IEC and REC West in Norway for the Indian sites and likewise from NHRC Nepal and REC West in Norway for the Nepal sites.

10. STATISTICS

10.1. Sample Size

Based on the most recent annual audits undertaken in the district hospitals in India, the case fatality risk in infants < 2 months with clinical severe infection is expected to be 10%. Assuming a 10% loss to follow-up, we will need to recruit 4,140 infants (2,070 in each group) to identify a clinically important (≥30%) risk reduction of death by administering zinc, with 90% power and 5% confidence.

With the proposed sample size we will have sufficient power to detect a difference in time to death from enrolment until 12 weeks post discharge. Although a secondary outcome, with the proposed sample size, we will be able to detect even a 17% relative risk reduction in treatment failure between the two groups at 90% power assuming the risk of treatment failure in the placebo arm to be 25%.

10.2. Analysis

10.2.1. Plan of analysis

10.2.1.a Plan of analysis for the primary outcome:

The **primary outcome** is **case fatality**. Using statistical software for analysis (STATA), the proportion of children who die will be calculated in the two study (zinc and placebo) arms. This will give us the risk in the two study arms. Relative risks (RRs), risk differences (RDs) and numbers needed to treat (NNT), and their corresponding 95% confidence intervals (CIs) will be calculated. This will be done using an intention-to-treat approach where all infants assigned into the zinc or placebo group will be analyzed. There will be a systematic registration of all baseline characteristics. In case the baseline variables associated with the primary outcome are unevenly distributed between the two study arms, we will adjust the RRs and RDs/NNT for these baseline imbalances, using generalized linear models of the binomial family with log and identity links, respectively.

10.2.1.b Secondary outcomes:

Likewise, we will compare the risks in the intervention and placebo arms of the trial, for the secondary outcomes like **treatment failure**, **death at any time after discharge from hospital until end of study period which is 12 weeks from the day of enrolment, severe illness requiring hospitalisation at any time after discharge from hospital until end of study period**.

For the secondary outcome of treatment failure we will compare the risk in the intervention and placebo arms of the trial using

i. Composite definition of treatment failure

ii. Cause specific treatment failure.

   Cause of treatment failure will be assigned by the worst outcome and the first cause of failure.

iii. Treatment failure where suspected nosocomial infections will be excluded. Nosocomial infections will be suspected when antibiotics are changed for

   a. Persistence of signs of 'clinical severe infection' beyond 168 completed hours,

   or

   b. Worsening of signs of 'clinical severe infection' after 168 completed hours

   or

   c. Reappearance of signs of 'clinical severe infection' 48 hours after a symptom free period

These secondary outcomes will be analyzed the same way as the primary outcomes. If an outcome-event can occur more than once, we will adjust for the repeated observations in statistical models. The secondary outcome of severe illness requiring hospitalization will also be analyzed in Poisson or negative binomial regression models (allowing for more than one event per child).

Percent efficacy will be calculated using one of these formulas: 100×(1-relative risk) or 100×(incidence rate ratio), as appropriate.

10.2.1.c Time-to-event analyses will be done for the following **primary and secondary outcomes**:

Time to death until end of study period defined as the time from enrolment to the time when an infant dies anytime until 12 weeks from the day of enrolment in the study**:** This analysis will consider the event as death at any time after enrolment until 12 weeks from the day of enrolment into the study. Again, this will be done using an intention-to-treat approach where all infants assigned into the zinc or placebo group will be analyzed. Infants where the parent/ guardian withdraws consent for continuation in the study or for other reasons cannot be followed until 12 weeks after enrolment in the study will be censored at the time point that they were lost to follow up. Data on these lost to follow up infants till the time of censoring will be considered during the analysis. We will use a Cox proportional hazards model to compare the time to death any time after enrolment until end of study period, which is 12 weeks from the day of enrolment between study groups. The hazard ratio (HR) and their corresponding 95% confidence intervals (CIs) will be calculated.
Time to death: The event will be death at any time after enrolment during hospitalization for the illness episode. Infants where the parent/ guardian withdraws consent for continuation in the study or for other reasons cannot be followed until recovery and discharge will be censored. This will also be done using an intention-to-treat approach where all infants assigned into the zinc or placebo group will be analyzed. We will use a Cox proportional hazards model to compare the time to death until hospital discharge between study groups and will calculate the relative hazard of death during hospitalization between the intervention groups in (i.e. comparing time until death during hospitalization).
Time to cessation of signs of clinical severe infection: The event will be cessation of signs of clinical severe infection. Infants, where the caregiver withdraws consent for continuation or for other reasons cannot be followed further, will be censored. Irrespective of whether treatment failure has occurred, we will use a Cox proportional hazards model to compare the time to cessation of signs of clinical severe infection between study groups.
Time to failure of treatment: This analysis will consider the event as treatment failure. Censoring infants who withdraw consent to continue in the trial we will use a Cox proportional hazards model to compare the time to treatment failure between study groups.
Time to discharge: This analysis will consider the event as discharge. Censoring infants who withdraw consent for continuation in the trial, we will use a Cox proportional hazards model to compare the time to discharge between study groups.

Percent efficacy in time-to the events analyses is 100×(1-hazard ratio).

Like antibiotics, zinc is unlikely to exert an effect before 24 hours of administration. All of the above analyses will therefore be repeated where the outcomes are redefined to occur only when the event takes place after 24 hours of administering the first dose of zinc.

10.2.1.d Analyses of laboratory parameters:

Serum zinc concentration and **immunobiological readouts** at recovery and the change from baseline to recovery will be compared between the two groups using Student's t-test or, if need to adjust for potential confounders, with linear regression.

10.2.1.e Subgroup analyses

Subgroup analyses to study effects of zinc on primary and secondary outcomes will be done (i) within each hospital, (ii) for whether the child had diarrhea (on admission) to assess whether its presence at enrolment modifies the effect of zinc [8] (iii) in those less than 7 days old and (iv) presence of sepsis, either 'culture positive' (true pathogen detected in blood culture) or 'clinical sepsis' defined by a negative blood culture but a positive septic screen with presence of any two of the following parameters: total leucocyte count <5000/cmm; absolute neutrophil count <1500/cmm; band cell: neutrophil ratio >0.2; micro ESR >15mm at 1st hour and C-reactive protein levels >1mg/dl (adapted from Center for Disease Control and Prevention criteria [42]). Heterogeneity of RRs and RDs will be estimated using interaction terms in generalized linear models of the binomial family with log and identity links, respectively.

10.2.1.f Cost effectiveness Analysis (CEA):

We will measure the incremental cost effectiveness ratio of zinc supplementation as an intervention for clinical severe infection in infants < 2 months. We will adopt an ingredient-based costing approach to value the resources used. This will involve the following steps: 1) categorize types of resources; 2) quantify # units of each resource used for each subject; 3) identify the unit cost for each resource type; 4) multiply unit costs by associated quantities and sum to generate a total cost for each subject. We will collect information on the quantity of each resource category used in 6 hourly intervals for each subject, when the study nurse records the main study data and from hospital records. Data collection will continue until hospital discharge or death. We hypothesize that the zinc treatment will reduce the probabilities of: 1) needing ICU care; 2) needing a change in antibiotics; 3) needing closer monitoring for longer periods 4) death. Therefore, we will ensure that resource categorization is refined enough so that we can examine these potential effects (Zinc, Hospital days, ICU days, Medicines given, Procedures, Lab tests, Imaging studies, Specialist consults, Doctor time, Nurse time, Other healthcare worker time, Caregiver time, Caregiver direct costs etc.) at each monitoring point. We will develop unit prices to value resources in each category via a separate data collection effort. Valuation of resources in the healthcare context is often complicated because market prices are not readily and publically available. To address this challenge, we will use a multi-pronged approach. We will use Rashtriya Swasthya Bima Yojana (RSBY) a national health insurance scheme that maintains a public list of reimbursement rates for inpatient services. Using these and other sources (e.g., unit prices paid by the government for bulk pharmaceutical purchases), we will develop likely ranges of the economic value of each unit of each type of resource. The effectiveness of the intervention potentially involves both reductions in mortality and morbidity, which will

be combined into a single metric, the quality adjusted life year (QALY). For morbidity, since children are held in hospital until they recover sufficiently, we will assume similarity between the two study arms at discharge. Therefore, we will assess quality based on the time each child spends in the regular ward (i.e., higher quality) versus the ICU (i.e., lower quality). Quality weights for these time periods will be derived from existing literature. However, because hospital stays may be shortened positively by quicker recovery or negatively via mortality and because the loss of life represents many years of lost life expectancy, we will compute the remaining quality adjusted life expectancy for each child based on current age, sex, state of residence, and residual morbidities using Sample Registration System life tables. Mean costs and mean QALYs for each study arm will be computed. If there are observable differences in baseline characteristics we will use appropriate statistical models (e.g., limited dependent variables since costs cannot be negative) to compute adjusted mean costs and QALYs. We will then compute the difference in mean costs and mean QALYs comparing the zinc arm to the standard-of-care, taking the ratio of these differences to estimate the incremental cost-effectiveness ratio (ICER) for zinc treatment.

*(Refer to **Annexure VII** :addendum protocol on extended cost effectiveness analysis [ECEA] for the expanded scope of a secondary objective on Cost-effectiveness analysis)*

10.2.2. Selection of Subjects to be Included in the Analysis

The compliance in our previous study was close to 100% as the study staff administered each dose of the intervention till the end of the study. The small proportion of 6% who withdrew from the study took the intervention as long as they participated in the trial. Adopting a nearly identical strategy in the proposed trial, where the study nurse/ medical officer will administer the intervention to the enrolled infant twice a day till discharge from the hospital, we expect to have a similarly high compliance and small loss to follow-up**.** Our strategy of monitoring enrolled infants at 6 hourly intervals and close interaction of the caregiver and the study staff during the hospital stay will ensure compliance and minimize the loss to follow up and withdrawals. Close supervision by the RO/ SRO/ CRC and the PI and CPIs will reduce protocol deviations to a minimum.

At the time of discharge the parent (s) / caregiver will be advised to administer the intervention to the infant in the same dose twice a day to complete a total of 14 days. A participant booklet with a log will be provided to the parent (s) / caregiver on discharge and they will be asked to fill the log each time they administer the intervention. The parent (s) / caregiver will be asked to get the infant to the hospital for a follow up visit (after completion of the 14 day course of intervention), around day 15 of enrolment and asked to get the intervention pack with them and the participant booklet. At the follow up visit, data on the infant's health will be collected and a residual 'pill count will be made to check the compliance. After this follow up visit they will be contacted on telephone at one and a half month and again at three months from the day of enrolment.

10.2.2.a Intention-to-treat (ITT) analysis

i. All infants once found eligible and assigned an intervention and in whom the outcome is known (case fatality/ treatment failure/ discharge/ serious illness requiring hospitalization after the discharge until end of study period) will be included in the ITT analysis.

ii. Time-to-event analyses

This analysis will consider the event as death/ discharge/ treatment failure/ cessation of signs of clinical severe infection. This will be done using an intention-to-treat approach where all infants assigned into the zinc or placebo group will be analyzed. Infants where the parent/ guardian withdraws consent for continuation in the study or for other reasons cannot be followed until outcome will be censored at the time point that they were lost to follow up. Data on these lost to follow up infants till the time of censoring will be considered during the analysis. We will use a Cox proportional hazards model to compare the time to event between study groups. The hazard ratio (HR) and their corresponding 95% confidence intervals (CIs) will be calculated.

10.2.2.b Per protocol analysis

i. **Case fatality anytime from enrolment until discharge**

Infants who receive 50% or more of the projected doses in the first five days after enrolment will be included in the per protocol analysis

ii. **Treatment failure**

Infants who receive 50% or more of the projected doses in the first five days after enrolment will be included in the per protocol analysis

iii. **Death at any time after discharge from hospital until end of study period, which is 12 weeks from the day of enrolment**

Infants who receive 50% or more of the projected doses in the 14 days from enrolment will be included in the per protocol analysis

iv. **Severe illness requiring hospitalization at any time after discharge from hospital until end of study period**

Infants who receive 50% or more of the projected doses in the 14 days from enrolment will be included in the per protocol analysis

11. DIRECT ACCESS TO SOURCE DATA/ DOCUMENTS

Trial-related monitoring, audits, IEC review, and regulatory inspection(s) will be permitted by providing direct access to source data/documents to sponsors or monitors and regulators.

12. QUALITY CONTROL AND QUALITY ASSURANCE

Misclassification will be minimized by employing strict definitions of the outcomes, developing clear and concise Standard Operating Procedures (SOPs), initial training before study initiation and repeated training during study implementation, as well as regular and rigid supervision of study staff.

Quality control will be implemented right from the recruitment stage where we will follow very stringent criteria for recruiting staff for the research related activities. We will ensure that the personnel recruited for the study have the desired qualifications and

experience to take up their responsibilities. All levels of the staff will receive training consistent with the Good Clinical Practices guidelines (GCP) and in the study protocol. The staff will then be trained in the study protocol specific activities as per GCP. The Clinical Research Coordinator (CRC) will ensure adherence to implementation of the strategy and along with the, Senior Research Officers (SRO)/ Research Officers (RO) will ensure quality control through regular checks on all activities being performed in the study, such as screening of infants aged 3-59 days coming to the hospital out patients department (OPD) or emergency department, taking written consent from parents/ guardians, enrolment of eligible infants, administering the intervention/ placebo at enrolment and then every 12 hourly until discharge and advising the parents/ caregiver to continue with the intervention at home after discharge in the same dose to complete a total of 14 days, follow up of enrolled infants every 6 hourly, assessing for and documenting outcomes, documentation and management of adverse and serious adverse events, collection, immediate processing, temporary storage and transportation of blood samples at the designated time points, calibration of clinical and laboratory equipment like infant weighing scale, infantometer, incubator, centrifuge, refrigerator, deep freezers, pipettes, etc.

The CRC will assist the investigators in developing clear and concise case report forms (CRFs), responsibility logs, equipment calibration logs, training logs, and standard operating procedures (SOP) for each research related activity like screening with clear objective definitions for determining eligibility, taking informed consent, enrolment, administering the intervention/placebo, follow up, clear objective definitions for assessing outcomes, documenting and management of serious adverse events, reporting of SAEs following the guidelines, form filling guidelines, calibration of equipment, laboratory procedures like collection of blood, immediate processing, transportation and storage of samples.

Initial training and then repeated training during study implementation will be provided to the study staff on each relevant study activities. In order to ensure that the standardized protocol is followed at all sites, standardization exercises will be done within each site and then between different sites at regular defined intervals.

In addition, the following QC/QA procedures will be ensured:

- Maintaining a paper trail whenever there are changes in the CRFs and the SOPs
- Proper storage of the consent forms under lock and key with restricted access.
- A systematic registration of all relevant baseline characteristics will enable us to adjust for relevant confounders, should variables associated with the outcomes be unevenly distributed between trial arms by randomization.

  Quality control (QC) of laboratory samples will be done using a standard protocol

12.1. Staff Training

The components of the study specific training are:

- Protocol and therapeutic area training
- Training on eCRF filling guidelines
- Class room training for determining eligibility, taking informed consent from parents/ guardian, enrolling infant, assigning intervention, administering intervention, ascertainment of outcomes, adverse events, reporting of adverse events, blood sampling, calibration of site equipment, documentation practices.

- Role plays for obtaining written informed consent
- Training for all possible emergency scenarios by group discussions and mock exercises

12.2. Site Monitoring

<u>Site readiness monitoring visit:</u> After appropriate ethics approvals by all the relevant Institutional Ethics Committees (IECs) are available (and the final protocol has been amended as required by IECs), a **'site initiation visit'** will be conducted before the first participant is enrolled at that site. The participants cannot be enrolled until occurrence of such visit and its documentation. During this site visit, the requirements of protocol procedures, and all logistical issues will be discussed at length. During the visit, the monitor will complete a standard checklist that covers key aspects to indicate the readiness of the site. When the monitor has shared site readiness report with the PMT, the PI or the PMT member designated by the PI will together with the monitor develop a plan of action to address any problems identified during the visit. The PI is responsible for the implementation of the plan and addressing all problems before recruitment of the first study participant.

Regular monitoring and audit will be conducted at the hospital sites and the coordinating centre by external agencies. CDSA (Clinical Development Services Agency), an extramural centre of THSTI will be responsible for quality monitoring in the study sites in India. CISMAC will coordinate the quality monitoring at the Nepal hospital sites. After the study is initiated, the study monitor will be in regular contact with the sites to obtain information on the performance of the study. These contacts will be scheduled to take place at **regular intervals**. Subsequent to start of recruitment, **routine-monitoring visits** would occur after prior appointment with the investigators.

The investigator and his/her staff are obliged to devote a suitable amount of time and an appropriate place for the monitoring visits. Monitoring visits will include, but will not be limited to, review of files, intervention accountability records, eCRFs, informed consent forms, laboratory reports, and protocol compliance. During each visit, the monitor will review the electronic case report form (CRF) of each participant in the study with regard to completeness, thoroughness and compliance with the protocol. eCRFs will be filled up for all the participants who receive study intervention by study staff. For those participants who are dropped before the completion of the study, the reason for their termination must be specified. Such reports must be signed by the site Investigator or his/her designee and submitted to the PMT in the same manner as completed reports.

In addition, at a minimum, the original participant data will be reviewed to ensure that:

- participant informed consent is incorporated;
- inclusion/exclusion criteria are properly followed;
- eCRF data are consistent
- all relevant clinical and laboratory findings and concomitant medication are documented in the eCRFs;
- quantity and dosing schedule of concomitant medication is documented in the paper CRF/ eCRFs;

- quantity and dosing schedule of the Investigational/Comparator Product is in accordance with the protocol;
- all relevant information (e.g., any adverse event) has been recorded in the appropriate place in the eCRF/ paper CRFs;
- All AEs and SAEs have been managed as per standard case management
- the Investigational/Comparator Product is being stored correctly, and its supply is being properly accounted for;
- Incorrect or illegible entries in the CRFs would be submitted to the site investigator or his/her designee for correction.

The monitor will retrieve completed CRFs during the regularly held monitoring visits. The study monitors will meet with site investigator or his/her designee to discuss any problems and actions to be taken, and document visit findings and discussions. When the monitor has shared ‘monitoring visit’ report with the PMT, the PI or the PMT member designated by the PI will together with the monitor and the site investigator/ designee develop a plan of action to address any weaknesses/challenges identified during the visit. During the study period, the responsible CDSA staff will be available to answer questions with regard to the performance of the study.

12.3. Auditing

In addition to the above outlined monitoring visits, the participating institutions may be audited. This audit may be carried out by representatives of monitoring agency or by the responsible regulatory authority (ies). Such an audit would be done to review whether the data has been properly recorded in the interim or final report and whether the performance of the study is in accordance with the protocol, and other relevant guidelines. Participant confidentiality will be maintained at all times. The investigator will inform monitoring agency immediately if an audit has been requested by a regulatory authority/committee /agency.

13. ETHICS COMMITTEE CLEARANCES

The Investigators will initiate the study at each site only after securing approval from the Institutional Ethics Committees of the hospital site in which the study is to be carried out as well as from THSTI IEC and REC West in Norway for the Indian sites and likewise from NHRC Nepal and REC West in Norway for the Nepal sites. Appropriate written informed consent will be obtained from the parents or guardians of each participant after explaining the nature of the study, expected benefits and possible adverse effects of the study intervention as explained in the Participant Information Sheet & Informed Consent Form.

Participant confidentiality will be maintained at all times during the conduct of the study, and after completion of the study.

India: Approvals will be obtained from the institutional ethics committees of THSTI and the hospital sites.

Nepal: approvals will be obtained from Nepal Health Research Council (NHRC) and the ethics committees of the hospital sites.

14. CONSENT FOR PARTICIPATION

Informed written consent will be taken from one or both parent(s)/ guardian of the participating infant before enrolment into the study. The study nurse taking the informed consent will speak to the parent(s)/ guardian in the language they understand. She/ he will provide the parent (s)/ guardian with the participant information sheet that will be in the local language and will detail the focus of the study along with the associated risks and benefits of the trial. The study nurse will first explain to the parent (s)/ guardian verbally about the study before giving them the participant information sheet. She/ he will explain why the study is being done, how it is being done. She/ he will emphasize that the infant will continue to get the standard care (standard antimicrobials and other supportive care) that he/she should get for his/ her illness.

If the parent (s)/ guardians agree for participation in the study then in addition to the standard antimicrobials the infant will get either zinc or placebo as the intervention. The only reason for giving a placebo, which is a totally harmless product, is that by using a placebo we will be able to measure whether zinc is effective in treating the serious infection that their baby has in an unbiased way; if those who get zinc fare better then it means that zinc is effective as an adjunct to the standard treatment for this disease/infection. The parent(s)/ guardian will be explained that neither the doctor nor the parent(s)/ guardian will know whether zinc or placebo is being given; this is done so that no one is influenced by this foreknowledge and standard high quality care is provided to all babies.

They will also be explained that a small quantity of blood will be taken for routine investigations for standard management and some specific markers of the infection and immune status and for zinc estimation. It will be emphasized that their baby will be constantly monitored in the Pediatric ward and all cost of the medication will be taken care of by the study. They will be informed that the infant will be kept in the hospital till complete recovery and will get all medications from the hospital/ study.

The parent (s)/ guardian will be informed that after all the processing and analysis of the collected bio specimens has been done for the stated objectives in the study, we would like to store the excess or leftover bio specimen for future use. These samples may be used in the future to answer other important questions in medical research and/or for re-running the sample, if needed. The consent of the parent of the enrolled infant will be taken before any bio specimen is stored.

The identity of the enrolled infant whose sample has been stored will be protected by storing the samples without the participant's name or any contact information on them (de-identified). These specimens will be stored at the Pediatric Biology Centre of the Translational Health Science and Technology Institute, Govt. of India for a period of 10 years.

It will also be emphasized that there is enough evidence to show that zinc is a very safe micronutrient. It will be explained to the parent(s)/ guardian that participation in the study is voluntary and even if they agree for participation initially they are free to leave the study whenever they desire. This decision will in no way affect the standard care that they receive at the hospital.

Once the participant information sheet is provided to the parent(s)/ guardian they will read it themselves. Illiterate parents will be encouraged to bring along a literate neighbour or relative as a witness. The parent(s)/ guardian will be given an opportunity to ask any questions that they have, which will be answered by the person administering consent or the study supervisors. The parents will be asked a set of questions to assess their understanding of the consenting procedure and the study. The consent process would be repeated if the parent(s)/ guardian have not understood clearly.

Once the person administering the consent is satisfied that the parent(s)/ guardian have understood the consent form and are willing for their infant's participation in the study if found eligible, she/he will ask the parent(s)/ guardian to sign (or put a thumb impression in case illiterate) the participant informed consent form. If the parent(s)/ guardian are illiterate, in addition to the thumb impression, a literate impartial witness will be asked to sign the participant informed consent form.

If the consent is refused, the infant will not be included and the cause for not including will be recorded. The parent(s)/ guardian will be assured that all services and treatment that the infant will receive at the hospital will continue and nothing will change even if they choose not to participate in the study.

The filled participant informed consent forms will be filed and locked in a cabinet at the hospital site. A duplicate original signed copy of the participant informed consent form will be given to the parent(s)/ guardian. The participant's contact information will be kept in a locked cupboard at the hospital site while the study is ongoing and shifted to a locked cupboard holding the study documents at the coordinating centre once the study is completed.

Serum specimens of those participants whose parent(s) have refused for analysis of the samples for research purpose (after blood sampling was done)

The laboratory technician in the presence of the CRC/SRO/PI will discard all aliquots of the serum specimens of these participants. A list of these participant IDs will be typed, dated, initialed by those who witnessed the procedure and noted in the laboratory file.

Participant confidentiality

Any information obtained about the enrolled infant in the course of the study constitutes confidential information. This information will be used solely for the purposes of the trial and will not be shared with anyone that is not connected with the study. All the clinical and laboratory data collected from the enrolled infants will be coded with a unique serial number/ unique enrolment ID/ participant ID. All research records will have very restricted access. In the event of any publication or presentation resulting from the study, no personally identifiable information will be shared.

All records with the participant identification information will be kept in a locked cupboard in the coordinating centre and will be accessible to only the investigators. All CRFs will only have enrolment/ participant IDs, participant initials, age and no other identifiers. Data will be provided only to the DSMB, the IECs, auditors and the funding agency, if deemed necessary.

Incentive for participation

The principal and site investigators will ensure that no verbal or financial incentive is used to coerce families to participate.

15. DATA HANDLING AND RECORD KEEPING

15.1. Data Management

The Data Management Centre (DMC) at THSTI will manage the data.

i. **Description of data arising from proposed research:** The data will consist of baseline information on clinical and demographic characteristics of infants screened and enrolled in the study and details of 6 hourly progress including intervention, co-interventions, progress of clinical illness resulting in recovery, treatment failure or death during the hospitalization and details of any morbidity in the following 12 weeks from the day of enrolment.

ii. **Data collection, including type, format, scale and standards for data:** All the data except for laboratory data arising from the research laboratory, will be collected by the study nurses/ medical officers in eCRF designed on tablets placed at the seven sites and supervised by the study physician (research officer/ senior research officer). The laboratory data will be entered either in paper CRFs or entered in the desktop application and shall be either imported directly into the eCRF or manually entered at Data Management. The forms will be developed by the DMC and will consist of details of all the variables with standard definitions and labels. Consistency and range checks will be inbuilt into the “data entry screens” such that these checks are applied during entry itself. For data where the discrepancies are generated , the queries shall be available to the site staff on tablets for resolution. The DMC will generate manual queries via web while performing quality control of data.

All the study site/ Monitors/ Quality Management staff shall be trained for eCRF filling guidelines by the Data Management personnel. The relevant eCRF flow Power Point presentation shall also be provided to all the study site staff for reference.

Data Management personnel shall be responsible for providing adhoc reports to the relevant stakeholders on a bi-weekly or weekly basis or as and when required.

iii. **Data security and storage:** Data will be captured offline and saved locally on the tablet once the user hits the save button. The saved data shall be securely synced to the web server once the user hits the sync button in a prefixed time schedule daily; and for the sites where internet is intermittent or temporary internet outage, the user can sync the entered data once the connection is up or the user is in the internet zone.

iv. The data is securely synced into the server, as the sync process will be done through SSL. The data will be sent to the web server in encrypted packets which will be decrypted only by the web server receiving the data. The application and Database server shall be maintained in a cloud based / dedicated server located at THSTI. Daily and monthly backups to be scheduled as per the PBC server management plan.

v. **Data tracking, cleaning, and quality checks:** Data Management shall pull out the data from the web server and shall maintain enrollment status tracker. Data Management shall extract the data from the web server every fortnight and perform the QC activities as per the Quality Control SOP. All the queries shall be raised manually on the web, and shall be transmitted to the site at the time of data sync.

vi. **Feedback to Data Safety Monitoring Board (DSMB):** A feedback form for the DSMB containing relevant information will be prepared by the data management team in consultation with the project management team and the site investigators and sent to the DSMB at regular intervals.

15.2. Record Retention and Archival

Daily data backups shall be maintained in 2 parts. One will be available on the server date wise and the second copy of all the monthly data shall be maintained in an external Hard Disk Drive (NAS Drive). A copy of the same shall be taken every month on another external hard drive and shall be kept within a fire proof cabinet with the study Investigator/ Head Data Management. The study data shall be archived post submission of Complete Study Report for 5 Years with THSTI Archival Unit.

16. STUDY ORGANIZATION

16.1. Governance

16.1.1. Technical Steering Committee (TSC)

A Technical Steering Committee (TSC) will be established by the funding agency comprising of both national and international experts. A distinguished and renowned scientist will chair the Committee. The members will be from all representative domains such as epidemiology, neonatology, pediatrics and immunology. Additional domain experts will be consulted as and when required.

Specific Roles and Responsibilities:

- review scientific data generated from the study and provide regular feedback to the investigators and the funding agency
- create a measurement matrix for specific outcomes and review if the original goals are being met
- give new directions for research

The committee will meet once or twice a year. Meetings with individual Technical Steering Committee members will take place when and if required by the Chair or the funding agency.

16.1.2. Project Management Team (PMT)

The PMT will comprise of the Principal Investigators from PBC, THSTI, Tribhuvan University, Nepal and CIH, Bergen, Norway and lead investigators from all partnering hospital sites. It is responsible for the overall governance of the study. The PI from THSTI is responsible for coordinating the PMT, which will meet (in person or on skype) every month (or earlier if required). Subject experts will be invited as and when required to give their technical advice. The Clinical Research Coordinator (CRC) from PBC will be the primary link between the study coordination unit (SCU) and the PMT. The SCU will report to the PMT regarding study progress and updates every month. The PMT will review the conduct of the study, which will include visits to the hospital sites, the scientific and other technical aspects.

The final study protocol, management plans, SOPs, logs and the data recording tools with essential site adaptations will be approved by the PMT before the implementation of the protocol.

Specific Roles and Responsibilities:

- overall governance of the study
- to provide a consultative forum that can effectively advice on current and future directions for day to day management of the study.
- to provide advice and support on the planning, implementation, monitoring and reporting of the study
- to review the conduct of the study on the scientific and other technical aspects.

16.1.3. Study Coordination Unit (SCU)

This will comprise of the Clinical Research Coordinator (CRC), Senior Research Officer (SRO), Research Officers (RO)/ Supervisors from each hospital site. The SCU will be responsible for the overall site coordination. It will be responsible for the implementation of the final study protocol. The CRC will be responsible for convening and functioning of the SCU. The SCU will meet (in person or on skype) every 15 days (or earlier if required) to review the management and the progress of the study.

16.2. Study Organization

16.2.1. Responsibility of the site investigators (consultant neonatologists/pediatricians) at the hospital sites

The consultant neonatologists/pediatricians (site investigators) will be responsible for providing all the clinical care to the enrolled participants. They will provide the clinical care to the infants as per standard of care protocol. Regular orientation seminars will be done by the study team for the hospital clinical staff at these hospital sites so that they are aware of the clinical trial being done at their hospital, can identify the infants enrolled in the study from the enrolment ID number and can facilitate the use of the approved protocol for providing standard care to the sick infants.

16.2.2. Study Clinical Team

The study clinical team will comprise of the Clinical Research Coordinator (CRC), Senior Research Officer (SRO), Research Officers (RO), Study Nurses (SN)/ Medical Officers (MO), and Field Technicians (FT)/ Study Assistants. **The study clinical team comprised of Study Nurses/ Medical Officer, and Field Technicians/ Study Assistant will be on duty round the clock and will be available at each hospital site at all times of the day on a rota system.** Broad guidelines of the responsibilities of this team are given below**.**

i. **Clinical Research Coordinator (CRC)**

The Clinical Research Coordinator (CRC) will be leading the study team and will be primary point of contact for operational aspects of implementation of the clinical trial activities from study start-up through database lock, ensuring compliance with GCP and applicable guidance. He/ she will be the primary link between study coordination unit and study investigators. The CRC will have an oversight responsibility for activities undertaken by study coordination unit of the study at all the 4 hospital sites. He/she will be responsible for:

a) Providing input into and/or developing study related materials such as clinical operations plan, SOPS, CRF completion guidelines, taking informed consent, study logs/forms and other study related documents;
b) Supporting the submissions for relevant government / ethics approvals;
c) Developing training module and planning the initial and retraining sessions for the research study staff along with the site supervisors (called research officers) who will be medical doctors;
d) Contribute through operational inputs in protocol and study budget related decisions;
e) Structuring and supervising compliance for the study management plans; Ensuring compliance with the project requirements and cascading the issues/ updates to the relevant stakeholders;
f) Supervising the site preparation, study implementation at site and ongoing study and QC activities;
g) Reviewing protocol deviations and loss to follow up to ensure quality data is delivered;
h) Communicating with site supervisor and site investigator for tracking patient recruitment and progress to study timelines; maintaining and reporting metrics for clinical site performance
i) Providing input and support to maintain appropriate documentation for adverse event safety monitoring, and collaborating in submission of safety reports to sponsor, Ethics Committees and other applicable authorities;
j) Liaisoning with the QM team to ensure good quality of study data;

k) Providing support to site team to prepare for clinical audits and to respond to audit findings conducted by internal QA and external agencies;
l) Supervising the data management progress with data manager and the DM team;
m) Work with coordinating PI to ensure that the trial is meeting its targets, is producing meaningful output and to predict and plan any changes that warrant requests to changes in protocol, funding, or timelines;
n) Keeping stakeholders informed on study progress, risks and accomplishments.

ii. Senior Research Officer (SRO)

The Senior Research Officer will coordinate the study activities at any one of the 4 Delhi hospital sites. He/ she will have a team of 7 study nurses and 4 technicians working under his/her supervision at the site. He/she will be responsible for:

a) Efficient management of clinical operations at the study site- including space for study related activities, responsibility of equipment provided for study activities (calibration, maintenance), ensuring sufficient inventory at site for smooth functioning of study activities.
b) Providing input into study related materials such as clinical operations plan, SOPS, CRF completion guidelines, training module, informed consent, site instructions for specimen collections, study logs/forms and other study related documents;
c) Assisting the Clinical Research Coordinator (CRC) and site investigators for the site preparation;
d) Supervising the study nurses and the clinical technicians posted at the hospital site: including their attendance, punctuality, completion of tasks/ activities assigned to them, training;
e) Supervision of process of taking written informed consent;
f) Supervision of ensuring eligibility of infants before they are enrolled in study;
g) Supervision of assigning the correct randomization code/ intervention to the newly enrolled infant
h) Supervision of outcome assessment
i) Final review of the CRF and the signing of the CRF (as a part of QC);
j) Tracking the reports of all the laboratory samples collected for standard clinical care for ‘clinical severe infection’;
k) Tracking the collection, immediate processing, temporary storage and transportation of bio specimens collected for the research study;
l) Supervising flow of clinical and lab data from point of collection to data management centre;
m) Reviewing participant recruitment, protocol deviations, loss to follow up for hospital site performance;
n) Providing input and support to maintain appropriate documentation for adverse event safety monitoring;
o) Responsible for all logs (calibration, equipment maintenance, training, etc), registers (all enrolments, scheduled follow up registers), documents and site file (updated protocol, informed consent document, eCRF and paper CRFs, all relevant permissions)
p) Responsible for intervention at site-stock, storage at appropriate temperature
q) Liaisoning with the QM team to ensure good quality of study data ;

r) Providing support to CRC and site investigators to prepare for clinical audits and to respond to audit findings conducted by internal QA and external agencies;
s) Liaisoning with QM and DM team and timely resolution of queries in data collected;
t) Keeping site investigators informed on rate of enrolment at site, daily status of all enrolled infants, study progress, risks and accomplishments, any operational problems being faced which are impeding smooth conduct of study

iii. Research Officer

The ROs will supervise the activities of the SN/ MO and the FT/SA and will be responsible for:

a) supervision of process of taking written informed consent
b) supervision of ensuring eligibility of infants before they are enrolled in study
c) supervision of outcome assessment
d) final review of the CRF and the signing of the CRF (as a part of QC)
e) tracking the reports of all the laboratory samples collected for standard clinical care for 'clinical severe infection'
f) tracking the collection, immediate processing, temporary storage and transportation of bio specimens collected for the research study

iv. Study nurses (SN)/ Medical officer (MO):

The SN/ MO will be responsible for:

a) taking written informed consent ;
b) screening ill infants aged 3d-59d who come to the emergency rooms of the hospital, evaluating eligibility criteria and enrolling the infants;
c) assigning the correct randomization code/ intervention to a newly enrolled infant
d) administering the assigned intervention to the enrolled infant as per protocol;
e) collecting data on all the relevant clinical examination for assessing daily progress, outcomes, adverse events
f) completing the case report forms (CRF);
g) collecting biospecimens for the study at the pre-specified time points;
h) maintaining laboratory records in eCRF
i) giving appropriate instructions to the parent(s)/ caregiver of enrolled infant at discharge-completion of 14 day course of assigned intervention, how to administer the intervention, how to document that intervention has been given, when to call the study staff and how to contact the study staff
j) scheduling the follow up visit dates of the enrolled infant at discharge;
k) making reminder calls to the parent (s)/ caregiver of enrolled infant for a scheduled follow up visit
l) collecting clinical data and doing clinical examination at the 15 day follow up visit and clinical data during telephonic follow up visits.
m) In case an enrolled infant has missed a scheduled follow up visit-informing the SRO for corrective action

v. Field technician (FT)/ Study Assistant (SA)

The FT/ SA will be specifically responsible for:

a) assisting the study nurse/ medical officer in administering the intervention to the enrolled infant
b) assisting the SN/MO in collecting bio specimens of the enrolled infant
c) labeling, scanning, immediate processing and temporary storage of collected bio specimens
d) collecting the reports of the routine laboratory tests performed for the enrolled infant from the side lab or designated outsourced laboratory
e) maintaining laboratory records in the eCRF.
f) transportation of all laboratory samples to hospital site side lab/ an accredited laboratory in the close vicinity of the hospital or to the central storage facility at THSTI (India)/ IOM (Nepal) as relevant
g) maintaining detailed input-output charts of the enrolled infants
h) making a home visit to the enrolled infant's house for the post discharge data collection if the need arises
i) making reminder calls to the enrolled infant for scheduled follow up visits in the pediatric OPD
j) maintaining the equipment log, calibration logs, ensuring smooth functioning of equipment at site
k) maintaining the stock inventory at site and reporting to SRO on daily basis on the requirements for the site
l) ensuring cleanliness at site particularly where the lab related study activities will be performed
m) assisting the study nurse/ medical officer in all study related activities at the site
n) assisting the SRO in maintaining all documentation at site-photocoping or scanning of documents if required

17. PROPOSED TIME LINE OF STUDY ACTIVITIES

Figure 2a: Initial proposed Timeline of the study

	2015				2016				2017				2018			
	1	2	3	4	1	2	3	4	1	2	3	4	1	2	3	4
Site Preparation	■	■	■	■	■											
Development of protocol, CRFs, ICD	■	■	■	■	■											
Development of SOPs					■	■										
Development of database						■	■									
Regulatory and Ethics clearances					■	■	■	■								
Recruitment & training of research staff						■	■	■								
Establishing DSMB							■	■								
Pretesting and standardization of procedures across sites							■	■								
Initiation of sites								■	■							
Enrolment of study participants								■	■	■	■	■	■	■		
Laboratory & clinical data collection								■	■	■	■	■	■	■		
Outcome measurement								■	■	■	■	■	■	■	■	
Laboratory assays								■	■	■	■	■	■	■	■	
Data entry								■	■	■	■	■	■	■	■	
Data cleaning															■	■
Data locking															■	■
Data analysis & report writing															■	■

Figure 2b: Revised Timeline of the study

	2016				2017				2019				2020			
	1	2	3	4	1	2	3	4	1	2	3	4	1	2	3	4
Site Preparation	■	■	■	■	■											
Development of protocol, CRFs, ICD	■	■	■	■	■											
Development of SOPs			■	■												
Development of database				■	■											
Regulatory and Ethics clearances			■	■	■	■										
Recruitment & training of research staff				■	■	■										
Establishing DSMB					■	■										
Pretesting and standardization of procedures across sites					■	■										
Initiation of sites						■	■									
Enrolment of study participants							■	■	■	■	■	■	■	■		
Laboratory & clinical data collection							■	■	■	■	■	■	■	■		
Outcome measurement							■	■	■	■	■	■	■	■	■	
Laboratory assays							■	■	■	■	■	■	■	■	■	
Data entry							■	■	■	■	■	■	■	■	■	
Data cleaning														■	■	
Data locking														■	■	
Data analysis & report writing														■	■	

18. CONTRIBUTION OF EACH ORGANIZATION

All the collaborating institutes in the study have agreed to assume responsibilities that are largely non-overlapping, but with regular cross-talk among themselves for implementation of the study design, exchange of information, data and ideas for an efficient conduct of the study.

18.1. THSTI (PBC), SJH, MAMC, CNBC, KH, KSCH

PBC, THSTI will be the main coordinator of the study. The implementation of the study will be coordinated by the Pediatric Biology Centre (PBC) at the Translational Health Science and Technology Institute (THSTI), in the National Capital Region (NCR), Delhi. SJH, MAMC, CNBC, KH and KSCH will be the hospital sites in India for recruitment of eligible infants. During the preparation phase of the study and development of SOPs and other logistical steps, THSTI will work closely with the collaborating institutions and the 7 hospital sites. THSTI will also coordinate the immunological studies to explain potential mechanisms of clinical severe infection and/or role of zinc in clinical severe infection.

CDSA is an extra mural centre of THSTI**.** It will be responsible for the quality management of the study at the Indian hospital sites.

18.2. Tribhuvan University, Institute of Medicine, Nepal

IOM will be the coordinator of the study of the Nepal hospital sites. IOM and KCH will be the hospital sites in Nepal for recruitment of eligible infants. During the preparation phase of the study and development of SOPs and other logistical steps, THSTI will work closely with the IOM and the 7 hospital sites. The laboratory assays for the immunobiological readouts of the samples from the Nepal sites will be analyzed at a central laboratory at IOM.

18.3. Centre for International Health, University of Bergen, Norway (CHN-CIH)

CHN-CIH will play an advisory role and will help in establishing the 'Data Management Centre' for the study at THSTI and in data analysis.

18.4. Centres for Health Policy/Primary Care and Outcomes Research at Stanford University School of Medicine (CHP/PCOR)

CHP/PCOR will collaborate with THSTI to measure the incremental cost effectiveness ratio of zinc supplementation as an intervention for clinical severe infection in infants <2 months.

18.5. Radbound University, Nijmegen, Netherlands

Radbound University will collaborate with THSTI and Institute of Medicine Tribhuvan University for the immunological studies to explain potential mechanisms

19. FINANCING AND BUDGET

This study will be executed as a bilateral collaborative project **under the 'Program of Cooperation' between the Department of Biotechnology**, Indian Institutions (Translational Health Science and Technology Institute, Faridabad; Regional Centre for Biotechnology, Faridabad; Society for Applied Studies, New Delhi; Christian Medical College, Vellore) **and the Norwegian Institutions (University of Bergen, Norway** and Norwegian Institute of Life Sciences, Norway) **under the agreement in Science and**

Technology between Government of Republic of India and Government of Kingdom of Norway (Copy of approval from DBT for executing the study under the Program of Cooperation enclosed as **Annexure III**)

Budget: This study has been granted funding by Research Council of Norway under the Research Grant on Global Health and Vaccination Research (GLOBVAC) (18.02 million Norwegian Kroners) and CISMAC (Centre for Intervention Sciences in Maternal & Child Health), Norway (6.5 million Norwegian Kroners)
The study will be implemented at seven hospital sites, 5 in New Delhi, India and 2 in Kathmandu, Nepal. Initially for easy logistics, it was agreed that funding for the Indian sites and CIH will come from GLOBVAC and funding for the Nepal sites will come from CISMAC. Now, with the closure of one site in Nepal (Patan hospital) and the addition of a site in India (KSCH), funds from CISMAC will be re-appropriated to THSTI for the conduct of study at KSCH. A site wise budget has been prepared for the GLOBVAC and CISMAC funds allocated for the Indian sites. The personnel who will conduct the study at the 4 hospital sites will be employed by Translational Health Science and Technology Institute (THSTI) but deployed at the respective hospitals. There will be no financial transfer of budget to the hospital sites. (Site wise GLOBVAC budget enclosed as **Annexure IV** and CISMAC budget for KSCH enclosed as **Annexure V**)

20. PUBLICATION POLICY

The results of the study will be written up as a report for submission, presentation, or publication. The report will be used for regulatory submissions, presentation at a meeting, or publication in a journal. All publications emanating from this trial will be reviewed by the participating institutions. Authors will be determined based on actual input into the publications, according to existing guidelines.
The report will be authored and approved by the Investigators. Before finalizing it, however, stakeholders will review the report and offer comments. The authors of the study report will be the Investigators conducting the study and the Experts/Advisers to the study.

Following documents are provided as Annexures of the clinical study protocol

- **Annexure I: addendum to protocol with list of Investigators**
- **Annexure II: Circular (File No. 12-01/14-DC (pt. 47)) from the DCGI dated 10-11-2015 and Gazette Notification (Regd. No. D.L.-33004/99) from the Ministry of Health and Family Welfare dated 16-03-2016**
- **Annexure III: Approval from DBT for executing the study under the 'Program of Cooperation'**
- **Annexure IV and V: Site wise budget**
- **Annexure VI: Informed Consent Document (English and Hindi)**
- **Annexure VII: Addendum protocol on extended cost effectiveness analysis [ECEA] for the expanded scope of a secondary objective on Cost-effectiveness analysis**

21. REFERENCES

1. Liu L, Johnson HL, Cousens S, Perin J, et al. Global, regional, and national causes of child mortality: an updated systematic analysis for 2010 with time trends since 2000. Lancet 2012;379(9832):2151-61.
2. Black RE, Cousens S, Johnson HL, et al. Global, regional, and national causes of child mortality in 2008: a systematic analysis. *Lancet* 2010; **375**:1969–87.
3. Lawn JE, Cousens S, Zupan J. 4 million neonatal deaths: when? Where? Why? *Lancet* 2005:**365**;891–900.
4. Young Infants Clinical Signs Study Group. Clinical signs that predict severe illness in children under age 2 months: a multicentre study. *Lancet* 2008; **371**:135–42.
5. The WHO Young Infants Study Group. Bacterial etiology of serious infections in young infants in developing countries: results of a multi-center study. *Pediatr Infect Dis J* 1999; **18**:S17–22.
6. Bassani, DG, Kumar R, Awasthi S. *et al.* Causes of neonatal and child mortality in India: a nationally representative mortality survey. *Lancet* 2010:**376**;1853–1860.
7. Annual Report Department of Health Services (2009-2010) Government of Nepal Accessed on 29th February, 2012 at http://mwrhd.gov.np/annualreports.html
8. Bhatnagar S, Wadhwa N, Aneja S et al. Zinc as adjunct treatment in infants aged between 7 and 120 days with probable serious bacterial infection: a randomised, double-blind, placebo-controlled trial. Lancet 2012; 379:2072-8.
9. Shankar AH, Prasad AS. Zinc and immune function: the biological basis of altered resistance to infection. *Am J Clin Nutr* 1998; **68**:447S–63S
10. Prasad AS. Zinc and immunity. *Mol Cell Biochem* 1998; **188**:63–9.
11. Sazawal S, Jalla S, Mazumder S, Sinha A, Black RE, Bhan MK. Effect of zinc supplementation on cell-mediated immunity and lymphocyte subsets in preschool children. *Indian Pediatr* 1997; **34**:589–97.
12. Bhatnagar S, Bahl R, Sharma PK, Kumar GT, Saxena SK, Bhan MK. Zinc with oral rehydration therapy reduces stool output and duration of diarrhea in hospitalized children: a randomized controlled trial. *J Pediatr Gastroenterol Nutr* 2004; **38**:34–40.
13. Lazzerini M, Ronfani L. Oral zinc for treating diarrhoea in children. *Cochrane Database Syst Rev* 2008; **3**:CD005436.
14. Lukacik M, Thomas RL, Aranda JV. A meta-analysis of the effects of oral zinc in the treatment of acute and persistent diarrhea. *Pediatrics* 2008; **121**:326–36.
15. Bhutta ZA, Black RE, Brown KH, et al. Prevention of diarrhea and pneumonia by zinc supplementation in children in developing countries: pooled analysis of randomized controlled trials. Zinc Investigators' Collaborative Group. *J Pediatr* 1999; **135**:689–97.

16. Sandstead HH. Zinc deficiency. A public health problem? *American Journal of Diseases of Children*, 1991, 145:853–859.

17. Zinc. In: *Trace elements in human nutrition and health*. Geneva, World Health Organization, 1996.

18. Prasad AS et al. Biochemical studies on dwarfism, hypogonadism and anemia. *Archives of Internal Medicine*, 1963, 111:407–428.

19. Brown KH et al. Assessment of the risk of zinc deficiency in populations. *Food and Nutrition Bulletin*, 2004, 25:S130–S162.

20. World health report 2002: reducing risks, promoting healthy life. Geneva, World Health Organization, 2002.

21. Brown KH et al. Overview of zinc nutrition. *Food and Nutrition Bulletin*, 2004, 25:S99–S129.

22. Hotz C, Brown KH. Identifying populations at risk of zinc deficiency: the use of supplementation trials. *Nutrition Reviews*, 2001, 59:80–84.

23. Castillo-Duran C, Vial P, Uauy R. Trace mineral balance during acute diarrhoea in infants. *Journal of Pediatrics*, 1988, 113:452–457.

24. Leonard A, Gerber GB, Leonard F. Mutagenicity, carcinogenicity and teratogenicity of zinc. *Mutation Research*, 1986, 168:343–353.

25. Vallee BL, Falchuk KH. The biochemical basis of zinc physiology. *Physiological Reviews*, 1993, 73:79–118.

26. Samman S. Trace elements. In: Mann J, Truswell S, eds. *Essentials of human nutrition*, 2nd ed. New York, Oxford University Press, 2002.

27. Festa MD et al. Effect of zinc intake on copper excretion and retention in men. *American Journal of Clinical Nutrition*, 1985, 41:285–292.

28. Clinical management of acute diarrhoea: WHO/UNICEF joint statement. Geneva, World Health Organization, 2004.

29. Strand TA et al. Effectiveness and efficacy of zinc for the treatment of acute diarrhoea in young children. *Pediatrics*, 2002, 109:898–903.

30. Bahl R et al. Efficacy of zinc-fortified oral rehydration solution in 6- to 35-month-old children with acute diarrhoea. *Journal of Pediatrics*, 2002, 141:677–682.

31. Penny ME et al. Randomized, community-based trial of the effect of zinc supplementation, with and without other micronutrients, on the duration of persistent childhood diarrhoea in Lima, Peru. *Journal of Pediatrics*, 1999, 135:208–217.

32. Sazawal S et al. Zinc supplementation for four months does not affect plasma copper concentration in infants. *Acta Paediatrica*, 2004, 93:599–602.

33. Bhatnagar S et al. Zinc with oral rehydration therapy reduces stool output and duration of diarrhoea in hospitalized children: a randomized controlled trial. *Journal of Pediatric Gastroenterology and Nutrition*, 2004, 38:34–40.

34. Bhutta ZA, Nizami SQ, Isani Z. Zinc supplementation in malnourished children with persistent diarrhea in Pakistan. *Pediatrics*, 1999, 103:e42.

35. Sazawal S et al. Zinc supplementation in infants born small for gestational age reduces mortality: a prospective, randomized, controlled trial. *Pediatrics*, 2001, 108:1280–1286.

36. Osendarp SJ, West CE, Black RE. The need for maternal zinc supplementation in developing countries. *Journal of Nutrition*, 2003, 133:817S–827S.

37. Sazawal S et al. Effect of zinc supplementation on mortality in children aged 1–48 months: a community-based randomised placebo-controlled trial. *Lancet*, 2007, 369:927–934.

38. Bhandari N et al. The effectiveness of zinc supplementation plus ORS compared to ORS alone as a treatment for acute diarrhoea in a primary health care setting: a cluster randomized trial. *Pediatrics* (in press).

39. Integrated management of childhood illness. World Health Organization, Geneva & UNICEF 2008. Accessed on 29th February, 2012 at http://www.who.int/child_adolescent_health/documents/IMCI_chartbooklet/en/index.html

40. Physicians chart booklet. Integrated Management of Neonatal and Childhood Illness. World Health Organization, Geneva, UNICEF & Ministry of Health & Family Welfare Govt. of India, India: 2003. Accessed on 29th February, 2012 at http://mohfw.nic.in/NRHM/IMNCI/IMNCI_index.htm

41. African Neonatal Sepsis Trial (AFRINEST) group, Tshefu A, Lokangaka A, Ngaima S, Engmann C, Esamai F, Gisore P, Ayede AI, Falade AG, Adejuyigbe EA, Anyabolu CH, Wammanda RD, Ejembi CL, Ogala WN, Gram L, Cousens S. Simplified antibiotic regimens compared with injectable procaine benzylpenicillin plus gentamicin for treatment of neonates and young infants with clinical signs of possible serious bacterial infection when referral is not possible: a randomised, open-label, equivalence trial. *Lancet*. 2015 May 2; 385:1767-76. doi: 10.1016/S0140-6736(14)62284-4.

42. Teresa C. Horan, MPH, Mary Andrus, RN, BA, CIC, and Margaret A. Dudeck, MPH Atlanta, Georgia. CDC/NHSN surveillance definition of health care–associated infection and criteria for specific types of infections in the acute care setting. *Am J Infect Control*. 2008; 36:309-32.

Zinc as an adjunct for the treatment of clinical severe infection in infants younger than 2 months

Version 1.0
Dated: 4-03-2016
Protocol number: RCN/ZINCSEVINF/02/2015

Confidentiality Clause: The confidential information in this document is provided to you as an investigator for review by you, your staff and the applicable Institutional Review Board member. By accepting this document, you agree that the information contained herein will not be disclosed to others, without written authorization from Pediatric Biology Centre, Translational Health Science and Technology Institute, Faridabad

Funding:

Research Council of Norway (RCN) under the Research Grant on Global Health and Vaccination Research (GLOBVAC)
Research Council of Norway
P.O. Box 564
NO-1327 Lysaker, Norway

CISMAC (Centre for Intervention Sciences in Maternal & Child Health), Norway
Anchored at the Centre for International Health (CIH), University of Bergen, Norway

Partnering Institutes:

Pediatric Biology Centre, Translational Health Science and Technology Institute, Faridabad

Department of Child Health, Institute of Medicine, Tribhuvan University, Kathmandu, Nepal

Centre for International Health, Bergen, Norway
Innlandet Hospital Trust, Norway

Stanford University School of Medicine, USA

Radbound University, Nijmegen, The Netherlands

Coordinating Centre:

Pediatric Biology Centre, Translational Health Science and Technology Institute, Faridabad

Study Hospital Sites **<u>Delhi, India</u>**

- Maulana Azad Medical College (MAMC) and associated Lok Nayak Hospital
- Vardhman Mahavir Medical College & Safdarjung Hospital (VMMC & SJH)
- Chacha Nehru Bal Chikitsalaya (CNBC) and
- Kasturba Hospital (KH)

<u>Nepal</u>

- Patan Hospital [PH], Kathmandu,
- Kanti Children's Hospital (KCH), Kathmandu and
- Institute of Medicine (IOM), Kathmandu.

Investigators:

New Delhi, India

Dr. Nitya Wadhwa
Principal Investigator
Pediatric Biology Centre,
Translational Health Science and Technology Institute,
Faridabad-121001
Tel No: +91 129 2876342/ +91 9811142248
Email: nitya.wadhwa@thsti.res.in

Dr. Shinjini Bhatnagar
Co-Principal Investigator
Pediatric Biology Centre,
Translational Health Science and Technology Institute,
Faridabad-121001
Tel No: +91 129 2876351/ +91 129 2876362
Email: shinjini.bhatnagar@thsti.res.in

Dr. KC Aggarwal
Site Investigator
Department of Pediatrics,
VMMC & Safdarjung Hospital,
New Delhi-110029
Tel No: +91 11 26707241/ +91 11 26198106
Email: kcagg1955@rediffmail.com

Dr. Sunita Bhatia
Site Investigator
Department of Pediatrics,
Kasturba Hospital,
Delhi-110002
Tel No: +91 9811767569
Email: sunitabhatia54@gmail.com

Dr. Meenakshi Bhatt
Lead Site Investigator
Department of Pediatrics,
VMMC & Safdarjung Hospital,
New Delhi-110029
Tel No: +91 9873659404
Email: meenakshi491@hotmail.com

Dr. Harish Chellani
Site Investigator
Department of Pediatrics,
VMMC & Safdarjung Hospital,
New Delhi-110029
Tel No: +91 11 26181862
Email: chellaniharish@gmail.com

Dr. Aashima Dabas
Site Investigator
Department of Pediatrics,
Chacha Nehru Bal Chikitsalaya,
Delhi-110031
Tel No: +91 9868479647
Email: dr.aashimagupta@gmail.com

Dr. Anuradha Govil
Lead Site Investigator
Department of Pediatrics,
Kasturba Hospital,
Delhi-110002
Tel No: +91 11 27569042/ +91 9811220768
Email: dranuradhagovil@gmail.com

Dr. Mamta Jajoo
Site Investigator
Department of Pediatrics,
Chacha Nehru Bal Chikitsalaya,
Delhi-110031
Tel No: +91 9643308217
Email: mamtajajoo123@gmail.com

Dr. Ajay Kumar
Site Investigator
Department of Neonatology,
Maulana Azad Medical College and associated Lok Nayak Hospital
New Delhi-110002
Tel No: +91 9968604310
Email: ajayneonatology@gmail.com

Dr. NB Mathur
Lead Site Investigator
Department of Neonatology,
Maulana Azad Medical College and associated Lok Nayak Hospital
New Delhi-110002
Tel No: +91 9968604308
Email: drnbmathur@gmail.com

Dr. Anup Mohta
Lead Site Investigator
Department of Pediatric Surgery,

Chacha Nehru Bal Chikitsalaya,
Delhi-110031
Email: directorcnbc@gmail.com

Dr. Siddarth Ramji
Site Investigator
Department of Neonatology,
Maulana Azad Medical College and
associated Lok Nayak Hospital
New Delhi-110002
Tel No: +91 11 23236031
Email: siddarthramji@gmail.com

Dr. Uma Chandra Mouli Natchu
Co-Investigator
Pediatric Biology Centre,
Translational Health Science and Technology Institute,
Faridabad-121001
Tel No: +91 129 2876356
Email: unatchu@thsti.res.in

Dr. Shailaja Sopory
Co-Investigator
Pediatric Biology Centre,
Translational Health Science and Technology Institute,
Faridabad-121001
Tel No: +91 129 2876345
Email: ssopory@thsti.res.in

Dr. Guruprasad Medigeshi
Co-Investigator
Vaccine and Infectious Disease Research Centre
Translational Health Science and Technology Institute,
Faridabad-121001
Tel No: +91 129 2876311
Email: gmedigeshi@thsti.res.in

Nepal

Dr. Sudha Basnet
Co-Principal Investigator
Department of Child Health,
Institute of Medicine, Tribhuvan University,
Kathmandu, Nepal
G.P.O. Box No 2533
Postal Code 1
Kathmandu, Nepal
Tel No: +977 14412202/ +977 9841209799
Email: sudhacbasnet@gmail.com

Dr. Srijana Basnet
Site Investigator
Department of Child Health,
Institute of Medicine, Tribhuvan University,
Kathmandu, Nepal
Email: drsrijanabasnet@yahoo.com

Dr. Imran Ansari
Lead Site Investigator
Department of Pediatrics,
Patan Hospital,
Patan Academy of Health Sciences (PAHS)
Lalitpur, Nepal
Email: imranansari@pahs.edu.np

Dr. Ganesh Prasad Shah
Site Investigator
Patan Academy of Health Sciences
Patan Hospital,
Department of Pediatrics,
Lalitpur, Nepal
Email: ganeshshah59@yahoo.com

Dr. Binod Man Shreshtha
Lead Site Investigator
Medical Department,
Kanti Children's Hospital,
Kathmandu, Nepal
Email: drbinod@yahoo.com

Dr. Laxman Prasad Shrestha
Lead Site Investigator
Department of Child Health,
Institute of Medicine, Tribhuvan University,
Kathmandu, Nepal
Tel No: +977 14412202 / +977 985036550
Email: laxmanshree12@gmail.com

Dr. Ram Hari Chapagain
Site Investigator
Medical Department,
Kanti Children's Hospital,
Kathmandu, Nepal
Email: chapagainrh2007@gmail.com

Centre for International Health, Bergen

Dr. Tor A. Strand
Co-Principal Investigator
Centre for International Health (CIH),
University of Bergen (UiB),
PB 7804, N-5009 Bergen, Norway
Tel No: +47 5558 8575/ +47 4064 0882
Email: tor.strand@cih.uib.no

Dr. Halvor Sommerfelt
Co-Investigator
Centre for International Health (CIH),
University of Bergen (UiB),
PB 7804, N-5009 Bergen, Norway
Tel No: +47 5558 8575/ +47 4064 0882
Email: halvor.sommerfelt@cih.uib.no

Stanford University School of Medicine, USA

Dr. Jeremy Goldhaber-Fiebert
Co-Investigator
Centers for Health Policy/
Primary Care and Outcomes Research
Stanford University School of Medicine
Stanford, USA
Email: jeremygf@gmail.com

Radbound University, The Netherlands

Dr. Mihai Netea
Co-Investigator
Radbound University, Nijmegen
The Netherlands
Email: Mihai.Netea@radboundumc.nl

Table of Contents

GLOSSARY ... 8
1. TRIAL SYNOPSIS ... 10
2. BACKGROUND INFORMATION ... 12
2.1 Name and Description of the Investigational Product ... 12
2.2 Introduction ... 12
2.3 Relevant previous human studies ... 12
2.4 Rationale ... 12
2.5 Summary of known potential Risks and Benefits ... 13
2.6 Justification for Route of Administration, Dosage, Dose Regimen and Treatment Period ... 17
2.7 Rationale for Study Design ... 18
2.8 Description of Population ... 18
3. STUDY OBJECTIVES ... 19
3.1 Hypothesis ... 19
3.2 Objectives ... 19
3.3 Outcomes ... 20
4. TRIAL DESIGN ... 22
5. Randomization ... 24
5.1. Stratification: ... 24
5.2. Preparation and safe keeping of the randomization lists: ... 24
5.3. Allocation concealment: ... 24
5.4. Masking: ... 25
5.5. Study Procedures ... 25
5.6. Standard Case Management ... 33
5.7. Laboratory procedures ... 33
6. Supplies and Handling of Materials ... 37
6.2. Storage ... 38
6.3. Clinical Supplies Accountability ... 38
6.4. Source Documents ... 38
7. WITHDRAWAL CRITERIA ... 38
8. SAFETY MONITORING ... 39
8.1. Adverse Events ... 39
8.2. Relatedness to Intervention ... 40
8.3. Specification of Safety Parameters ... 41

8.4. Data Safety Monitoring Board 41
8.5. Independent study monitoring 42
8.6. Adverse event reporting 42
8.7. Follow Up After Adverse Events 43
8.8. Trial Termination 43
9. Ethical Issues and Approvals 43
9.1. Ethical issues: 43
9.2. Ethical Approvals 43
10. STATISTICS 43
10.1. Sample Size 43
10.2. Analysis 44
11. DIRECT ACCESS TO SOURCE DATA/ DOCUMENTS 49
12. QUALITY CONTROL AND QUALITY ASSURANCE 49
12.1. Staff Training 50
12.2. Site Monitoring 50
12.3. Auditing 51
13. ETHICS COMMITTEE CLEARANCES 52
14. CONSENT FOR PARTICIPATION 52
15. DATA HANDLING AND RECORD KEEPING 54
15.1. Data Management 54
15.2. Record Retention and Archival 55
16. STUDY ORGANIZATION 55
16.1. Governance 55
16.2. Study Organization 56
17. PROPOSED TIME LINE OF STUDY ACTIVITIES 59
18. CONTRIBUTION OF EACH ORGANIZATION 60
18.1. THSTI (PBC), SJH, MAMC, CNBC, KH 60
18.2. Tribhuvan University, Institute of Medicine, Nepal 60
18.3. Centre for International Health, University of Bergen, Norway (CHN-CIH) 60
18.4. Centres for Health Policy/Primary Care and Outcomes Research at Stanford University School of Medicine (CHP/PCOR) 60
18.5. Radbound University, Nijmegen, Netherlands 60
19. FINANCING AND BUDGET 60
20. PUBLICATION POLICY 61
21. REFERENCES 62

GLOSSARY

ANC: Absolute Neutrophil Count

CHP/PCOR: Centres for Health Policy/Primary Care and Outcomes Research

CIH: Centre for International Health

CISMAC: Centre for Intervention Sciences in Maternal & Child Health

CNBC: Chacha Nehru Bal Chikitsalaya

CRC: Clinical Research Coordinator

CRF: Case Report Form

DSMB: Data Safety Monitoring Board

EC: Ethics Committee

FT: Field Technician

GCP: Good Clinical Practice

GLOBVAC: Global Health and Vaccination Research

IEC: Institutional Ethics Committee

IOM: Institute of Medicine

IRB: Institutional Review Board

KCH: Kanti Children's Hospital

KH: Kasturba Hospital

MAMC: Maulana Azad Medical College

MO: Medical Officer

PAHS: Patan Academy of Health Sciences

PBC: Pediatric Biology Centre

PH: Patan Hospital

PI: Principal Investigator

PMT: Project Management Team

RCN: Research Council of Norway

RO: Research Officer

SA: Study Assistant

SAE: Serious Adverse Event

SCT: Site Coordination Team

SJH: Safdarjung Hospital

SN: Study nurse

SOP: Standard Operating Procedure

SRO: Senior Research Officer

THSTI: Translational Health Science and Technology Institute

TLC: Total Leukocyte Count

UiB: University of Bergen

VMMC: Vardhman Mahavir Medical College

1. TRIAL SYNOPSIS

Study Title: Zinc as an adjunct for the treatment of clinical severe infection in infants younger than 2 months

Sponsor: Pediatric Biology Centre, Translational Health Science and Technology Institute

Funding Agency: Research Council of Norway (RCN) under the Research Grant on Global Health and Vaccination research (GLOBVAC) and CISMAC (Centre for Intervention Sciences in Maternal & Child Health, Norway

Study Intervention: 10mg of elemental zinc per day given as oral zinc sulphate drops Vs placebo drops

Study design: Double-blind randomized placebo-controlled parallel group superiority trial.

Duration of study intervention: 10 mg elemental zinc given as 5 mg oral drops twice a day 12 hours apart for a total of 14 days

Objectives of study:

Primary objectives:

i. To estimate the efficacy of 10 mg elemental zinc administered orally as an adjunct to standard antibiotic therapy to infants aged 1 day up to 2 months (59 days) hospitalized with 'clinical severe infection' against **case fatality**.
ii. **Time to death until end of study period** defined as the time from enrolment to the time when an infant dies anytime until 12 weeks from the day of enrolment in the study.

Secondary objectives: Secondary objectives are to estimate the efficacy of 10 mg elemental zinc administered orally as an adjunct to standard antibiotic therapy to infants aged 1 day up to 2 months (59 days) hospitalized with 'clinical severe infection' against the following **outcomes**:

i. **Failure of treatment**
ii. **Time to cessation of symptoms and signs of clinical severe infection**
iii. **Time to failure of treatment**
iv. **Time to discharge**
v. **Time to death**
vi. **Death at any time after discharge from hospital until end of study period which is 12 weeks from the day of enrolment.**
vii. **Severe illness requiring hospitalisation at any time after discharge from hospital until end of study period which is 12 weeks from the day of enrolment.**

viii. **Cost-effectiveness analysis**

ix. **To undertake mechanistic studies of immune system**

Study Procedures: The trial will be conducted over a period of 3 years and 6 months. This is a multicenter study where recruitments will take place in 7 centres, 4 in New Delhi India and 3 in Kathmandu, Nepal. The trial will measure the efficacy of zinc administered orally as an adjunct to standard therapy to infants aged 1 day to 2 months hospitalized with clinical severe infection identified using an adaptation of the WHO Integrated Management of Childhood Illnesses (IMCI) criteria. The participants will be randomized to receive zinc or placebo in a 1:1 allocation ratio. The intervention (zinc/ placebo drops) will be co-administered with the standard therapy which includes intravenous antibiotics and other supportive therapy like intravenous fluids, supplemental oxygen, etc. daily at 12 hrly intervals from the time of enrolment for 14 days. 4140 infants with clinical severe infection will be enrolled, given intervention for 14 days and followed up till discharge and until 12 weeks from the day of enrolment.

Biospecimen collection:
Blood sampling for the study:

i. At enrolment: 3 ml of venous blood will be collected at admission (enrolment) from the enrolled infants with clinical severe infection for study related investigations
ii. At 48-72 hours: 2-3 ml of blood will be collected
iii. At discharge: 2-3 ml of blood will be collected

Nasal swab for viral etiology at enrolment

2. BACKGROUND INFORMATION

2.1 Name and Description of the Investigational Product

Elemental zinc given as zinc sulphate drops (Z&D)

2.2 Introduction

More than 3 of the 7.6 million global deaths in children under 5 years of age occur in the neonatal period[1] and about 75% in the first week of life.[2] More than 70% of the neonatal deaths occur in Africa and South East Asia.[1] Severe infections like pneumonia and sepsis contribute to 25% of these deaths [3] and are also a major cause of hospitalization in infants.[4] Despite appropriate antimicrobial therapy, the outcome of these severe infections in early infancy is poor.[5] Effective interventions that can be added to standard therapy for severe infections are required to improve clinical outcomes and to reduce case fatality.

Serious infections like pneumonia, sepsis, and meningitis contribute to more than 25% of the annual 1 million neonatal deaths in India.[6] Also in Nepal, sepsis is a leading cause of death in neonates, and the second most frequent reason for hospitalization.[7] While appropriate antibiotics are available in many hospitals in India and Nepal, second-line antibiotics may be unavailable or are prohibitively expensive in peripheral health facilities. It is important to develop inexpensive, effective and accessible interventions that can be added to standard therapy for severe infections to improve treatment outcomes and reduce case fatality.

2.3 Relevant previous human studies

In a recent randomized placebo-controlled trial conducted in 3 tertiary hospitals in New Delhi, it was found that 10 mg of elemental zinc given daily to 7 to 120 days old infants treated with antibiotics for probable serious bacterial infection (PSBI) carried a 40% (95% CI 10% to 60%) efficacy against treatment failure.[8] The absolute risk reduction was 6.8% (95% CI 1.5% to 12.0%), indicating that 15 (95% CI 8 to 67) infants would need to be treated with zinc in addition to antibiotics to prevent one treatment failure. This study of 700 infants is, to our knowledge, the first report of the efficacy of zinc in the treatment of PSBI in infants younger than 4 months. An even larger efficacy against treatment failure (54% [95% CI 20% to 74%; P=0.005)] was seen when analysis was restricted to 1 week to 2 month old infants, among whom only 11 (95% CI 6 to 37) would need to receive adjunct zinc treatment to prevent one treatment failure. The point estimate for the efficacy of adjunct zinc therapy against death (43%) was the same as that against treatment failure, albeit with poorer precision (95%CI -23% to 73). However, the study was not powered to estimate the effect of zinc on case fatality.

Our main hypothesis that zinc will reduce the risk of treatment failure in infants with clinical severe infection was based on publications showing that zinc is crucial for immune function [9-11] and the benefit of oral zinc in infectious diseases like acute childhood diarrhoea.[12-15]

2.4 Rationale

More than 3 of the 7.6 million global deaths in children under 5 years of age occur in the neonatal period[1] and about 75% in the first week of life.[2] More than 70% of the

neonatal deaths occur in Africa and South East Asia.[1] Severe infections like pneumonia and sepsis contribute to 25% of these deaths [3] and are also a major cause of hospitalization in infants.[4] Despite appropriate antimicrobial therapy, the outcome of these severe infections in early infancy is poor.[5] Effective interventions that can be added to standard therapy for severe infections are required to improve clinical outcomes and to reduce case fatality.

Based on the promising results of the above-mentioned trial, [8] a large, multicentre study powered to examine the effect of zinc on case fatality from clinical severe infection would contribute evidence towards revising treatment recommendations for low resource settings in South Asia and elsewhere.

2.5 Summary of known potential Risks and Benefits

Risks

Zinc is a micronutrient that can be found in all tissues of the body and is essential for cell growth, cell differentiation and DNA synthesis.[16] It is also essential for the maintenance of a healthy immune system.[17] Human zinc deficiency was first identified by Prasad et al. in the 1960s in growth-retarded adolescent boys in Egypt .[18] A population level analyses from food balance sheets have estimated that 21% of the world's population is at risk of zinc deficiency.[19] Children in many developing countries around the world typically consume fewer animal products than adults, especially in the developing world, which results in both stunting and zinc deficiency. A high rate of stunting is considered indicative of zinc deficiency among children less than 5 years of age.[19] WHO has identified zinc deficiency as a major risk to child health, and has linked it to morbidity from diarrhoea, lower respiratory tract infections and malaria, accounting for 0.8 million-child deaths per year.[20]

The International Zinc Consultative Group (IZiNCG) revised the recommended dietary allowances (RDAs) in 2004.[21] These recommendations suggest the following:

Group	RDA of zinc
Infants	4–5 mg
Children 1–3 years of age	3 mg
Children 4–8 years of age	4–5 mg
Non-pregnant women	8–9 mg
Pregnant and lactating women	9–13 mg
Men	13–19 mg

These recommendations take into account differences in diet and are based on a standard reference body weight. Children receiving diets higher in phytate, which is found in unrefined cereals, will need to consume more zinc each day to achieve the physiological requirement. In addition, these guidelines are for healthy children and

do not take into account the excess zinc losses during an episode of diarrhoea[22, 23] or the extra zinc required for catch-up growth and development.

In extensive safety studies undertaken in laboratory animals, zinc has been shown *not* to be carcinogenic, mutagenic or teratogenic .[24] In addition, the human body has efficient homeostatic mechanisms that regulate the absorption and retention of zinc, and these reduce the likelihood of toxic build-up in the body .[25] Zinc toxicity in adults can occur following moderately high intakes of zinc (>150 mg/day or approximately 10 times the RDA) over a long period of time or from ingestion of >1 g of zinc (more than 60 times the RDA) by overdose via supplementation or intravenous feeding .[26] Ingesting too much zinc at once can cause gastric distress and the typical signs and symptoms often associated with food poisoning.

High doses of zinc for long periods may lead to a lower concentration of plasma lipoproteins and decreased copper absorption.[26] A lower copper status may also inhibit the transport of iron and result in anaemia.[27] Although zinc-induced copper deficiency and the resulting anaemia are serious, they occur only after excessive zinc intake over a long period and are easily corrected by adjusting the intake of zinc and copper accordingly.[25] Supplements of zinc and iron may also compete for absorption in the body.

Cases have been reported of acute, though reversible, adverse reactions from inhaling zinc vapours – a condition known as “zinc fume fever” – and from ingesting food or drink stored in galvanized containers.[25] There are also a number of reported cases of adverse effects due to excessive zinc intake. The majority of these cases involved adults who knowingly ingested many times the normal daily dose of zinc over a long period. Even in the most extreme cases (more than 1 g/day for many months), patients recovered from all signs and symptoms, including fatigue, gastrointestinal discomfort and anaemia, as soon as zinc intake was decreased and serum zinc returned to the normal range.

In the report of the WHO Collaborating Centre for International Drug Monitoring in Uppsala, Sweden, there were 50 cases of adverse effects of oral zinc sulfate, including 56 clinical signs and symptoms. The majority of these cases involved the patient taking or being given several drugs at the same time, so it was not always possible to identify the cause of the observed sign(s) and/or symptom(s). The cases documented in the report all varied with respect to dosage, patient age, certainty of causality of association and number of additional drugs. **There were only 2 reported cases in which the likelihood of causation by zinc was “certain”, and only 1 in a child (side-effect: epistaxis) There were 20 reported cases where the likelihood was “possible” and 9 where it was “probable.”** The level of causation could not be established in the other reported cases. There were four reports of possible adverse responses to zinc ingestion among children less than 10 years of age.

Short-course supplementation trials

After more than 20 years of extensive research, zinc supplementation for the treatment of diarrhoea is now recommended by WHO and UNICEF .[28] Current recommendations are for 10–14 days of supplementation for all episodes of diarrhoea among children less than 5 years of age. Infants less than 6 months of age are to receive 10 mg daily and children aged between 6 months and 5 years are to

receive 20 mg daily. These doses have been proven to be both effective and safe for treatment during diarrhoea.

To date there have been no reports of severe adverse reactions from any form of zinc treatment for diarrhoea. Trials have included more than 9100 children who have participated in efficacy trials in both the placebo and zinc study arms, and nearly 12 000 child-years of observation from one large effectiveness trial. **The zinc doses ranged from 5 to 45 mg/day and were well-tolerated in diverse settings. No differences in adverse reactions were found based on the different zinc salts used in supplementation trials, i.e. the sulfate, acetate and gluconate.**

At present, the only reported side-effect of zinc treatment has been vomiting. Of the seven trials that have reported incidences of vomiting, only two reported more vomiting in the zinc-treated children compared with those given a placebo.[29, 30] One trial reported more vomiting than in control children when zinc was given with multiple micronutrients but not when given alone.[31]

Copper status has been evaluated in four trials. In three of these, no difference in serum copper status was found after supplementation.[29, 32,33] In the fourth trial, a significant trend towards lower copper status was found in zinc-treated compared to untreated children[34]; however, these children were malnourished with persistent diarrhoea at baseline. Overall, there is no substantial evidence that short-term zinc administration for the treatment of diarrhoea adversely affects copper status.

In addition to trials on the treatment of diarrhoea, there have been several trials assessing the efficacy of zinc for the treatment of pneumonia, malaria, measles and the common cold. Treatments have typically included approximately 20 mg/day for the duration of the illness, which is typically less than two weeks. No serious adverse effects linked to zinc have been reported in these studies.

In a **recent clinical trial in India** where 700 infants aged 7d -120d were given either 10 mg elemental zinc or placebo, no serious adverse events were demonstrated to be associated with oral zinc supplementation when given in a dose of 10mg elemental zinc per day till recovery and hospital discharge. The risk of adverse events that were not components of the clinical outcomes was similar in the two trial arms

**Adverse events***	**Zinc n=352**	**Placebo n=348**	**RR (95% CI)**
Vomiting related to the time of intervention:			
Vomited the first dose of intervention within 30 minutes	3 (0·85)	3 (0·86)	0·99 (0·20, 4·86)
Vomited any subsequent intervention within 30 minutes	6 (1·70)	6 (1·72)	0·99 (0·32, 3·04)
Vomited once or more times during the study period unrelated to time of intervention	41 (11·65)	36 (10·34)	1·13 (0·74, 1·72)
Abdominal distension lasting ≥ 24 hours at any	13	10 (2·87)	1·28 (0·57,

time during the study period	(3·69)	2·89)

Long-term supplementation trials

A number of long-term supplementation trials have been carried out among young children and pregnant women. Children have been given zinc supplements to improve growth and to prevent pneumonia, diarrhoea and malaria. In 1999, a review of seven zinc supplementation studies for the prevention of diarrhoea and pneumonia was published .[15] These trials were conducted in a variety of study populations with different baseline nutritional status. Supplementation with zinc ranged from 5 to 20 mg/day for up to a year. No adverse effects were reported in these studies. There have been two studies in which children were given zinc supplements for the prevention of malaria. Children were given supplements of up to 70 mg zinc, twice a week for up to 15 months, and no adverse effects were reported.

One supplementation trial of low-birth-weight infants demonstrated not only the safety of zinc but the benefits of daily supplementation among these vulnerable babies .[35] A total of 581 Indian infants aged 30–284 days were each given 5 mg zinc. Those who received the zinc supplementation had a two-thirds lower risk of dying over the study period. There were 5 deaths among infants receiving zinc and 15 deaths among infants who did not receive zinc. There were no adverse events linked to zinc supplementation in this trial.

There have been several trials of zinc supplementation among pregnant women .[36] Because the demands of zinc increase during pregnancy, zinc supplementation may provide benefits to the pregnant mother and the growing fetus, especially in countries where zinc intake is lower than the recommended standards. Although the benefits of providing pregnant women with zinc supplements have yet to be confirmed, there are no published reports of adverse effects during these trials in either the pregnant women or their infants.

Further studies on **zinc supplementation** have been completed in the past two years, involving thousands of children who received either **10 mg elemental zinc per day for up to two years**[37] or 40 mg zinc for up to 14 days for the management of acute diarrhoea .[38] Except for mild regurgitation in a few studies, no serious adverse effects of oral zinc were seen in any of the studies.

Precautions

As with any treatment, zinc supplements should be kept in a safe place to prevent the accidental ingestion of more than the recommended dose. In the unlikely event that a child consumes several daily doses of the supplements, he/she would probably vomit quickly. There is no evidence to suggest that further adverse events would occur but, as in the case of any accidental ingestion of medication, the child should be taken to a health care provider.

Conclusion

Zinc supplementation is a safe and effective treatment for diarrhoea. Zinc has also been shown to be safe in long-term supplementation studies. The most severe adverse effects noted in supplementation trials have been vomiting in some cases and a slight reduction in copper status in some children. Neither has been shown to cause any long-term harm. Although there have been case reports in adults of excessive zinc intake, the adverse effects even in these cases have been limited to short-term morbidity, and few have resulted in any long-term sequelae.

Potential benefits to enrolled participants in the current clinical trial could be reduced risk of case fatality, need for changing antibiotics, prolonged hospital stay and use of high-generation antimicrobials; education on personal hygiene and access to medical care for the participant for any illness occurring during the study period.

2.6 Justification for Route of Administration, Dosage, Dose Regimen and Treatment Period

2.6.1 **Route of Administration:** The ease of administration by oral route makes this route a preferred choice. If zinc is proven to be effective in reducing case fatality in infants with clinical severe infection, care providers at small health-care facilities can be trained to initiate treatment of clinical severe infection with antibiotics (as recommended by IMNCI) and oral zinc before transferring infants to appropriately equipped facilities.

2.6.2 **Dosage:** In a recent randomized placebo-controlled trial conducted in 3 tertiary hospitals in New Delhi, it was found that 10 mg of elemental zinc given daily to 7 to 120 days old infants treated with antibiotics for probable serious bacterial infection (PSBI) carried a 40% (95% CI 10% to 60%) efficacy against treatment failure. In this study about 43%-enrolled infants were found to have low serum zinc at enrolment. After supplementation there was a 39% increase in serum zinc levels at discharge suggesting that this dose is adequate.

In addition to being efficacious, no serious adverse events were demonstrated to be associated with oral zinc supplementation when given in a dose of 10mg elemental zinc per day till recovery and hospital discharge.

2.6.3 **Dose Regimen:** In the above-mentioned placebo-controlled trial conducted in New Delhi, oral zinc was given in the dose of 10mg elemental zinc divided in two doses given 12 hours apart.

We will replicate the same dose regimen of 5mg of elemental zinc given 12 hourly in our current study

2.6.4 **Treatment Period:** Once enrolled in the study oral zinc will be given in the dose of 10mg elemental zinc for a total of 14 days. This is based on previous studies where beneficial effects of zinc beyond the supplementation period have been seen when it was given for 14 days.

2.7 Rationale for Study Design

The study has been designed as a placebo-controlled randomized trial similar to the design of our previous study on the efficacy of zinc used as an adjunct to standard antibiotic therapy in reducing treatment failure in young infants with probable serious bacterial infection. The study has similar safeguards for standard medical care in place to minimize risk of adverse events for the participants.

If the results of this study are consistent with our earlier trial, this would substantially strengthen the evidence for recommending zinc as an adjunct to standard therapy for clinical severe infection in young infants. Further, as we propose to identify young infants with clinical severe infection using an adaptation of IMNCI criteria, it would be easier to justify introduction of zinc in the national programs where IMNCI is followed.

2.8 Description of Population

The patient recruitment will be done in 7 centres: 4 secondary level hospitals in Delhi, India (Maulana Azad Medical College (MAMC) and associated Lok Nayak Hospital, Vardhman Mahavir Medical College & Safdarjung Hospital (VMMC & SJH), Chacha Nehru Bal Chikitsalaya (CNBC) and Kasturba Hospital (KH)), and 3 centres in Nepal (Patan Hospital [PH], Kathmandu, Kanti Children's Hospital (KCH), Kathmandu and Institute of Medicine (IOM), Kathmandu), The study will be coordinated by PBC at THSTI.

India centres: The selected hospitals in North India are secondary referral level hospitals that are responsible for a defined geographical area. Their pediatric department bed strength is around 150-250 beds depending on the size, terrain and population of the area covered by the hospital. Secondary level hospitals provide all basic specialty services. Almost 25% of pediatric admissions at these hospitals are for infant sepsis.

Nepal centres: PH, under Patan Academy of Health Sciences, Kathmandu, has a 75-bedded Pediatric ward, with a separate neonatal nursery and both pediatric and neonatal intensive care units. According to an audit of hospital admissions over a period of 6 months, infants < 2 months accounted for 35% of total pediatric admissions with sepsis in 71% as the most common diagnosis (verbal communication). KCH has a capacity of 300 beds. About 50% of the referrals are from outside the Kathmandu valley. Neonatal sepsis accounts for 50% of the total admissions in the neonatal intermediate care unit of this hospital. IOM, with a 60-bedded pediatric ward, recently opened a neonatal nursery and separate neonatal and pediatric intensive care units and admits some 300 patients each month, of whom almost 50 have sepsis.

The selected clinical sites are secondary level hospitals with similar standard of care. This will make it easier to standardize study protocol across the hospitals. Each of the seven hospitals has a large patient load particularly of young infants with clinical severe infection.

The above hospitals have been selected because they get a large number of young infants with clinical severe infection and are therefore most relevant for this intervention.

3. STUDY OBJECTIVES

3.1 Hypothesis

Our hypothesis is that daily administration of 10 mg elemental zinc orally as adjunct to standard therapy to infants aged 1 day up to 2 months (59 days) hospitalized with clinical severe infection will lead to a relative case fatality risk reduction of at least 30%.

3.2 Objectives

3.2.1 Primary objectives:

i. To estimate the efficacy of 10 mg elemental zinc administered orally as an adjunct to standard antibiotic therapy to infants aged 1 day up to 2 months (59 days) hospitalized with 'clinical severe infection' against **case fatality**.
   The case fatality risk is the proportion of children with 'clinical severe infection' who die due to any cause and at any time from enrolment until hospital discharge.

ii. **Time to death until end of study period** defined as the time from enrolment to the time when an infant dies anytime until 12 weeks from the day of enrolment in the study.

3.2.2 Secondary objectives:

Secondary objectives are to estimate the efficacy of 10 mg elemental zinc administered orally as an adjunct to standard antibiotic therapy to infants aged 1 day up to 2 months (59 days) hospitalized with 'clinical severe infection' against the following <u>**outcomes**</u>:

i. **Failure of treatment**. There is failure of treatment if one or more of the following events occur:

   a) Death at any time after enrolment until hospital discharge, i.e. as primary objective i., or

   b) Initiation of life support at any time after enrolment until hospital discharge. Need for life support will be defined as a need for ventilation or vasoactive drugs at any time after enrolment until hospital discharge, and/or

   c) A change in antibiotics for one of the following circumstances:

      i) Persistence of signs that indicate 'clinical severe infection' present at the time of enrolment anytime after 48 hours of enrolment and prior to discharge.

      ii) Worsening of existing signs or appearance of new signs of 'clinical severe infection' any time after enrolment and prior to discharge

      iii) Reappearance of signs of 'clinical severe infection' that the infant presented with at time of enrolment anytime after 48 hours of disappearance and prior to discharge.

ii. **Time to cessation of symptoms and signs of clinical severe infection**: This will be defined as the time from enrolment to the beginning of a 48 hour period with none of the signs of ‘clinical severe infection’.

iii. **Time to failure of treatment**: This will be defined as the time from enrolment to the time when an infant is deemed to have failed treatment as per the definition of **secondary objective i**.

iv. **Time to discharge**: The time from enrolment to discharge from the hospital.

v. **Time to death**: This will be defined as the time from enrolment to the time when an infant dies while admitted in the hospital for ‘clinical severe infection’.

vi. **Death at any time after discharge from hospital until end of study period which is 12 weeks from the day of enrolment.**

vii. **Severe illness requiring hospitalisation at any time after discharge from hospital until end of study period which is 12 weeks from the day of enrolment.**

viii. **Cost-effectiveness analysis:** To measure the incremental cost effectiveness ratio of zinc supplementation as an intervention for ‘clinical severe infection’ in infants < 2 months.

ix. **To undertake mechanistic studies of immune system**

3.3 Outcomes

3.3.1 Primary Outcomes

i. To estimate the efficacy of 10 mg elemental zinc administered orally as an adjunct to standard antibiotic therapy to infants aged 1 day up to 2 months (59 days) hospitalized with ‘clinical severe infection’ against **case fatality**.
The case fatality risk is the proportion of children with ‘clinical severe infection’ who die due to any cause and at any time from enrolment until hospital discharge.

ii. **Time to death until end of study period** defined as the time from enrolment to the time when an infant dies until 12 weeks from the day of enrolment in the study.

3.3.2 Secondary Outcomes

i. **Failure of treatment**. There is failure of treatment if one or more of the following events occur:

a) Death at any time after enrolment until hospital discharge, i.e. as primary outcome i., or

b) Initiation of life support at any time after enrolment until hospital discharge. Need for life support will be defined as a need for ventilation or vasoactive drugs at any time after enrolment until hospital discharge, and/or

c) A change in antibiotics for one of the following circumstances:

i) Persistence of signs that indicate ‘clinical severe infection’ present at the time of enrolment anytime after 48 hours of enrolment and prior to discharge.

ii) Worsening of existing signs or appearance of new signs of 'clinical severe infection' any time after enrolment and prior to discharge.

iii) Reappearance of signs of 'clinical severe infection' that the infant initially presented with at time of enrolment anytime after 48 hours of disappearance and prior to discharge

ii. **Cessation of signs of clinical severe infection**: This will be defined as the beginning of a 48 hour period with none of the signs of 'clinical severe infection'.

iii. **Discharge**: Defined as discharge from the hospital.

iv. **Death at any time after discharge from hospital until end of study period which is 12 weeks from the day of enrolment.**

v. **Severe illness requiring hospitalisation at any time after discharge from hospital until end of study period which is 12 weeks from the day of enrolment.**

vi. **Cost-effectiveness analysis:** To measure the incremental cost effectiveness ratio of zinc supplementation as an intervention for 'clinical severe infection' in infants < 2 months.

vii. **Immunobiological readouts**

4. TRIAL DESIGN

The trial will be conducted in compliance with the protocol and ICH (http://www.ich.org/home.html) guidelines.

The trial is a double-blind randomized placebo-controlled parallel group superiority trial. The trial will measure the efficacy of zinc administered orally as an adjunct to standard therapy to infants aged 1 day to 2 months hospitalized with clinical severe infection identified using an adaptation of the WHO Integrated Management of Childhood Illnesses (IMCI) criteria on reducing case fatality. The trial will also test the efficacy of zinc administered orally as an adjunct to standard therapy to infants aged 1 day to 2 months hospitalized with clinical severe infection on reducing time to death until end of the study period which is 12 weeks from the day of enrolment as another primary outcome.

This trial will also assess (i) failure of treatment, defined as a need to change antibiotics or requirement for life support or death, (ii) time to cessation of clinical symptoms and signs of clinical severe infection, (iii) time to failure of treatment and (iv) time to discharge (v) time to death (vi) Death at any time after discharge from hospital until end of study period which is 12 weeks from the day of enrolment (vii) Severe illness requiring hospitalization at any time after discharge from hospital until end of study period which is 12 weeks from the day of enrolment. An evaluation of the incremental cost effectiveness of zinc supplementation will be conducted to inform policy. This trial will also include mechanistic studies of the immune system during the episode of 'clinical severe infection'.

The participants will be randomized to receive zinc or placebo in a 1:1 allocation ratio. The intervention (zinc/ placebo drops) will be co-administered with the standard therapy which includes intravenous antibiotics and other supportive therapy like intravenous fluids, supplemental oxygen, etc. daily at 12 hrly intervals from the time of enrolment for 14 days. **4140 infants with clinical severe infection will be enrolled, given intervention for 14 days and followed up till discharge and until 12 weeks from the day of enrolment.**

Figure 1: Flow diagram of the trial

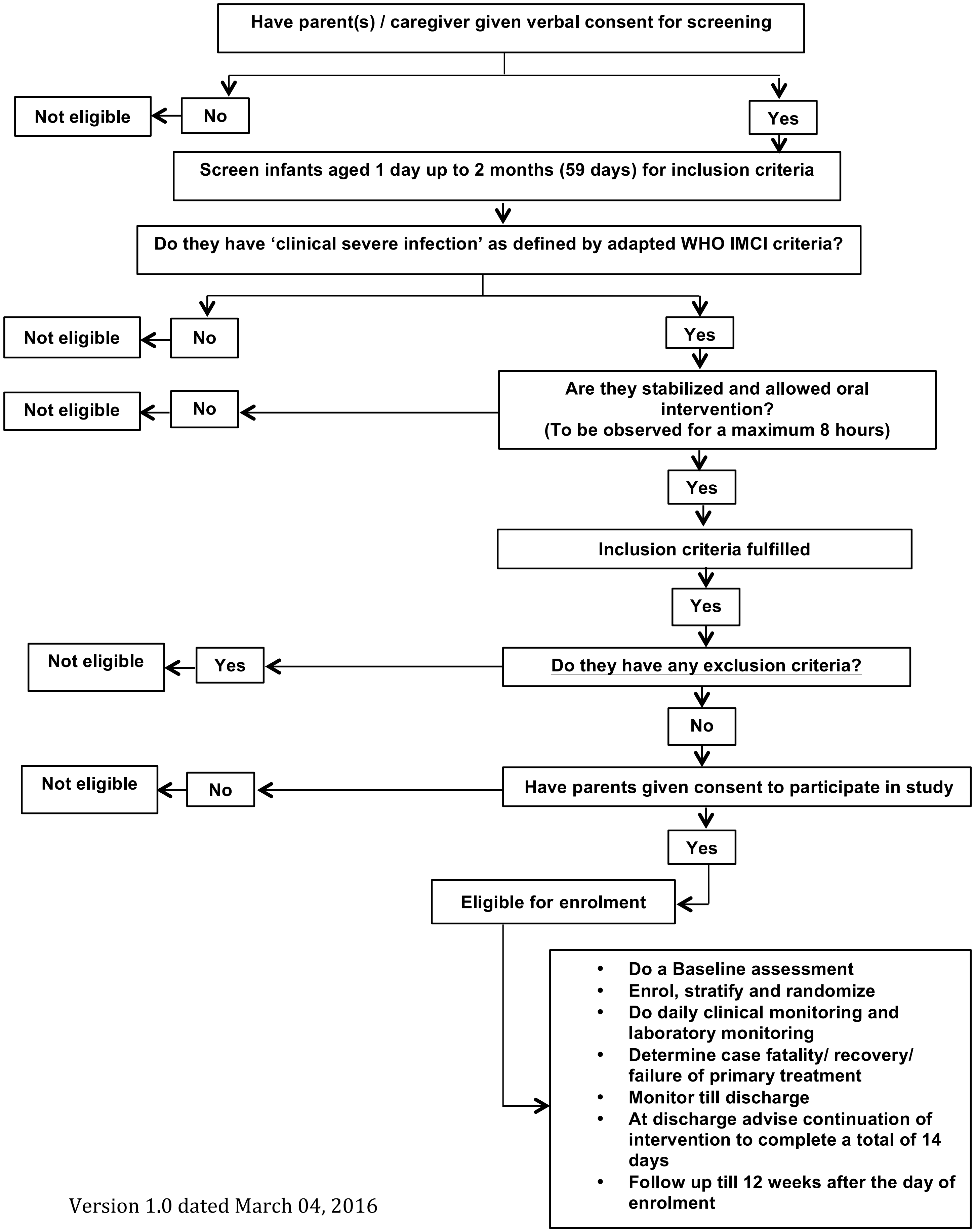

Version 1.0 dated March 04, 2016

5. Randomization

5.1. Stratification:

The infants fulfilling eligibility criteria will be **stratified by hospital and by the presence or absence of diarrhea**.

Diarrhea will be defined as passage of 3 or more liquid or watery stools per day. The normally frequent or loose stool of a breastfed baby is not diarrhea. The mother of a breastfed baby can recognize diarrhea because the consistency or frequency of the stools is different from normal.

5.2. Preparation and safe keeping of the randomization lists:

A statistician/ scientist not otherwise involved in the trial will generate the allocation sequences using STATA (StataCorp, College Station, Texas, USA) or equivalent software such that, within each hospital and stratum, patients are randomized in the ratio of 1:1 in permuted blocks of 8 to receive zinc or placebo. The randomization list will be generated for 4200 infants.

Duplicate copies of the randomization sequence will be kept safely with scientists not involved in the study and will not be available to any of the investigators until all data have been collected and cleaned, and the database is locked.

<u>Procedures for breaking the randomization codes:</u>

- The randomization codes and allocation sequence will be generated by an independent statistician/ scientist not otherwise related to the study
- The DSMB will be authorized to unblind the treatment allocation if required during the trial
- The DSMB may unblind under the following conditions:
  - An unanticipated serious adverse event (SAE) that has been judged to be related to the intervention by either the principal investigator/ designee
  - A SAE that has been judged to be related to the intervention and is of a severity or frequency higher than anticipated
  - An anticipated or other SAE where the management of the adverse event may depend on the intervention received
  - Other conditions where the DSMB may feel the need to unblind

5.3. Allocation concealment:

Identically looking zinc sulphate and placebo drops will be provided in identically looking bottles. The bottles will be labeled with a unique serial number according to the randomization list.

The study nurse/ medical officer will, after obtaining an initial consent for the screening, screen all infants coming to the emergency rooms of the hospital sites. Once eligibility has been determined and informed consent for participation in the trial obtained, a unique 'participant ID' will be assigned to the participant. The study nurse/ medical officer will be provided with the intervention bottles labelled with the unique participant ID.

Once the assigned bottle of intervention has been given to the participant, the study nurse/ medical officer will give 0.25 ml of the intervention drops (i.e. 5 mg elemental zinc) or placebo orally every 12 hours till discharge. At discharge the parents/

caregiver will be advised to continue with the intervention in the same dose to complete a total of 14 days of intervention

5.4. **Masking**:

The packing and appearance of the zinc sulphate and placebo drops will be identical. In addition the zinc sulphate and placebo drops will be similar in taste and appearance. This will ensure that the study team administering the intervention and ascertaining the outcome are unaware of group allocation.

` Masking will be maintained during data analysis by coding the treatment allocation with two letters.

5.5. Study Procedures

5.5.1. Study setting:

The trial will be conducted over a period of 3 years and 6 months. We will recruit the infants in 7 centres: **4 centres in Delhi, India, Maulana Azad Medical College (MAMC) and associated Lok Nayak Hospital, Vardhman Mahavir Medical College & Safdarjung Hospital (VMMC & SJH), Chacha Nehru Bal Chikitsalaya (CNBC) and Kasturba Hospital (KH)**; and **3 centres in Nepal, Patan Hospital [PH], Kathmandu, Kanti Children's Hospital (KCH), Kathmandu and Institute of Medicine (IOM), Kathmandu**. The selected hospital sites are secondary level hospitals with similar standard of care. This will make it easier to standardize study protocol across the hospitals. Each of the seven hospitals has a large patient load particularly of young infants with clinical severe infection. The implementation of the study will be coordinated by the Pediatric Biology Centre (PBC) at the Translational Health Science and Technology Institute (THSTI), in the National Capital Region (NCR), Delhi. The other collaborating centres will be the Tribhuvan University, Institute of Medicine, Kathmandu, Nepal Centre for International Health, University of Bergen, Norway (CHN-CIH), Innlandet Hospital Trust, Norway, Centres for Health Policy/Primary Care and Outcomes Research at Stanford University School of Medicine (CHP/PCOR) and Radbound University, Nijmegen, Netherlands. Tribhuvan University, Institute of Medicine, Kathmandu, Nepal will coordinate the implementation of the study at the Nepal hospital sites. CHN-CIH will play an advisory role and will help in establishing the 'Data Management Centre' for the study at THSTI and in data analysis. CHP/PCOR will collaborate with THSTI to measure the incremental cost effectiveness ratio of zinc supplementation as an intervention for clinical severe infection in infants <2 months. Radbound University will collaborate with THSTI Institute of Medicine Tribhuvan University and CHN-CIH for the immunological studies to explain potential mechanisms.

5.5.2. **Eligibility criteria**:

5.5.2.a. Inclusion criteria for participants

We have adapted the inclusion criteria from the WHO IMCI[39] and IMNCI[40] to identify very sick infants with clinical severe infection.[4] Infants aged 1 day and up to 2 months attending the emergency services of the hospitals will be screened by a study nurse (at the Indian hospital sites)/ medical officer (at the Nepal hospital sites) for the

following symptoms and signs and will be eligible for enrolment if **<u>any</u>** of these five signs are present:

i. **Stopped feeding well, defined as 'poor feeding on observation'**

An infant is said to have poor feeding on observation if:
<u>Exclusively breast-fed infant has</u>:

- Poor attachment to breast
- Suckling is not effective

<u>Not exclusively breast-fed/ non-breast fed infant</u>**:**
On observation of a feed given with cup and spoon, the infant does not accept the feed as vigorously as before (as per the mother's/ guardian's assessment)

ii. **Severe chest in drawing**:
When chest indrawing is present, the lower chest wall goes IN when the infant breathes IN.

Before the study nurse/ medical officer looks for chest indrawing she/ he must watch the infant to determine when the infant is breathing IN and when the infant is breathing OUT. If the infant's shirt was not lifted when she/ he counted the infant's breathes, the mother will be asked to lift it now. Study nurse/ medical office will look for chest indrawing when the infant breathes IN. She/ he will look at the lower chest wall (lower ribs). The infant has chest indrawing if the lower chest wall goes IN when the infant breathes IN. Chest indrawing occurs when the effort the infant needs to breathe IN is much greater than normal. In normal breathing, the whole chest wall (upper and lower) and the abdomen move OUT when the infant breathes IN. When chest indrawing is present, the lower chest wall goes IN when the infant breathes IN. If the study nurse/ medical officer is not sure that chest indrawing is present, she/ he will look again. If the infant's body is bent at the waist, it is hard to see the lower chest wall move. The mother will be asked to change the infant's position so he is lying flat in her lap. If she/ he still does not see the lower chest wall go IN when the infant breathes IN, the infant does not have chest indrawing. *For chest indrawing to be present, it must be clearly visible and present all the time.* If the study nurse/ medical officer see chest indrawing when the infant is crying or feeding, the infant does not have chest indrawing. *If only the soft tissue between the ribs goes in when the infant breathes in (also called intercostal indrawing or intercostal retractions), the infant does not have chest indrawing.* <u>In this assessment, chest indrawing is lower chest wall indrawing. It does not include "intercostal indrawing."</u>

iii. **Axillary temperature of ≥38.0°C on measurement,**
Fever with an axillary temperature ≥ **38.0°C** is uncommon in the first two months of life. If a young infant has fever, this may mean the infant has a severe infection. In addition, fever may be the only sign of a serious bacterial infection.

If the mother/ guardian gives a history of fever and on measurement the axillary temperature is <38^{0}C, the infant will be kept under observation for a maximum of 8 hours. Temperature measurement will be repeated every 4 hours or SOS during the observation period. If there is a documentation of axillary temperature of ≥ 38.0^{o}C during the observation period, the infant will be said to have fulfilled this inclusion criterion.
The thermometer will be kept high in the axilla and the infant's arm will be held against her/ his body for 3 full minutes before reading the temperature

iv. **Low body temperature (<35.5°C)**
Young infants can also respond to infection by dropping their body temperature to below 35.5ºC
A digital thermometer that measures to a minimum of 35ºC will be used to measure temperature.

v. **Movement only when stimulated**.
Young infants often sleep most of the time, and this is not a sign of illness. Even when awake, a healthy infant will usually not watch his mother and a physician/health worker while they talk, as an older infant or young child would.
A lethargic young infant who moves only when stimulated will *not be awake and alert when she/ he should be*. She/ He may be drowsy and may not awake after a disturbance.
If a young infant does not awake up during the assessment, the study nurse/ medical officer will flick the soles 2-3 times. She/ he will then look to see if the infant wakens and stays awake. If the young infant does not stay awake after some response, he is lethargic or said to have movement only when stimulated.

Infants who have any of these symptoms or signs and whose parents/ guardians have given written informed consent for participation will fulfill the inclusion criteria for enrolment. Those infants who fulfill the criteria of 'clinical severe infection' but are very sick and not allowed oral intervention will not be enrolled immediately but will be observed during a 'stabilization period' when they will be observed by the study nurse/ medical officer every 4th hourly for a maximum period of 8 hours of stabilization. Infants who have been stabilized and allowed orally, anytime within the 8 hour stabilization period; and who after stabilization continue to have at least one sign of 'clinical severe infection' and whose parents/ guardians have given written informed consent for participation will now fulfill the inclusion criteria.

5.5.2.b. Exclusion criteria for participants

The infants fulfilling the inclusion criteria **will be excluded** if they have the following conditions:

i. Weight at presentation < 1500g

The infant's current weight will be measured on an electronic infant weighing scale after the treating physician has examined the infant and the vitals are stable.

ii. **Weight for length <-3z at admission**
The infant's current length will be measured using an infantometer after the treating physician has examined the infant and the vitals are stable. The weight for length will be converted into Z-scores with the WHO growth reference. All infants with weight-for-length < -3z at the time of enrolment will be excluded. http://www.who.int/childgrowth/standards/cht_wfl_boys_z_0_2.pdf
http://www.who.int/childgrowth/standards/cht_wfl_girls_z_0_2.pdf

iii. **Surgical or other conditions that interfere with oral/ nasogastric feeding at admission.** For eg

a. Known structural defects, which interfere with feeding, e.g.

- esophageal abnormalities like esophageal atresia
- intestinal atresia and stenosis
- malrotation of the gut
- anorectal malformation like imperforate anus

b. Major congenital anomaly that interferes with feeding. Eg. anencephaly, omphalocele

c. Infants requiring mechanical ventilation for respiratory failure, shock

d. Infants started on inotropic support

e. Severe birth asphyxia (Apgar score of <4/10 at 5 min of birth)

iv. **Infants requiring surgical intervention or admission outside of pediatric ward for management** eg Open fractures, large meningomyelocele, massive burns, etc

v. **Infants requiring exchange transfusion on admission**

vi. **History of having received zinc in the last 48 hours**

We will examine the dose of elemental zinc that young infants in our area who receive multivitamin/micronutrient supplements (e.g. in the form of drops) are likely to ingest. If the likely dose exceeds 1 mg elemental zinc per day, babies receiving such supplements will not be included in the trial.

Any baby who has been started on zinc treatment for the current illness will not be included in the trial

vii. **History of having received injectable antibiotics for the last 48 hours or more before admission**

Note: The screening for eligibility will be done rapidly (within a couple of minutes) so that very little time is lost before starting the standard therapy for the 'Clinical severe infection'.

5.5.3. Intervention and Co-interventions

5.5.3.a. Intervention: Identically looking zinc sulphate drops (each ml of the formulation containing 20 mg of elemental zinc) and indistinguishable placebo drops will be provided in identically looking bottles. The bottles will be labeled with a 'unique serial number' according to the randomization list. A dropper with the 0.25 ml mark will accompany each bottle. The bottle will be stored in a cool, dry place.

Once eligibility has been determined and informed consent for participation in the trial obtained, she/ he will be randomized and assigned a unique 'participant ID' depending on the strata into which the infant is enrolled. The study nurse/ medical officer will take the intervention bottle labelled with the same unique participant ID and assign it to the enrolled infant.

Once the assigned bottle of intervention has been given to the participant, the study nurse/ medical officer will give 0.25 ml of the intervention drops (i.e. 5 mg elemental zinc) or placebo orally to the infant. The study nurse/ medical officer will be responsible for giving the assigned intervention to the infant at enrolment and then twice a day (every 12 hourly) till hospital discharge. The infant will be **observed for 30 min after each dose of intervention** that is administered. In case the infant vomits within the observation period, the intervention will be repeated. A maximum of two attempts will be made to administer each dose. If an infant is unable to tolerate two consecutive doses due to vomiting, that dose will be skipped and the next and subsequent doses will be given to the enrolled infant.

In case the enrolled infant is not allowed oral feeds and therefore cannot be given the intervention orally, the intervention will be given using a nasogastric tube as will the required feeds. The 0.25 ml volume of the intervention is minimal and can be given without any risk. The study staff will give the drops orally every 12 hours till discharge.

At discharge the parent (s)/ caregiver will be advised to continue with the intervention in the same dose to complete a total of 14 days of intervention. A maximum period of 14 days is based on previous studies where beneficial effects of zinc therapy beyond the period of administration have been seen when given for 14 days. The parent (s)/ caregiver will be advised to repeat the intervention if their baby vomits within 30 minutes of administration of the intervention.

The drops will be provided by an Indian pharmaceutical company Dr. Reddy's Laboratories Ltd (www.drreddys.com) at a reasonable price.
The study nurse/ medical officer will be responsible for administering each dose of zinc or placebo till discharge. This will ensure high compliance and small loss to follow-up till discharge.
In the few cases the enrolled infant develops necrotising enterocolitis or abdominal distension with tense abdomen or any such condition where the treating physician keeps the infant nil per orally and does not allow the intervention to be given either orally or by nasogastric route, the

intervention will be withheld until there is clinical improvement. Infants who receive 50% or more of the projected doses in the first five days after enrolment will be included in the per protocol analysis.

Our strategy of monitoring enrolled subjects at 6 hourly intervals and close interaction of the caregiver and the study staff will ensure compliance and minimize the loss to follow up and withdrawals. It will also ensure that the babies receive excellent clinical care. Close supervision by the Clinical Research Coordinator (CRC) and site investigators will minimize protocol deviation.

5.5.3.b. **Co-interventions:** The treatment of enrolled infants of 'clinical severe infection' with standard antibiotics and other medications will be given based on standard procedures. These procedures will be standardized across the 7 centres. The antibiotics received by the enrolled participant will be documented in the case reporting forms along with the route, dose and frequency.

All other therapy prescribed to the enrolled infant by the treating physician like supplemental oxygen, intravenous fluids, multivitamins, etc. will also be documented in the case reporting forms along with the route, dose and frequency.

Care will be taken that the enrolled infant is not prescribed 'zinc' inadvertently particularly in the multivitamin drops or the creams for local application for perianal rash.

Note: Zinc is not recommended as standard treatment for diarrhea to infants< 2 months of age

5.5.3.c. Contraindications to subsequent doses of intervention

5.5.3.d. Procedures for Monitoring safety and participant compliance

Refer to sections 5.5.5.c and 8

5.5.4. Outcomes

5.5.4.a. <u>Primary Outcomes</u>

i. To estimate the efficacy of 10 mg elemental zinc administered orally as an adjunct to standard antibiotic therapy to infants aged 1 day up to 2 months (59 days) hospitalized with 'clinical severe infection' against **case fatality**.
The case fatality risk is the proportion of children with 'clinical severe infection' who die due to any cause and at any time from enrolment until hospital discharge.
ii. **Time to death until end of study period** defined as the time from enrolment to the time when an infant dies until 12 weeks from the day of enrolment in the study.

5.5.4.b. Secondary Outcomes

i. **Failure of treatment**. There is failure of treatment if one or more of the following events occur:

   a) Death at any time after enrolment until hospital discharge, i.e. as primary outcome i., or

   b) Initiation of life support at any time after enrolment until hospital discharge. Need for life support will be defined as a need for ventilation or vasoactive drugs at any time after enrolment until hospital discharge, and/or

   c) A change in antibiotics for one of the following circumstances:

      i) Persistence of signs that indicate 'clinical severe infection' present at the time of enrolment anytime after 48 hours of enrolment and prior to discharge.

      ii) Worsening of existing signs or appearance of new signs of 'clinical severe infection' any time after enrolment and prior to discharge.

      iii) Reappearance of signs of 'clinical severe infection' that the infant initially presented with at time of enrolment anytime after 48 hours of disappearance and prior to discharge

ii. **Cessation of signs of clinical severe infection**: This will be defined as the beginning of a 48 hour period with none of the signs of 'clinical severe infection'.

iii. **Discharge**: Defined as discharge from the hospital.

iv. **Death at any time after discharge from hospital until end of study period which is 12 weeks from the day of enrolment.**

v. **Severe illness requiring hospitalisation at any time after discharge from hospital until end of study period which is 12 weeks from the day of enrolment.**

vi. **Cost-effectiveness analysis:** To measure the incremental cost effectiveness ratio of zinc supplementation as an intervention for 'clinical severe infection' in infants < 2 months.

vii. **Immunobiological markers**

5.5.5. Data collection

5.5.5.a. Description of data arising from proposed research: The study nurses/medical officers will collect and safely store the data including patient eligibility, informed consent statements and clinical outcomes**.** The data will consist of baseline information on clinical and demographic characteristics of infants screened and enrolled in the study and details of 6 hourly progress including intervention, co-interventions, progress of clinical illness resulting in recovery, treatment failure or death during the hospitalization.

5.5.5.b. Data collection, including type, format, scale and standards for data: All the data will be collected in standard forms electronically at the seven sites by the research nurses/ medical officers supervised by the study physician (research officer). The forms will be developed by the data management centre (DMC) and will consist of details of all the variables with standard definitions and labels.

5.5.5.c. Clinical Data Collection

Study nurses/ medical officers will examine the enrolled infants for all clinical features of clinical severe infection every 6 hours or more often if clinically indicated, until discharge from the hospital. They will repeat the respiratory rate count if it is $\geq$ 60/min and the lowest of the two counts will be recorded. They will also record nude weights at enrolment and every 24 hours till discharge. The research officers (pediatricians) will oversee at least one daily monitoring conducted by each study nurse/ medical officer. The decisions for all outcome measures will be made by the research officer in consultation with an experienced pediatrician (site investigator) based on the closely supervised 6 hourly monitoring done by the study nurse/ medical officer.

At the time of discharge the parents/ caregiver will be given a participant booklet and advise on the following:

i. To continue the intervention in the same dose to complete a total of 14 days of the intervention. The participant booklet will have a log, which the parent/ caregiver will be asked to fill each time they administer the intervention.
ii. To repeat the dose in case the infant vomits within 30 min of having received a dose of intervention. In case there is vomiting within 30 min of repeat dose of intervention, the parent/ caregiver will be advised to skip that dose.
iii. They will be asked to make a follow up visit to the hospital site 10-15 days post their discharge, after they have completed the 14 day course of intervention and asked to get the intervention bottle with them and the participant booklet. At the follow up visit data on the infant's health will be collected and a 'pill count' will be made to check the compliance.
iv. They will also be advised that the study staff will be contacting them twice telephonically in the next 3 months to collect data on the infant's health.
v. List of situations when the parent/ caregiver would need to contact the study team
vi. Contact numbers of the study team
vii. Advise on immunisation schedule

Once the enrolled infant with 'clinical severe infection' has recovered and been discharged from the hospital, the parents of the infant will be contacted <u>on telephon</u>e at <u>one and a half month</u> and again at <u>three months from the day of enrolment</u>. During the post discharge telephonic contact the following information will be collected using structured forms

i. Infants' health and well being including the feeding pattern, any difficulties in feeding, frequency of urine and sleep pattern and routine immunization.
ii. Any illness in form of signs and symptoms of 'clinical severe infection' since last contact
iii. Any hospitalisation since last contact. If yes, then details of hospitalization-diagnosis and duration of hospitalization. This will help us differentiate between accidents/ non-accidental indications for hospitalization.
iv. Death of infant. In case of death, validated verbal autopsy questionnaires that will help us differentiate between death due to an accident or an illness/ non-accidental cause.

The study nurses/ medical officers will collect all this post discharge information. They will be trained in establishing telephonic contact with the parents and collecting all the relevant information as per the questionnaire. Parents/ caregivers of infants will be encouraged to report any hospitalization within the 3-month study period post discharge to the study team through a visit to the hospital site or a call made to the team.

5.6. Standard Case Management

Infants will be treated according to a standardized treatment protocol across all centres; recommended doses of intravenous ampicillin or amoxicillin-clavulinic acid and an aminoglycoside (amikacin or gentamicin) will be given when infants come without antibiotic treatment (or on oral antibiotics) to the study hospitals, or a third generation cephalosporin (cefotaxime or ceftriaxone) and an aminoglycoside (amikacin or gentamicin) when infants have already been treated with these injectable antibiotics (for $\geq$ 48 hours) prior to admission. In case of suspected meningitis ceftriaxone or cefotaxime and amikacin will be started on admission. In case of suspected staphylococcal infection, cloxacillin or amoxicillin-clavulinic acid will be given with an aminoglycoside and in suspected staphylococcal meningitis vancomycin and amikacin or gentamicin will be administered. The duration of antimicrobial therapy will be 7-10 days, extendable to three weeks in meningitis. Intravenous fluids, temperature maintenance and oxygen will be provided as supportive therapy. The treating physicians who are also site investigators will be responsible for the medical treatment of the enrolled infants with clinical severe infection. The study staff will be responsible for clinical and safety monitoring as described later in the protocol.

5.7. Laboratory procedures

Bio-specimens

Study nurses/ medical officers will collect blood specimens at baseline (enrolment), at 48-72 hours of enrolment and at discharge.

The following is the schedule for peripheral blood collection for each enrolled participant at different time points:

i. <u>At enrolment</u>: 3-5 ml of blood will be collected intravenously for
   1. Blood culture by BACTEC
   2. Septic screen which includes

i) total leukocyte count (TLC),
ii) absolute neutrophil count (ANC),
iii) C-reactive protein (CRP),
iv) micro ESR and
v) band cell: neutrophil count ratio (I:T ratio).

3. Baseline serum zinc estimation
4. Soluble markers of inflammation in serum
5. Etiology and characterization of viral pathogens in ‘clinical severe infection’
6. Immunobiological studies
   i) Cell stimulation assays for measuring inflammatory response (cytokine levels)
   ii) DNA isolation for methylation studies
   iii) RNA isolation for transcriptional studies

A total of up to **3-5 ml of blood** will be collected at admission (enrolment). This will include the blood required for standard of care like **1 ml blood for blood culture** by BACTEC, another **1 ml blood for septic screening** which includes CRP, TLC, ANC, mESR and I: T ratio. All investigations necessary for standard care will be done but with techniques that use smaller volumes of blood.
The remaining blood will be used for immunobiologic studies mentioned above.
1 ml blood will be used for isolation of sera for estimation of **zinc and soluble markers of inflammation.**
1 ml fresh blood will be used for **cell stimulation assays** to be done using either fresh whole blood or by isolating PBMCs within 16 hours of collection. The culture supernatant from cell stimulation assays will be stored for cytokine level estimation.
The **remaining 1 ml** will be used for **DNA isolation for methylation studies (0.5 ml) and RNA isolation for transcriptional studies (0.5 ml)**.

Indian hospital sites: Blood samples for blood culture by BACTEC and septic screen (except CRP) will be processed and analysed at the respective Indian hospital sites or at an accredited laboratory in the close vicinity of the hospital.
Nasal swab/ nasopharyngeal aspirate for the etiologic studies will be transported to the central laboratory at THSTI in the National Capital Region (NCR) at the earliest on ice (dry-ice if available) and will be stored in - 70^0C to -80^0C deep freezers till analysis.
Blood for the immunobiological studies will be transported at room temperature (37^0C) to the central laboratory at THSTI in the National Capital Region (NCR) at the earliest and definitely within 16 hours of collection and after the stimulation assays the culture supernatant will be stored in -70^0C to -80^0C deep freezers till analysis for analysis of inflammatory markers (cytokine levels).
The sera for zinc and soluble markers of inflammation will be separated at the hospital site and then transported on ice to the central laboratory at THSTI where it will be stored at -70^0C to - 80^0C till analysis.
Nepal hospital sites: Blood samples for blood culture by BACTEC and septic screen (except CRP) will be processed and analysed at the respective Nepal hospital sites or at an accredited laboratory in the close vicinity of the hospital.

Nasal swab/ nasopharyngeal aspirate for the etiologic studies will be transported to the central laboratory at Institute of Medicine in Kathmandu, Nepal at the earliest on ice (dry-ice if available) and will be stored in - 70^0C to -80^0C deep freezers till analysis.
Blood for the immunobiological studies will be transported at room temperature (37^0C) to the central laboratory at Institute of Medicine (IOM) in Kathmandu, Nepal at the earliest and definitely within 16 hours of collection and after the stimulation assays the culture supernatant will be stored in -70^0C to - 80^0C deep freezers till analysis for cytokine levels.
The sera for zinc and soluble markers of inflammation will be separated at the hospital site and then transported on ice to the central laboratory at IOM, Kathmandu, Nepal.
The samples for CRP, zinc estimation, soluble markers for inflammation and cell stimulation assays for cytokine levels will be processed on site/ at the central facility at IOM and transported for temporary storage in the -70^0C to - 80^0C deep freezer at the Institute of Medicine. These samples will be transported to the central laboratory at THSTI in India twice a year.

ii. At <u>48-72 hrs of enrolment</u>: 2-3 ml of blood will be collected intravenously for
   a) Soluble markers of inflammation in serum
   b) Immunobiological studies
      i) Cell stimulation assays for measuring inflammatory response (cytokine levels)
      ii) DNA isolation for methylation studies
      iii) RNA isolation for transcriptional studies

The study team will try to coincide the blood sampling for the 48-72 hours time point with the routine sampling done in case of treatment failure or to see the progress in biochemical parameters of the sick infant. In case the participant does not require undergoing a routine sampling by 72 hours of enrolment, the study sample will be collected without any further delay.

iii. <u>At discharge</u>: 2-3 ml blood will be collected intravenously for
   a) Soluble markers of inflammation in serum
   b) Immunobiological studies
      i) Cell stimulation assays for measuring inflammatory response (cytokine levels)
      ii) DNA isolation for methylation studies
      iii) RNA isolation for transcriptional studies

Serum concentrations of zinc will be measured in a subset of the enrolled infants using standard procedures. The zinc concentration will be analyzed using a flame furnace atomic absorption spectrophotometer (Thermo Scientific) using standard techniques. Serum CRP concentration will be measured using a commercial ELISA kit at the micronutrient lab at THSTI. Blood cultures will be done at enrolment using BACTEC (Becton Dickinson, Sparks, 152 MD, U.S.A.) at each hospital site. Blood collection for immunobiological studies and for soluble markers of inflammation will be done at enrolment, 48-72 hrs and discharge.

INVESTIGATION CHECKLIST

(Please tick and write date whenever any investigation is sent)

BIOSPECIMEN COLLECTION	0 HOURS / /	48-72 HOURS / /	DISCHARGE / /
	Volume of blood 3-5ml	Volume of blood 2-3ml	Volume of blood 2-3ml
**1.** Blood culture by BACTEC			
**2. Septic Screening**			
(i) TLC			
(ii) ANC			
(iii) CRP			
(iv) mESR			
(v) 1: T ratio			
**3.** Cell stimulation assay for cytokine levels			
**4. Storage of bio specimens**			
(i) Zinc estimation			
(ii) Soluble markers of inflammation in serum			
(iii) DNA isolation from blood for methylation studies			
(iv) RNA isolation from blood for transcriptional studies			
(v) Nasal swab / NPA for viral etiology			

6. Supplies and Handling of Materials

Intervention: Zinc/Placebo

Zinc and placebo drops will be procured from 'Dr Reddy's Laboratories'. The preparation of the intervention will contain 20 mg of elemental zinc as zinc sulphate per ml of the drops. The placebo preparation will contain the same ingredients as the zinc sulphate preparation. The only way it will differ from the zinc sulphate preparation will be the zinc content. The zinc or placebo drops will be similar in color, taste and appearance. They will be provided in similar looking bottles labeled with similar labels except for the 'unique ID' on each bottle. A small dropper that will accompany each bottle will dispense the drops. The 1 ml dropper accompanying the bottle will have a line that marks 0.25 and 0.5 ml. One 15 ml bottle of intervention labeled with a unique ID along with a dropper will be packed for each enrolled infant in a box. Each box including the bottle and dropper, with either zinc or placebo will be labeled with the same unique ID as on the bottle and this will correspond to the randomization list.

The intervention will be shipped/ couriered by Dr Reddy's Laboratories to the coordination centre at THSTI at room temperature with a data logger. At the coordination centre a team not involved in the trial otherwise will do the labeling of the boxes containing the bottle and dropper and of the bottles with the generated unique IDs. This team will receive a copy of the randomization sequence from the scientist/ statistician involved in generating the allocation sequence for the study.
Once the labeling is complete, the intervention boxes will be stored in the intervention room at the Pediatric Biology Centre, THSTI.
The required number of intervention bottles will be transported from the coordination centre at THSTI to the hospital sites in New Delhi, India and Kathmandu, Nepal at room temperature with a data logger. For the study purpose 28 doses of 0.25 ml each (total of 7 ml) will be taken from the 15 ml bottle over a period of 14 days for oral administration and the remaining will be discarded.

6.1. Labeling

The text of the label will be common to both the bottles containing zinc or placebo.
The label text will be based on masking requirements and have the following:

i. Participant ID
ii. A caution that the drug is limited for investigational use
iii. To be stored in dry and cool place
iv. Total volume of intervention contained in the bottle
v. Manufacturing license No
vi. Batch No
vii. Manufacturing date
viii. Date of expiry
ix. Manufacturer's name, address, telephone number
x. For oral administration only
xi. Title of study
xii. Protocol number

xiii. Name of contact person

6.2. Storage

The intervention (zinc / placebo) will be stored at room temperature in a dry and cool place. All the supplies will be stored at the central storage facility at the coordination centre at THSTI.
Each bottle of intervention and its accompanying box will be labeled with a unique ID. Once an infant is enrolled in the study and assigned a unique participant ID, the corresponding intervention bottle and box will be marked with the date and time of randomization.
Once assigned to a participant, the intervention will be kept in a dry place.

6.3. Clinical Supplies Accountability

The details of the intervention bottles with their boxes received at the coordination centre will be recorded at receipt twice- first from the manufacturer and then from the team that has labeled the bottles and boxes. An inventory of the bottles will be maintained along with the remaining supplies by the 'coordination centre team'.
This team will also maintain a log of intervention allocated.
The used intervention bottles will be stored in a locked cupboard for intervention accountability verification by monitors. These bottles will be will be stored till the study is complete and the data published. The bottles will be destroyed after instructions from the PMT.

6.4. Source Documents

The database will not include participant name or personal identifying information other than the participant ID, age of infant and initials. The case reporting forms (CRFs) will have an identification form with the name of the participant, address, contact details, etc. This form will be used at the time of follow up, post discharge. This information will not be entered in the database and will not be linked to the other data captured from the participant.
The CRFs (paper/ electronic) will serve as source documents for all data, till the participant is enrolled in study and in the hospital till recovery and discharge. In addition the laboratory forms from the hospital records will serve as source documents for the laboratory reports. After discharge OPD slips, hospitalization summaries and laboratory reports will serve as source documents for the follow up data collection apart from the telephonically collected data on CRFs.

7. WITHDRAWAL CRITERIA

Censorship/Withdrawal Criteria

The enrolled participants will be censored / considered to be withdrawn from the study in the following circumstances:

i. The family of the participant may wish to withdraw consent from the trial at any point during follow up.

In case the family withdraws consent for blood samples to be taken for the purpose of the study but not for clinical monitoring and outcome assessment, the participant will not be considered to be withdrawn from study.

ii. Participant may take discharge against medical advice and leave the hospital before outcome assessment (death or recovery). The study team will attempt to contact the parent (s) on telephone and collect information on the primary outcome of death. But it may not always be feasible to follow up participant for the outcome assessment.
iii. Completion of study period as defined by the protocol that is end 12 weeks from the day of enrolment without outcome (that is either recovery from 'clinical severe infection' or death).

For participants who are withdrawn from the trial prior to completion of follow up, attempts will be made to assess the outcome and/or collect blood specimens as far as feasible, in addition to information on the reason for discontinuation.

8. SAFETY MONITORING

8.1. Adverse Events

8.1.1. Adverse Events (AE)

ICH E6 Good Clinical Practice Guidelines defines an adverse event (AE) as any untoward medical occurrence in a patient or clinical investigation participant, administered a pharmaceutical product that does not necessarily have a causal relationship with this treatment. An AE can therefore be any unfavorable and unintended sign (including an abnormal laboratory finding), symptom or disease temporally associated with the use of intervention whether or not it is related to the intervention.

These adverse events may be expected or unexpected. The former is any adverse reaction whose nature and severity have been previously observed and documented for the intervention. Although for this intervention, there are no expected adverse events, the commonly reported adverse events will be documented. An unexpected adverse event is any adverse event not previously reported.

In this trial the following will be included as adverse events:

i. Regurgitation or vomiting within 30 minutes of administration of first dose of intervention
ii. Regurgitation or vomiting within 30 minutes of administration of subsequent dose/s? of intervention
iii. Vomiting any time during the study period
iv. Abdominal distension lasting > 24 hours any time during the study period
v. Acute refusal of feed related to the intervention (within 4-6 hours)
vi. Change of antibiotics due to persistence of signs of 'clinical severe infection' anytime after 48 hours of enrolment and prior to discharge

8.1.2. Serious Adverse Event (SAE)

A Serious Adverse Event (study specific) is defined as any untoward medical occurrence in the enrolled participant, that at any dose:

i. Results in death anytime during the study period
ii. Is life threatening (defined as a participant at immediate risk of death at the time of the event). For eg Initiation of life support
iii. Requires inpatient hospitalization (if not already hospitalized) or prolongation of existing hospital admission
   Prolongation of hospital admission during the study period can be due to
   a) worsening of signs of clinical severe infection;
   b) appearance of new signs of clinical severe infection ;
   c) reappearance of signs of clinical severe infection after a 48 hours symptom free period
   d) development of signs of critical illness like unconsciousness, convulsions, apnea, unable to feed at all, cyanosis, dehydration, bulging fontanelle, persistent vomiting, low pulse volume, delayed capillary refill time
   e) appearance of new symptom or any unexpected event which could lead to prolongation of hospital stay like injection abscess or fall from cot leading to fracture or head injury

The study staff will be responsible for monitoring of all SAEs and AEs.

8.2. Relatedness to Intervention

Very likely/Certain: A clinical event with a plausible time relationship to administration of intervention and which cannot be explained by concurrent disease or other drugs or chemicals

Probable: A clinical event with a reasonable time relationship to administration of intervention; is unlikely to be attributed to concurrent disease or other drugs or chemicals.

Possible: A clinical event with a reasonable time relationship to administration of intervention, but which could be explained by concurrent disease or other drugs or chemicals

Unlikely: A clinical event whose time relationship to administration of intervention makes a causal connection improbable, but which could be plausible explained by underlying disease or other drugs or chemicals.

Unrelated: A clinical event with an incompatible time relationship and which could be explained by underlying disease or other drugs or chemicals.

Unclassifiable: A clinical event with insufficient information to permit assessment and identification of the cause.

8.3. Specification of Safety Parameters

The study nurse/ medical officer will be responsible for monitoring and recording of all AEs. All immediate adverse events following each dose of intervention (including regurgitation or vomiting within 30 minutes of administration of first or subsequent dose of intervention, vomiting any time during the study period, abdominal distension lasting > 24 hours any time during the study or acute refusal of feed related to the intervention (within 4-6 hours)) will be documented for all infants. During the pre defined 6 hourly clinical monitorings any worsening in condition of infant or appearance of new symptom or any unexpected event which could lead to prolongation of hospital stay like injection abscess or fall from cot leading to fracture or head injury will be recorded in the CRF. The study nurse/ medical officer will also make three follow-up contacts after discharge of infant from hospital. The first follow up will be in the hospital and the subsequent two follow up contacts will be on phone. During each contact the study staff will collect data to ascertain SAEs, illness requiring hospital referral and In case the infant has any of the illness requiring hospital referral, the infant will be advised to visit the hospital site for management.

Serious adverse events will be documented from the time of enrolment, throughout the study period, and reported to the coordinating centre IEC and the site IEC as per their requirements and to the sponsor/designee within 24 hours of being aware of the event. The coordinating centre IEC at THSTI will send the final event description of the death SAEs along with the causality assessment and compensation (if required) to funding agencies and sponsor within 30 days of the event.

SAE relatedness will be judged by the investigator/ designee and the DSMB who will have access to all relevant investigations, clinical assessments and management details.

8.4. Data Safety Monitoring Board

There will be two DSMBs, one for the Indian hospital sites and one for the Nepal hospital sites. The DSMB for the Indian hospital sites will comprise of a pediatrician, intensive care physician, an epidemiologist and a biostatistician. The DSMB for the Nepal hospital sites will comprise of three members including a statistician and a pediatrician. One of the DSMB members can be overlapping in both the India and Nepal DSMBs. These DSMBs will be independent from the sponsor and will have no competing interests. The DSMBs will prepare a charter and decide *a priori* on study stopping rules, and will review SAE and AE reported in the study periodically. They will examine all infant deaths and other SAE to decide if the study should be continued, based on the pre-decided stopping rules. After one third of the study participants have been enrolled and completed follow-up, the independent DSMBs will review the data and make recommendations concerning continuation, modification or termination of the study due to unexpectedly large beneficial effects or serious side effects.

Feedback to Data Safety Monitoring Board (DSMB): A feedback form for the DSMBs containing relevant information will be prepared at the data management centre in consultation with the CRC, PBC PI, Nepal PI and the site investigators, (DMC) and sent to the DSMB at regular intervals.

8.4.1. Safety Advisor

A pediatrician will be designated as the safety advisor for this trial. All immediate adverse events and deaths will be reviewed by the safety advisor. Also, the team may obtain his/her opinion on any other issue related to the safety of enrolled infants. The safety advisor will be a part of the DSMB.

8.5. Independent study monitoring

An independent monitor chosen by and paid for by CISMAC will be responsible for the monitoring of the study**.** The monitoring plan will include predefined periodic visits to all recruitment sites by the independent monitors who will review every aspect of the study using standardized formats. The conclusions/recommendations arising from these monitoring visits will be communicated to the clinical research coordinator (CRC), PBC PI, Nepal PI and site PIs for corrective and preventive action.

8.6. Adverse event reporting

Zinc has been shown to be safe in doses ranging from 5–45 mg per day when it has been used in both the treatment of acute diarrhea as short-term therapy and also as long-term supplementation in infants and young children in Asia, Latin America, and Africa. [18-25] All zinc salts (sulfate, acetate, and gluconate) have been found to be safe.[24] Zinc-treated infants in our own earlier trial [8] for treatment of probable serious bacterial infection did not demonstrate any adverse effects events. In addition to standard care provided by the hospital physicians the study physicians will examine the enrolled infants for any related adverse effects as given below at the time of giving the intervention and every 6 hours, or more often if indicated, until discharge from hospital and also till the infant completes the 14 days of the intervention.

All **serious adverse events (SAE)** of death, SAE other than death and initiation of life support (which are also the study outcomes) and **adverse events (AE)** as described above will be reported to the Ethics Committees, the DSMB (see below) and the sponsors of the study within the specified time period.

Serious adverse events will be documented from the time of enrolment, throughout the study period, and reported to the coordinating centre IEC and the site IEC as per their requirements and to the sponsor/designee (DSMB) within 24 hours of being aware of the event. The coordinating centre IEC at THSTI will send the event description of the SAEs to the Drugs Controller General of India within the timelines prescribed in Schedule Y of the Drugs and Cosmetics Act 2005. Information that will be collected includes event description, time of onset, investigator assessment of severity, relationship to study product, time of resolution of the event, seriousness, and outcome. All serious adverse events occurring in study participants will be documented appropriately regardless of relationship and followed up to adequate resolution or stabilization as judged by the treating physician and the Principal Investigator of the site.

All adverse events not meeting the criteria for “serious adverse events” will be captured on the appropriate case report form. All adverse events will be graded for intensity and relationship to the intervention. In addition each adverse event will be

assessed to be serious (SAE) or not. Adverse events will be documented and reported to the coordinating centre IEC and the site IEC periodically and to the DSMB as per their requirement.

8.7. Follow Up After Adverse Events

All adverse events will be followed up till resolution or stabilization as judged by the treating pediatrician (site investigator) and the principal investigator. All SAEs will be followed up until satisfactory resolution or until the treating pediatrician and the principal investigator deem the event to be chronic or participant to be stable.

8.8. Trial Termination

The study may be stopped for ethical reasons at the recommendation of the institutional ethics committees of any of the partner institutions/ hospital sites or for safety reasons at the recommendations of the DSMB, or funding agencies in which case no further dose of the intervention will be given but follow up of enrolled participants will continue till the end of study period as scheduled.

9. Ethical Issues and Approvals

9.1. Ethical issues:

i. The drug which is the study intervention (elemental zinc given as zinc sulphate drops orally in the dose of 10 mg per day for 14 days) is already approved for use in infants with diarrhea. This study extends the use of the intervention to other illnesses at the same dosage of 10 mg per day and for the same duration. Therefore there is no ethical issue about its use. Additionally, most infants with diarrhea in this age group of less than 2 months have systemic illness.

ii. As mentioned above, the study intervention (elemental zinc given as zinc sulphate drops orally in the dose of 10 mg per day for 14 days) is already approved for use in infants with diarrhea. Therefore, as per the Circular (File No. 12-01/14-DC (pt. 47)) from the DCGI dated 10-11-2015, since this trial is not being done for claiming permission of New Drug for marketing as per Drugs and Cosmetic Rules, permission from DCG (I) shall not be required provided that the trial has been approved by the respective Ethics Committee. (Circular attached as **Annexure I**).

9.2. Ethical Approvals

The Investigators will initiate the study only after securing approval from the Institutional Ethics Committees of all the seven hospital sites in which the study is to be carried out as well as from THSTI IEC, NHRC Nepal and REC West in Norway.

10. STATISTICS

10.1. Sample Size

Based on the most recent annual audits undertaken in the district hospitals in India, the case fatality risk in infants < 2 months with clinical severe infection is expected to be 10%. Assuming a 10% loss to follow-up, we will need to recruit 4,140 infants

(2,070 in each group) to identify a clinically important (≥30%) risk reduction of death by administering zinc, with 90% power and 5% confidence.

With the proposed sample size we will have sufficient power to detect a difference in time to death from enrolment until 12 weeks post discharge. Although a secondary outcome, with the proposed sample size, we will be able to detect even a 17% relative risk reduction in treatment failure between the two groups at 90% power assuming the risk of treatment failure in the placebo arm to be 25%.

10.2. Analysis

10.2.1. Plan of analysis

10.2.1.a Plan of analysis for the primary outcome:

The **primary outcome** is **case fatality**. Using statistical software for analysis (STATA), the proportion of children who die will be calculated in the two study (zinc and placebo) arms. This will give us the risk in the two study arms. Relative risks (RRs), risk differences (RDs) and numbers needed to treat (NNT), and their corresponding 95% confidence intervals (CIs) will be calculated. This will be done using an intention-to-treat approach where all infants assigned into the zinc or placebo group will be analyzed. There will be a systematic registration of all baseline characteristics. In case the baseline variables associated with the primary outcome are unevenly distributed between the two study arms, we will adjust the RRs and RDs/NNT for these baseline imbalances, using generalized linear models of the binomial family with log and identity links, respectively.

10.2.1.b Secondary outcomes:

Likewise, we will compare the risks in the intervention and placebo arms of the trial, for the secondary outcomes like **treatment failure**, **death at any time after discharge from hospital until end of study period which is 12 weeks from the day of enrolment, severe illness requiring hospitalisation at any time after discharge from hospital until end of study period**.

For the secondary outcome of treatment failure we will compare the risk in the intervention and placebo arms of the trial using

i. Composite definition of treatment failure

ii. Cause specific treatment failure.

   Cause of treatment failure will be assigned by the worst outcome and the first cause of failure.

iii. Treatment failure where suspected nosocomial infections will be excluded. Nosocomial infections will be suspected when antibiotics are changed for

   a. Persistence of signs of ‘clinical severe infection’ beyond 168 completed hours,

b. Worsening of signs of ‘clinical severe infection’ after 168 completed hours and
c. Reappearance of signs of ‘clinical severe infection’ 48 hours after a symptom free period

These secondary outcomes will be analyzed the same way as the primary outcomes. If an outcome-event can occur more than once, we will adjust for the repeated observations in statistical models. The secondary outcome of severe illness requiring hospitalization will also be analyzed in Poisson or negative binomial regression models (allowing for more than one event per child).
Percent efficacy will be calculated using one of these forumlas: 100×(1-relative risk) or 100×(incidence rate ratio), as appropriate.

10.2.1.c Time-to-event analyses will be done for the following **primary and secondary outcomes**:

Time to death until end of study period defined as the time from enrolment to the time when an infant dies anytime until 12 weeks from the day of enrolment in the study**:** This analysis will consider the event as death at any time after enrolment until 12 weeks from the day of enrolment into the study. Again, this will be done using an intention-to-treat approach where all infants assigned into the zinc or placebo group will be analyzed. Infants where the parent/ guardian withdraws consent for continuation in the study or for other reasons cannot be followed until 12 weeks after enrolment in the study will be censored at the time point that they were lost to follow up. Data on these lost to follow up infants till the time of censoring will be considered during the analysis. We will use a Cox proportional hazards model to compare the time to death anytime after enrolment until end of study period, which is 12 weeks from the day of enrolment between study groups. The hazard ratio (HR) and their corresponding 95% confidence intervals (CIs) will be calculated.
Time to death: The event will be death at any time after enrolment until hospital discharge. Infants where the caretaker withdraws consent for continuation in the study or for other reasons cannot be followed until recovery and discharge will be censored. This will also be done using an intention-to-treat approach where all infants assigned into the zinc or placebo group will be analyzed. We will use a Cox proportional hazards model to compare the time to death until hospital discharge between study groups and will calculate the relative hazard of death during hospitalization between the intervention groups in (i.e. comparing time until death during hospitalization).
Time to cessation of signs of clinical severe infection: The event will be cessation of signs of clinical severe infection. Infants, where the caretaker withdraws consent for continuation or for other reasons cannot be followed further, will be censored. Irrespective of whether treatment failure has occurred, we will use a Cox proportional hazards model to compare the time to cessation of signs of clinical severe infection between study groups.
Time to failure of treatment: This analysis will consider the event as treatment failure. Censoring infants who withdraw consent to continue in the

trial we will use a Cox proportional hazards model to compare the time to treatment failure between study groups.

Time to discharge: This analysis will consider the event as discharge. Censoring infants who withdraw consent for continuation in the trial, we will use a Cox proportional hazards model to compare the time to discharge between study groups.

Percent efficacy in time-to-the events analyses is 100×(1-hazard ratio).

Like antibiotics, zinc is unlikely to exert an effect before 24 hours of administration. All of the above analyses will therefore be repeated where the outcomes are redefined to occur only when the event takes place after 24 hours of administering the first dose of zinc.

10.2.1.d Analyses of laboratory parameters:

Serum zinc concentration and **immunbiological readouts** at recovery and the change from baseline to recovery will be compared between the two groups using Student's t-test or, if need to adjust for potential confounders, with linear regression.

10.2.1.e Subgroup analyses

Subgroup analyses to study effects of zinc on primary and secondary outcomes will be done (i) within each hospital, (ii) for whether the child had diarrhea (on admission) to assess whether its presence at enrolment modifies the effect of zinc [8] (iii) in those less than 7 days old and (iv) in those with a positive septic screen (adapted from Center for Disease Control and Prevention criteria [26] and defined by a positive blood culture or presence of any two of the following parameters: total leucocyte count <5000/cmm; absolute neutrophil count <1500/cmm; band cell:neutrophil ratio >0.2; micro ESR >15mm at 1st hour and both of two C-reactive protein levels in specimens taken 24 h apart >1mg/dl). Heterogeneity of RRs and RDs will be estimated using interaction terms in generalized linear models of the binomial family with log and identity links, respectively.

10.2.1.f Cost effectiveness Analysis (CEA):

We will measure the incremental cost effectiveness ratio of zinc supplementation as an intervention for clinical severe infection in infants < 2 months. We will adopt an ingredient-based costing approach to value the resources used. This will involve the following steps: 1) categorize types of resources; 2) quantify # units of each resource used for each subject; 3) identify the unit cost for each resource type; 4) multiply unit costs by associated quantities and sum to generate a total cost for each subject. We will collect information on the quantity of each resource category used in 6 hourly intervals for each subject, when the study nurse records the main study data and from hospital records. Data collection will continue until

hospital discharge or death. We hypothesize that the zinc treatment will reduce the probabilities of: 1) needing ICU care; 2) needing a change in antibiotics; 3) needing closer monitoring for longer periods 4) death. Therefore, we will ensure that resource categorization is refined enough so that we can examine these potential effects (Zinc, Hospital days, ICU days, Medicines given, Procedures, Lab tests, Imaging studies, Specialist consults, Doctor time, Nurse time, Other healthcare worker time, Caregiver time, Caregiver direct costs etc.) at each monitoring point. We will develop unit prices to value resources in each category via a separate data collection effort. Valuation of resources in the healthcare context is often complicated because market prices are not readily and publically available. To address this challenge, we will use a multi-pronged approach. We will use Rashtriya Swasthya Bima Yojana (RSBY) a national health insurance scheme that maintains a public list of reimbursement rates for inpatient services. Using these and other sources (e.g., unit prices paid by the government for bulk pharmaceutical purchases), we will develop likely ranges of the economic value of each unit of each type of resource. The effectiveness of the intervention potentially involves both reductions in mortality and morbidity, which will be combined into a single metric, the quality adjusted life year (QALY). For morbidity, since children are held in hospital until they recover sufficiently, we will assume similarity between the two study arms at discharge. Therefore, we will assess quality based on the time each child spends in the regular ward (i.e., higher quality) versus the ICU (i.e., lower quality). Quality weights for these time periods will be derived from existing literature. However, because hospital stays may be shortened positively by quicker recovery or negatively via mortality and because the loss of life represents many years of lost life expectancy, we will compute the remaining quality adjusted life expectancy for each child based on current age, sex, state of residence, and residual morbidities using Sample Registration System life tables. Mean costs and mean QALYs for each study arm will be computed. If there are observable differences in baseline characteristics we will use appropriate statistical models (e.g., limited dependent variables since costs cannot be negative) to compute adjusted mean costs and QALYs. We will then compute the difference in mean costs and mean QALYs comparing the zinc arm to the standard-of-care, taking the ratio of these differences to estimate the incremental cost-effectiveness ratio (ICER) for zinc treatment.

10.2.2. Selection of Subjects to be Included in the Analysis

The compliance in our previous study was close to 100% as the study staff administered each dose of the intervention till the end of the study. The small proportion of 6% who withdrew from the study took the intervention as long as they participated in the trial. Adopting a nearly identical strategy in the proposed trial, where the study nurse will administer the intervention to the enrolled infant twice a day till discharge from the hospital, we expect to have a similarly high compliance and small loss to follow-up. Our strategy of monitoring enrolled infants at 6 hourly intervals and close interaction of the caregiver and the study staff during the hospital stay will ensure compliance

and minimize the loss to follow up and withdrawals. Close supervision by the Clinical Research Coordinator (CRC) and the PI and CoIs will reduce protocol deviations to a minimum.

At the time of discharge the parent (s) / caregiver will be advised to administer the intervention to the infant in the same dose twice a day to complete a total of 14 days. A participant booklet with a log will be provided to the parent (s) / caregiver on discharge and they will be asked to fill the log each time they administer the intervention. The parent (s) / caregiver will be asked to make a follow up visit to the hospital site 10-15 days post their discharge, after they have completed the 14 day course of intervention and asked to get the intervention bottle with them and the participant booklet. At the follow up visit data on the infant's health will be collected and a 'pill count' will be made to check the compliance.

10.2.2.a Intention-to-treat (ITT) analysis

i. All infants once found eligible and assigned an intervention and in whom the outcome is known (case fatality/ treatment failure/ discharge/ serious illness requiring hospitalization after the discharge until end of study period) will be included in the ITT analysis.

ii. Time-to-event analyses
This analysis will consider the event as death/ discharge/ treatment failure/ cessation of signs of clinical severe infection. This will be done using an intention-to-treat approach where all infants assigned into the zinc or placebo group will be analyzed. Infants where the parent/ guardian withdraws consent for continuation in the study or for other reasons cannot be followed until outcome will be censored at the time point that they were lost to follow up. Data on these lost to follow up infants till the time of censoring will be considered during the analysis. We will use a Cox proportional hazards model to compare the time to event between study groups. The hazard ratio (HR) and their corresponding 95% confidence intervals (CIs) will be calculated.

10.2.2.b Per protocol analysis

i. **Case fatality anytime from enrolment until discharge**
Infants who receive 50% or more of the projected doses in the first five days after enrolment will be included in the per protocol analysis

ii. **Treatment failure**
Infants who receive 50% or more of the projected doses in the first five days after enrolment will be included in the per protocol analysis

iii. **Death at any time after discharge from hospital until end of study period, which is 12 weeks after the day of enrolment**
Infants who receive 50% or more of the projected doses in the 14 days after enrolment will be included in the per protocol analysis

iv. **Severe illness requiring hospitalisation at any time after discharge from hospital until end of study period**

Infants who receive 50% or more of the projected doses in the 14 days after enrolment will be included in the per protocol analysis

11. DIRECT ACCESS TO SOURCE DATA/ DOCUMENTS

Trial-related monitoring, audits, IEC review, and regulatory inspection(s) will be permitted by providing direct access to source data/documents to sponsors or monitors and regulators.

12. QUALITY CONTROL AND QUALITY ASSURANCE

Misclassification will be minimized by employing strict definitions of the outcomes, developing clear and concise Standard Operating Procedures (SOPs), initial training before study initiation and repeated training during study implementation, as well as regular and rigid supervision of study staff.

Quality control will be implemented right from the recruitment stage where we will follow very stringent criteria for recruiting staff for the research related activities. We will ensure that the personnel recruited for the study have the desired qualifications and experience to take up their responsibilities. All levels of the staff will receive training consistent with the Good Clinical Practices guidelines (GCP) and in the study protocol. The staff will then be trained in the study protocol specific activities as per GCP. The clinical research coordinator (CRC) will ensure adherence to implementation of the strategy and along with the research officers (RO) will ensure quality control through regular checks on all activities being performed in the study, such as screening of infants aged 1-59 days, coming to the hospital out patients department (OPD) or emergency department, taking written consent from parents/ guardians, enrolment of eligible infants, administering the intervention/ placebo at enrolment and then every 12 hourly until discharge and advising the parents/ caregiver to continue with the intervention at home after discharge in the same dose to complete a total of 14 days, follow up of enrolled infants every 6 hourly, assessing for and documenting outcomes, management of serious adverse events, collection, immediate processing and storage of blood samples at the designated time points, calibration of clinical and labortatory equipment like infant weighing scale, infantometer, incubator, centrifuge, refrigerator, deep freezers, pipettes, etc.

The CRC will assist the investigators in developing clear and concise case report forms (CRF), responsibility logs, equipment calibration logs, training logs, and standard operating procedures (SOP) for each research related activity like screening with clear objective definitions for determining eligibility, taking informed consent, enrolment, administering the intervention/placebo, follow up, clear objective definitions for assessing outcomes, documenting and management of serious adverse events, reporting of SAEs following the regulatory guidelines, form filling guidelines, calibration of equipment, laboratory procedures like collection of blood, immediate processing, transportation and storage of samples.

Initial training and then repeated training during study implementation will be provided to the study staff on each relevant study activities. In order to ensure that the standardized protocol is followed at all sites, standardization exercises will be done within each site and then between different sites at regular defined intervals.

In addition, the following QC/QA procedures will be ensured:

- Maintaining a paper trail whenever there are changes in the CRFs and the SOPs
- Proper storage of the consent forms under lock and key with restricted access.
- A systematic registration of all relevant baseline characteristics will enable us to adjust for relevant confounders, should variables associated with the outcomes be unevenly distributed between trial arms by randomization.

  Quality control (QC) of lab samples will be done using a standard protocol

12.1. Staff Training

The components of the study specific training are:

- Protocol and therapeutic area training
- Training in CRF filling guidelines
- Class room training for determining eligibility, taking informed consent from parents/ guardian, enrolling infant, assigning intervention, administering intervention, ascertainment of outcomes, adverse events, reporting of adverse events, blood sampling, naso-pharyngeal aspiration, calibration of site equipment, documentation practices.
- Role plays for obtaining written informed consent
- Training for all possible emergency scenarios by group discussions and mock exercises

12.2. Site Monitoring

Site readiness monitoring visit: After appropriate ethics approvals by all the relevant Institutional Ethics Committees (IECs) are available (and the final protocol has been amended as required by IECs), a **'site initiation visit'** will be conducted before the first participant is enrolled in the study. The participants cannot be enrolled until occurrence of such visit and its documentation. During this site visit, the requirements of protocol procedures, and all logistical issues will be discussed at length. During the visit, the monitor will complete a standard checklist that covers key aspects to indicate the readiness of the site. When the monitor has shared site readiness report with the PMT, the PI or the PMT member designated by the PI will together with the monitor develop a plan of action to address any problems identified during the visit. The PI is responsible for the implementation of the plan and addressing all problems before recruitment of the first study participant.

Regular monitoring and audit will be conducted at the hospital sites and the coordinating centre by external agencies. After the study is initiated, the study monitor will be in regular contact with the sites to obtain information on the performance of the study. These contacts will be scheduled to take place at **regular intervals**. Subsequent to start of recruitment, **routine-monitoring visits** would occur after prior appointment with the investigators.

The investigator and his/her staff are obliged to devote a suitable amount of time and an appropriate place for the monitoring visits. Monitoring visits will include, but will not be limited to, review of files, intervention accountability records, case report forms, informed consent forms, medical and laboratory reports, and protocol compliance. During each visit, the monitor will review the Case Report Form (CRF) of

each participant in the study with regard to completeness, thoroughness and compliance with the protocol. CRFs will be filled up for all the participants who receive study intervention by study staff. For those participants who are dropped before the completion of the study, the reason for their termination must be specified. Such reports must be signed by the site Investigator or his/her designee and submitted to the PMT in the same manner as completed reports.

In addition, at a minimum, the original participant data will be reviewed to ensure that:

- participant informed consent is incorporated;
- inclusion/exclusion criteria are properly followed;
- CRF data are consistent
- all relevant clinical and laboratory findings and concomitant medication are documented in the CRFs;
- quantity and dosing schedule of concomitant medication is documented in the CRFs;
- quantity and dosing schedule of the Investigational/Comparator Product is in accordance with the protocol;
- all relevant information (e.g., any adverse event) has been recorded in the appropriate place in the CRFs;
- All AEs and SAEs have been managed as per standard case management
- the Investigational/Comparator Product is being stored correctly, and its supply is being properly accounted for;
- Incorrect or illegible entries in the CRFs would be submitted to the site investigator or his/her designee for correction.

The monitor will retrieve completed CRFs during the regularly held monitoring visits. The study monitors will meet with site investigator or his/her designee to discuss any problems and actions to be taken, and document visit findings and discussions. When the monitor has shared 'monitoring visit' report with the PMT, the PI or the PMT member designated by the PI will together with the monitor and the site investigator/ designee develop a plan of action to address any weaknesses/challenges identified during the visit. During the study period, the responsible CDSA staff will be available to answer questions with regard to the performance of the study.

12.3. Auditing

In addition to the above outlined monitoring visits, the participating institutions may be audited. This audit may be carried out by representatives of monitoring agency or by the responsible regulatory authority (ies). Such an audit would be done to review whether the data has been properly recorded in the interim or final report and whether the performance of the study is in accordance with the protocol, and other relevant guidelines. Participant confidentiality will be maintained at all times. The investigator will inform monitoring agency immediately if an audit has been requested by a regulatory authority/committee /agency.

13. ETHICS COMMITTEE CLEARANCES

The investigator will initiate the study only after securing approval from the Ethics Committees of all the seven hospital sites in which the study is to be carried out, THSTI IEC, NHRC Nepal and REC West in Norway. Appropriate written informed consent will be obtained from the parents or guardians of each participant after explaining the nature of the study, expected benefits and possible adverse effects of the study intervention as explained in the Participant Information Sheet & Informed Consent Form.

Participant confidentiality will be maintained at all times during the conduct of the study, and after completion of the study.

India: Approvals will be obtained from the institutional ethics committees of THSTI and the hospital sites.

Nepal: approvals will be obtained from National Health Research Council (NHRC) and the ethics committees of the hospital sites.

14. CONSENT FOR PARTICIPATION

Informed written consent will be taken from one or both parent(s)/ guardian of the participating infant before screening and enrolment into the study. The study nurse taking the informed consent will speak to the parent(sj)/ guardian in the language they understand. She/ he will provide the parent (s)/ guardian with the information sheet that will be in the local language and will detail the focus of the study along with the associated risks and benefits of the trial. The study nurse will first explain to the parents/ guardian verbally about the study before giving them the information sheet. She/ he will explain why the study is being done, how it is being done. She/ he will emphasize that the infant will continue to get the standard care (standard antimicrobials and other supportive care) that he/she should get for his/ her illness.

If the parents/ guardians agree for participation in the study then in addition to the standard antimicrobials the infant will get either zinc or placebo as the intervention. The only reason for giving a placebo, which is a totally harmless product, is that it will help in knowing whether zinc is effective in treating the serious infection that their baby has; if those who get zinc fare better then it means that zinc is effective as an adjunct to the standard treatment for this disease/infection. The parent(s)/ guardian will be explained that neither the doctor nor the parent(s)/ guardian will know whether zinc or placebo is being given; this is done so that no one is influenced by this foreknowledge and standard high quality care is provided to all babies.

They will also be explained that a small quantity of blood will be taken for routine investigations for standard management and some specific markers of the infection and immune status and for zinc estimation. It will be emphasized that their baby will be constantly monitored in the Pediatric ward and all cost of the medication will be taken care of by the study. They will be informed that the infant will be kept in the hospital till complete recovery and will get all medications from the hospital/ study.

It will also be emphasized that there is enough evidence to show that zinc is a very safe micronutrient. It will be explained to the parent(s)/ guardian that participation in the study is **voluntary** and even if they agree for participation initially they are free to

leave the study whenever they desire. This decision will in no way affect the standard care that they receive at the hospital.

Once the information sheet is provided to the parent(s)/ guardian they will read it themselves. Illiterate parents will be encouraged to bring along a literate neighbour or relative as a witness. The parent(s)/ guardian will be given an opportunity to ask any questions that they have, which will be answered by the person administering consent or the study supervisors. The parents will be asked a set of questions to assess their understanding of the consenting procedure and the study. The consent process would be repeated if the parent(s)/ guardian have not understood clearly.

Once the person administering the consent is satisfied that the parent(s)/ guardian have understood the consent form and are willing for participation in the study if found eligible, she/he will ask the parent(s)/ guardian to sign (or put a thumb impression in case illiterate) the consent form. If the parent(s)/ guardian are illiterate in addition to the thumb impression, a literate impartial witness will be asked to sign the consent form.

If the consent is refused, the infant will not be included and the cause for not including will be recorded. The parent(s)/ guardian will be assured that all services and treatment that the infant will receive at the hospital will continue and nothing will change even if they choose not to participate in the study.

The filled consent forms will be filed and locked in a cabinet at the hospital site. A duplicate original signed copy of the consent form will be given to the parents. The participant's contact information will be kept in a locked cupboard at the hospital site while the study is ongoing and shifted to a locked cupboard holding the study documents at the coordinating centre once the study is completed.

Serum specimens of those participants whose parent(s) have refused for analysis of the samples for research purpose (after blood sampling was done)

The laboratory technician in the presence of the CRC will discard all aliquots of the serum specimens of these participants. A list of these subject IDs will be typed, dated, initialed by those who witnessed the procedure and noted in the laboratory file.

Participant confidentiality

Any information obtained about the enrolled infant in the course of the study constitutes confidential information. This information will be used solely for the purposes of the trial and will not be shared with anyone that is not connected with the study. All the clinical and laboratory data collected from the enrolled infants will be coded with a unique serial number. Only the study staff will know what this number is. All research records will have very restricted access. In the event of any publication or presentation resulting from the study, no personally identifiable information will be shared.

All records with the participant identification information will be kept in a locked cupboard in the coordinating centre and will be accessible to only the investigators.

All CRFs will only have participant IDs, participant initials, age and no other identifiers. Data will be provided only to the DSMB, the IECs, auditors and the funding agency, if deemed necessary.

Incentive for participation

The principal and site investigators will ensure that no verbal or financial incentive is used to coerce families to participate.

15. DATA HANDLING AND RECORD KEEPING

15.1. Data Management

The data will be managed by the Data Management Centre (DMC) at THSTI.

i. **Description of data arising from proposed research:** The data will consist of baseline information on clinical and demographic characteristics of infants screened and enrolled in the study and details of 6 hourly progress including intervention, co-interventions, progress of clinical illness resulting in recovery, treatment failure or death during the hospitalization.

ii. **Data collection, including type, format, scale and standards for data:** All the data will be collected in standard forms electronically at the seven sites by the research nurses/ medical officers supervised by the study physician (research officer). The forms will be developed by the DMC and will consist of details of all the variables with standard definitions and labels. Consistency and range checks will be inbuilt into the “data entry screens” such that these checks are applied during entry itself. Each form will be transferred to the server at the DMC daily. The DMC will train the study staff for data entry, running ranges and consistency checks, and responding to errors. The DMC will generate query forms which will be returned to the hospital research staff for concurrent verification daily.

iii. **Data security and storage:** At least 3 backups of the data will be kept in the server at the DMC protected by a specific password and accessible to only authorized users. DMC will send the data every month to a central data repository which will be established at THSTI.

iv. **Data tracking, cleaning, and quality checks:** DMC will create forms for data tracking and information on the subjects screened and enrolled will be sent to all the investigators the first week of every month. The DMC will be responsible for initial cleaning of the data. Interim tabulations and scatter plots for some variables will be made at regular intervals to identify data errors. Special checks will be made on observations that are more than two or three standard deviations from the mean. There will be a regular feedback of errors from the DMC to the clinical sites.

v. **Feedback to Data Safety Monitoring Board (DSMB):** A feedback form for the DSMB containing relevant information will be prepared by the data management

team in consultation with the project management team and the site investigators and sent to the DSMB at regular intervals.

15.2. Record Retention and Archival

Essential documents, including administrative documentation, as well as documentation relating to each infant screened or enrolled in the study, including informed consent, locator information, CRF's, and all source documents, should be retained until at least 5 years after study is completed and the results have been published. All records must be retained at the site throughout the study.

All the study documents including participant's source data and documents will be archived by the study sites after the completion of the study, till the time the sponsor informs in writing to the study sites that they no longer need to maintain the study documents.

16. STUDY ORGANIZATION

16.1. Governance

16.1.1. Technical Steering Committee (TSC)

A Technical Steering Committee (TSC) will be established by the funding agency comprising of both national and international experts. A distinguished and renowned scientist will chair the Committee. The members will be from all representative domains such as epidemiology, neonatology, pediatrics and immunology. Additional domain experts will be consulted as and when required.

Specific Roles and Responsibilities:

- review scientific data generated from the study and provide regular feedback to the investigators and the funding agency
- create a measurement matrix for specific outcomes and review if the original goals are being met
- give new directions for research

The committee will meet twice a year. Meetings with individual Technical Steering Committee members will take place when and if required by the Chair or the funding agency.

16.1.2. Project Management Team (PMT)

The PMT will comprise of the Principal Investigators from PBC, THSTI, Tribhuvan University, Nepal and CIH, Bergen, Norway and lead investigators from all partnering hospital sites. It is responsible for the overall governance of the study. The PI from THSTI is responsible for coordinating the PMT, which will meet (in person or on skype) every month (or earlier if required). Subject experts will be invited as and when required to give their technical advice. The Clinical Research Coordinator (CRC) from PBC will be the

primary link between the SCU and the PMT. The SCU will report to the PMT regarding study progress and updates every month. The PMT will review the conduct of the study, which will include visits to the hospital sites, the scientific and other technical aspects.

The final study protocol, management plans, SOPs, logs and the data recording tools with essential site adaptations will be approved by the PMT before the implementation of the protocol.

Specific Roles and Responsibilities:

- overall governance of the study
- to provide a consultative forum that can effectively advice on current and future directions for day to day management of the study.
- to provide advice and support on the planning, implementation, monitoring and reporting of the study
- to review the conduct of the study on the scientific and other technical aspects.

16.1.3. Study Coordination Unit (SCU)

This will comprise of the Clinical Research Coordinator (CRC), Senior Research Officer (SRO), Research Officers (RO)/ Supervisors from each hospital site. The SCU will be responsible for the overall site coordination. It will be responsible for the implementation of the final study protocol. The CRC will be responsible for convening and functioning of the SCU. The SCU will meet (in person or on skype) every 15 days (or earlier if required) to review the management and the progress of the study.

16.2. Study Organization

16.2.1. Responsibility of the site investigators (consultant neonatologists/pediatricians) at the hospital sites

The consultant neonatologists/pediatricians (site investigators) will be responsible for providing all the clinical care to the enrolled participants. They will provide the clinical care to the infants as per standard of care protocol. Regular orientation seminars will be done by the study team for the hospital clinical staff at these hospital sites so that they are aware of the clinical trial being done at their hospital, can identify the infants enrolled in the study from the enrolment ID number and can facilitate the use of the approved protocol for providing standard care to the sick infants.

16.2.2. Study Clinical Team

The study clinical team will comprise of the Clinical Research Co-ordinator (CRC), Senior Research Officer (SRO), Research Officers (RO), Study Nurses (SN)/ Medical Officers (MO), and Field Technicians (FT)/ Study Assistants. **The study clinical team comprised of Study Nurses/ Medical Officer, and Field Technicians/ Study Assistant will be on duty round**

the clock and will be available at each hospital site at all times of the day on a rota system. Broad guidelines of the responsibilities of this team are given below.

i. **Clinical Research Coordinator (CRC)**

The CRC will be leading this team and will be primary point of contact for all study management related aspects. The CRC will have an oversight responsibility for activities undertaken by SCU. He/she will be responsible for:

a) Developing training module and planning the initial and retraining sessions for the research study staff along with the SRO and the ROs.
b) Ensuring compliance with the project requirements and cascading the updates to respective team
c) Contribute through operational inputs in protocol and study budget related decisions
d) Supporting the submissions for relevant government / ethical approvals.
e) Structuring and supervising compliance for the study management plans.
f) Supervising the site ROs for the site preparation, monitoring, and ongoing quality management.
g) Supervising the data management progress with data manager
h) Planning, tracking and management of study milestones
i) Keeping stakeholders informed on study progress, risks and accomplishments

ii. Senior Research Officer (SRO)

The SRO will coordinate the study activities at the hospital sites. He/she will be responsible for the efficient management of clinical and laboratory activities of the study and will support the site PIs and the hospital clinical staff providing services/training to the study clinical team.

iii. Research Officer

The ROs will supervise the activities of the SN/ MO and the FT/SA and will be responsible for:

a) supervision of process of taking written informed consent
b) supervision of ensuring eligibility of infants before they are enrolled in study
c) supervision of outcome assessment
d) final review of the CRF and the signing of the CRF (as a part of QC)
e) tracking the reports of all the laboratory samples collected for standard clinical care for ‘clinical severe infection’
f) tracking the collection, immediate processing, temporary storage and transportation of bio specimens collected for the research study

iv. Study nurses (SN)/ Medical officer (MO):

The SN/ MO will be responsible for:

a) taking written informed consent
b) screening ill infants aged 1d-59d who come to the emergency rooms of the hospital sites, evaluating eligibility criteria and enrolling the infants
c) collecting data on all the relevant clinical examination
d) completing the case recording forms (CRF)
e) collecting biospecimens for the study at the pre-specified time points

v. **Field technician (FT)/ Study Assistant (SA)**

The FT/ SA will be specifically responsible for:

a) assisting the study nurse/ medical officer in administering the intervention to the enrolled infant
b) assisting the SN/MO in collecting bio specimens of the enrolled infant
c) immediate processing and temporary storage of collected bio specimens
d) maintaining laboratory records in the CRF.
e) transportation of all laboratory samples to hospital site side lab or to the central storage facility at THSTI (India)/ IOM (Nepal) as relevant
f) maintaining detailed input-output charts of the enrolled infants
g) making a home visit to the enrolled infant's house for the post discharge data collection if the need arises
h) making reminder calls to the enrolled infant for scheduled follow up visits in the pediatric OPD

17. PROPOSED TIME LINE OF STUDY ACTIVITIES

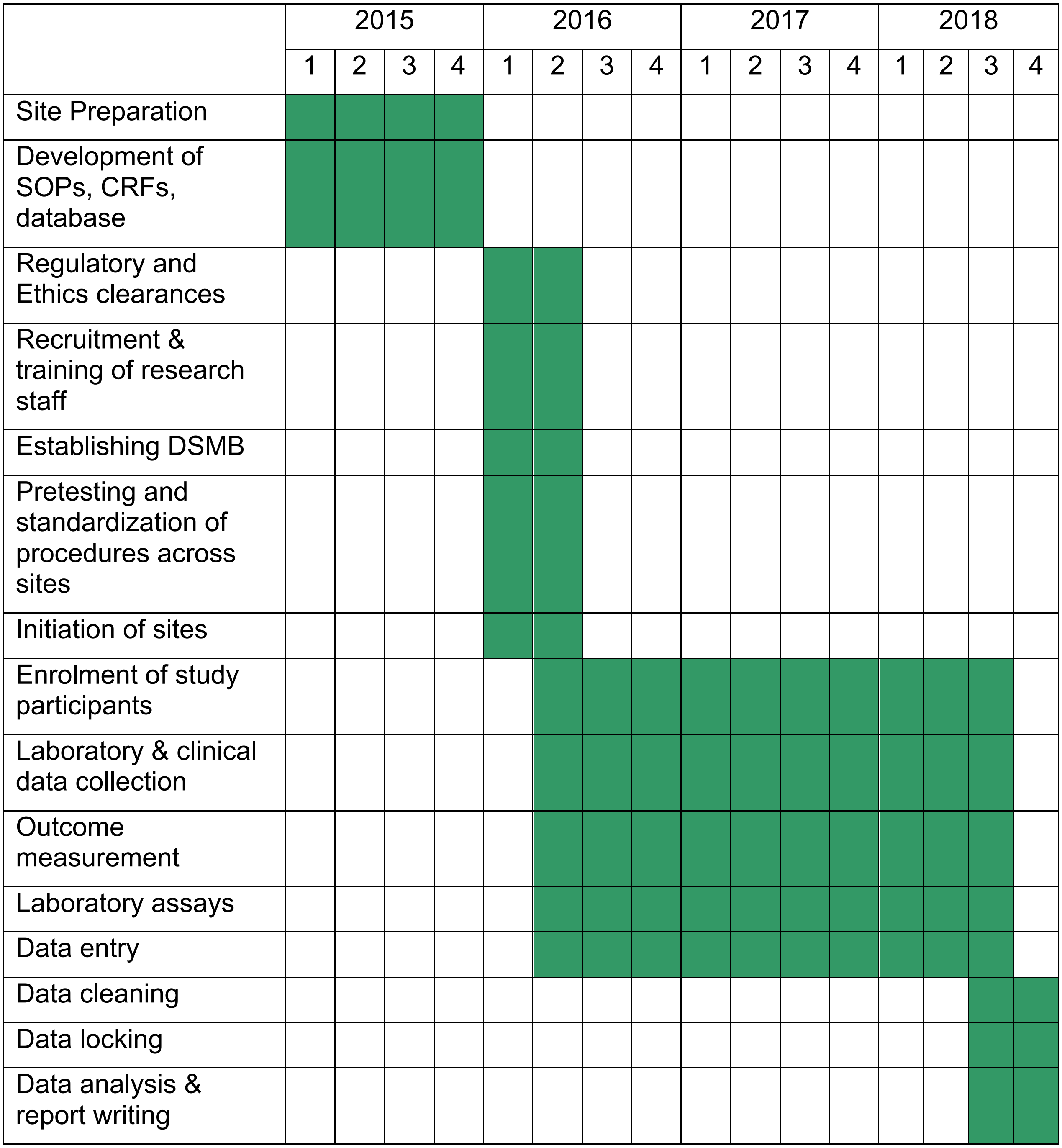

Figure 2: Timeline of the study

18. CONTRIBUTION OF EACH ORGANIZATION

All the collaborating institutes in the study have agreed to assume responsibilities that are largely non-overlapping, but with regular cross-talk among themselves for implementation of the study design, exchange of information, data and ideas for an efficient conduct of the study.

18.1. THSTI (PBC), SJH, MAMC, CNBC, KH

PBC, THSTI will be the main coordinator of the study. The implementation of the study will be coordinated by the Pediatric Biology Centre (PBC) at the Translational Health Science and Technology Institute (THSTI), in the National Capital Region (NCR), Delhi. SJH, MAMC, CNBC and KH will be the hospital sites in India for recruitment of eligible infants. During the preparation phase of the study and development of SOPs and other logistical steps, THSTI will work closely with the collaborating institutions and the 7 hospital sites. The laboratory assays for the immunobiological readouts of the samples from the Indian sites will be analyzed at the central laboratory at THSTI

CDSA is an extra mural centre of THSTI**.** It will be responsible for the quality management of the study.

18.2. Tribhuvan University, Institute of Medicine, Nepal

IOM will be the coordinator of the study of the Nepal hospital sites. IOM, PH and KCH will be the hospital sites in Nepal for recruitment of eligible infants. During the preparation phase of the study and development of SOPs and other logistical steps, THSTI will work closely with the IOM and the 7 hospital sites. The laboratory assays for the immunobiological readouts of the samples from the Nepal sites will be analyzed at a central laboratory at IOM.

18.3. Centre for International Health, University of Bergen, Norway (CHN-CIH)

CHN-CIH will play an advisory role and will help in establishing the 'Data Management Centre' for the study at THSTI and in data analysis.

18.4. Centres for Health Policy/Primary Care and Outcomes Research at Stanford University School of Medicine (CHP/PCOR)

CHP/PCOR will collaborate with THSTI to measure the incremental cost effectiveness ratio of zinc supplementation as an intervention for clinical severe infection in infants <2 months.

18.5. Radbound University, Nijmegen, Netherlands

Radbound University will collaborate with THSTI and Institute of Medicine Tribhuvan University for the immunological studies to explain potential mechanisms

19. FINANCING AND BUDGET

This study will be executed as a bilateral collaborative project **under the 'Program of Cooperation' between the Department of Biotechnology**, Indian Institutions (Translational Health Science and Technology Institute, Faridabad; Regional Centre

for Biotechnology, Faridabad; Society for Applied Studies, New Delhi; Christian Medical College, Vellore) **and the Norwegian Institutions (University of Bergen, Norway** and Norwegian Institute of Life Sciences, Norway) **under the agreement in Science and Technology between Government of Republic of India and Government of Kingdom of Norway** (Copy of approval from DBT for executing the study under the Program of Cooperation enclosed as **Annexure II**)

Budget: This study has been granted funding by Research Council of Norway under the Research Grant on Global Health and Vaccination Research (GLOBVAC) (18.02 million Norwegian Kroners) and CISMAC (Centre for Intervention Sciences in Maternal & Child Health), Norway (6.5 million Norwegian Kroners)
The study will be implemented at seven hospital sites, 4 in New Delhi, India and 3 in Kathmandu, Nepal. For easy logistics, funding for the Indian sites and CIH will come from GLOBVAC and funding for the Nepal sites will come from CISMAC. A site wise budget has been prepared for the GLOBVAC funds allocated for the Indian sites. The personnel who will conduct the study at the 4 hospital sites will be employed by Translational Health Science and Technology Institute (THSTI) but deployed at the respective hospitals. There will be no financial transfer of budget to the hospital sites. (Site wise budget enclosed as **Annexure III**)

20. PUBLICATION POLICY

The results of the study will be written up as a report for submission, presentation, or publication. The report will be used for regulatory submissions, presentation at a meeting, or publication in a journal. All publications emanating from this trial will be reviewed by the participating institutions. Authors will be determined based on actual input into the publications, according to existing guidelines.
The report will be authored and approved by the Investigators. Before finalizing it, however, stakeholders will review the report and offer comments. The authors of the study report will be the Investigators conducting the study and the Experts/Advisers to the study.

Following documents are provided as Annexures of the clinical study protocol

- **Annexure I: Circular (File No. 12-01/14-DC (pt. 47)) from the DCGI dated 10-11-2015**
- **Annexure II: Approval from DBT for executing the study under the ‘Program of Cooperation'.**
- **Annexure III: Site wise budget**
- **Annexure IV: Informed Consent Document (English and Hindi)**

21. REFERENCES

1. Liu L, Johnson HL, Cousens S, Perin J, et al. Global, regional, and national causes of child mortality: an updated systematic analysis for 2010 with time trends since 2000. Lancet 2012;379(9832):2151-61.
2. Black RE, Cousens S, Johnson HL, et al. Global, regional, and national causes of child mortality in 2008: a systematic analysis. *Lancet* 2010; **375**:1969–87.
3. Lawn JE, Cousens S, Zupan J. 4 million neonatal deaths: when? Where? Why? *Lancet* 2005:**365**;891–900.
4. Young Infants Clinical Signs Study Group. Clinical signs that predict severe illness in children under age 2 months: a multicentre study. *Lancet* 2008; **371**:135–42.
5. The WHO Young Infants Study Group. Bacterial etiology of serious infections in young infants in developing countries: results of a multi-center study. *Pediatr Infect Dis J* 1999; **18**:S17–22.
6. Bassani, DG, Kumar R, Awasthi S. *et al.* Causes of neonatal and child mortality in India: a nationally representative mortality survey. *Lancet* 2010:**376**;1853–1860.
7. Annual Report Department of Health Services (2009-2010) Government of Nepal Accessed on 29th February, 2012 at http://mwrhd.gov.np/annualreports.html
8. Bhatnagar S, Wadhwa N, Aneja S et al. Zinc as adjunct treatment in infants aged between 7 and 120 days with probable serious bacterial infection: a randomised, double-blind, placebo-controlled trial. Lancet 2012; 379:2072-8.
9. Shankar AH, Prasad AS. Zinc and immune function: the biological basis of altered resistance to infection. *Am J Clin Nutr* 1998; **68**:447S–63S
10. Prasad AS. Zinc and immunity. *Mol Cell Biochem* 1998; **188**:63–9.
11. Sazawal S, Jalla S, Mazumder S, Sinha A, Black RE, Bhan MK. Effect of zinc supplementation on cell-mediated immunity and lymphocyte subsets in preschool children. *Indian Pediatr* 1997; **34**:589–97.
12. Bhatnagar S, Bahl R, Sharma PK, Kumar GT, Saxena SK, Bhan MK. Zinc with oral rehydration therapy reduces stool output and duration of diarrhea in hospitalized children: a randomized controlled trial. *J Pediatr Gastroenterol Nutr* 2004; **38**:34–40.
13. Lazzerini M, Ronfani L. Oral zinc for treating diarrhoea in children. *Cochrane Database Syst Rev* 2008; **3**:CD005436.
14. Lukacik M, Thomas RL, Aranda JV. A meta-analysis of the effects of oral zinc in the treatment of acute and persistent diarrhea. *Pediatrics* 2008; **121**:326–36.
15. Bhutta ZA, Black RE, Brown KH, et al. Prevention of diarrhea and pneumonia by zinc supplementation in children in developing countries: pooled analysis of randomized controlled trials. Zinc Investigators' Collaborative Group. *J Pediatr* 1999; **135**:689–97.

16. Sandstead HH. Zinc deficiency. A public health problem? *American Journal of Diseases of Children*, 1991, 145:853–859.

17. Zinc. In: *Trace elements in human nutrition and health*. Geneva, World Health Organization, 1996.

18. Prasad AS et al. Biochemical studies on dwarfism, hypogonadism and anemia. *Archives of Internal Medicine*, 1963, 111:407–428.

19. Brown KH et al. Assessment of the risk of zinc deficiency in populations. *Food and Nutrition Bulletin*, 2004, 25:S130–S162.

20. World health report 2002: reducing risks, promoting healthy life. Geneva, World Health Organization, 2002.

21. Brown KH et al. Overview of zinc nutrition. *Food and Nutrition Bulletin*, 2004, 25:S99–S129.

22. Hotz C, Brown KH. Identifying populations at risk of zinc deficiency: the use of supplementation trials. *Nutrition Reviews*, 2001, 59:80–84.

23. Castillo-Duran C, Vial P, Uauy R. Trace mineral balance during acute diarrhoea in infants. *Journal of Pediatrics*, 1988, 113:452–457.

24. Leonard A, Gerber GB, Leonard F. Mutagenicity, carcinogenicity and teratogenicity of zinc. *Mutation Research*, 1986, 168:343–353.

25. Vallee BL, Falchuk KH. The biochemical basis of zinc physiology. *Physiological Reviews*, 1993, 73:79–118.

26. Samman S. Trace elements. In: Mann J, Truswell S, eds. *Essentials of human nutrition*, 2nd ed. New York, Oxford University Press, 2002.

27. Festa MD et al. Effect of zinc intake on copper excretion and retention in men. *American Journal of Clinical Nutrition*, 1985, 41:285–292.

28. Clinical management of acute diarrhoea: WHO/UNICEF joint statement. Geneva, World Health Organization, 2004.

29. Strand TA et al. Effectiveness and efficacy of zinc for the treatment of acute diarrhoea in young children. *Pediatrics*, 2002, 109:898–903.

30. Bahl R et al. Efficacy of zinc-fortified oral rehydration solution in 6- to 35-month-old children with acute diarrhoea. *Journal of Pediatrics*, 2002, 141:677–682.

31. Penny ME et al. Randomized, community-based trial of the effect of zinc supplementation, with and without other micronutrients, on the duration of persistent childhood diarrhoea in Lima, Peru. *Journal of Pediatrics*, 1999, 135:208–217.

32. Sazawal S et al. Zinc supplementation for four months does not affect plasma copper concentration in infants. *Acta Paediatrica*, 2004, 93:599–602.

33. Bhatnagar S et al. Zinc with oral rehydration therapy reduces stool output and duration of diarrhoea in hospitalized children: a randomized controlled trial. *Journal of Pediatric Gastroenterology and Nutrition*, 2004, 38:34–40.

34. Bhutta ZA, Nizami SQ, Isani Z. Zinc supplementation in malnourished children with persistent diarrhea in Pakistan. *Pediatrics*, 1999, 103:e42.

35. Sazawal S et al. Zinc supplementation in infants born small for gestational age reduces mortality: a prospective, randomized, controlled trial. *Pediatrics*, 2001, 108:1280–1286.

36. Osendarp SJ, West CE, Black RE. The need for maternal zinc supplementation in developing countries. *Journal of Nutrition*, 2003, 133:817S–827S.

37. Sazawal S et al. Effect of zinc supplementation on mortality in children aged 1–48 months: a community-based randomised placebo-controlled trial. *Lancet*, 2007, 369:927–934.

38. Bhandari N et al. The effectiveness of zinc supplementation plus ORS compared to ORS alone as a treatment for acute diarrhoea in a primary health care setting: a cluster randomized trial. *Pediatrics* (in press).

39. Integrated management of childhood illness. World Health Organization, Geneva & UNICEF 2008. Accessed on 29th February, 2012 at http://www.who.int/child_adolescent_health/documents/IMCI_chartbooklet/en/index.html

40. Physicians chart booklet. Integrated Management of Neonatal and Childhood Illness. World Health Organization, Geneva, UNICEF & Ministry of Health & Family Welfare Govt. of India, India: 2003. Accessed on 29th February, 2012 at http://mohfw.nic.in/NRHM/IMNCI/IMNCI_index.htm

Brief Statistical Analysis Plan for Zinc as an adjunct for the treatment of clinical severe infection in infants younger than 2 months (doi: 10.1186/s40360-017-0162-5)

Main research question: Does oral zinc used as an adjunct to standard treatment reduce risk of death in infants with clinical severe infection?

Hypothesis: Daily oral administration of 10 mg elemental zinc as an adjunct to standard therapy to infants aged 3 to 59 days hospitalized for clinical severe infection (CSI) will result in a relative mortality risk reduction (i.e. efficacy) of at least 30% both during hospitalization (case fatality) as well as for the period up to 12 weeks from the day of enrolment (extended case fatality).

Primary objectives: Estimate the efficacy of the adjunct zinc treatment for CSI against

1. case fatality
2. extended case fatality

Secondary objectives: Estimate the efficacy of the adjunct zinc treatment for CSI:

i. against treatment failure during initial hospitalization, i.e. required life support (ventilation or vasoactive drugs) or need to change antibiotics because of persistence of CSI signs after 48h of enrolment, or worsening of existing or appearance of new CSI signs or death
ii. against death at any time after discharge from hospital until 12 completed weeks from enrolment
iii. against severe illness requiring hospitalisation at any time after discharge from hospital until 12 completed weeks from enrolment.
iv. with respect to time to cessation of CSI signs (beginning of a 48-hour period with no CSI signs)
v. with respect to time to discharge from hospital

We will compare the risk of case fatality [primary objective 1 (1°#1)], extended case fatality (1°#2) and treatment failure (2°#i), death from discharge to 12 completed follow-up weeks (2°#ii), and of rehospitalization for severe illness (2°#iii) between the two trial arms to arrive at estimates of relative risk (RR); efficacy (1-RR), risk difference (RD) and number needed-to-treat (NNT=1/RD). As a supplementary analysis, we will also estimate the time to death from enrollment while in hospital (1°#1) and to the end of 12 weeks after enrollment (1°#2). Such time-to-event analysis will also be used to estimate the efficacy of adjunct zinc therapy with respect to time to cessation of CSI signs (2°#iv) as well as to hospital discharge (2°#v).

Graphic presentations and analytic procedures

We will in the trial profile present all screened patients down to those randomized and whose data were analyzed. The baseline table will present the characteristics of the enrolled participants by trial arm. The information will be used to evaluate whether any baseline differences of prognostic factors should be adjusted for (confounding) and depict the type of CSI patients the study findings pertain to (external validity). We will in the supplement also present a baseline table for all randomized patients separately for the 240 patients enrolled in the two hospitals in Nepal and the 2913 patients enrolled in the 5 Indian hospitals. The baseline tables will include but will not necessarily be limited to the following characteristics:

<u>Mother and patient's family:</u>
Mother's education (years)
[Any other maternal/family characteristics]

Patient:

- Age
  - Mean (SD) age
  - Categorized in 3-6 days, 7-28 days and 29-59 days)
- Sex
- Weight in kg (Mean [SD])
- Weight for age Z scores (< -2 Z-score) (n[%])
- History
  - Antimicrobial therapy for current illness received before admission
- Clinical signs
  - Axillary temperature $\geq$ 38°C
  - Stopped feeding well
  - Severe chest in-drawing
  - Grunting
  - Fast breathing (i.e. respiratory rate $\geq$60/min)
  - Convulsions
  - Movement only when stimulated
  - Refusal to feed
  - Diarrhea
- Laboratory parameters
  - Hemoglobin (g/dL)
  - Total Leukocyte Count (cells/μL)
  - Absolute Neutrophil Count (cells/μL)
  - Band cell: Neutrophil ratio
  - Micro-ESR(mm)
  - CRP (mg/L)
  - Plasma zinc concentration (μg/dL) median (IQR)

Statistical Analysis

Using generalized linear models of the binomial family with log and identity links we will estimate the RRs (and corresponding efficacies) and RDs (and corresponding NNT), respectively, for the two primary objectives (death during hospitalization and death until 12 completed weeks after enrolment) as well as for the secondary objectives i (treatment failure), ii (death from discharge until 12 completed weeks after enrolment) and iii (post-discharge rehospitalization for severe illness). Treatment failure will be reported both by (i) first event and by (ii) worst event.

In the supplementary analysis, we will estimate the corresponding hazard ratios for time to death from enrolment while in hospital (1°#1) and to the end of 12 weeks after enrolment (1°#2), right-censoring children who die on the day of their demise. Patients will also be censored when the caretaker withdraws consent for study continuation or the infant for other reasons could not be followed up for as long as we intended.

Such supplementary time-to-event analysis will also be used to estimate the efficacy of adjunct zinc therapy with respect to time to death at any time after discharge from hospital until 12 completed weeks from enrolment (2°#ii), time to cessation of CSI signs (2°#iv), and time to hospital discharge (2°#v).

We will also use Poisson regression or negative binomial regression, to address secondary objectives.

Zinc may exert an effect only after some time. In an exploratory analysis, the above analyses will therefore be repeated where the outcomes are redefined to occur only when they take place after 24 h of administering the first dose of zinc.

Subgroup analyses
We will estimate the efficacy of zinc in various subgroups based on:
Parameters on which the randomization was stratified:

A. presence or absence of diarrhea on admission,
B. study hospital and country

Other parameters:

C. Age at enrolment (3-6 days vs. ≥ 7 days)
D. Positive vs. negative septic screen[1]

We will display the RDs for each of the above-mentioned strata and depict them in a forest plot. We will also estimate the heterogeneity of RDs using interaction terms in our regression models to determine the absolute excess risk due to interaction.

Our primary analysis will use an intention-to-treat approach where all infants assigned into the zinc or placebo arm of the trial and whose outcome is known will be included. We will also consider Instrumental Variable Analyses (IVA) attempting to shed light on what may be a better estimate of the intrinsic efficacy of adjunct zinc therapy for CSI, i.e. the efficacy of zinc had it been given to all children in the scheduled doses and intervals. In our IVA, the random allocation will be the instrument and actual amount of zinc administered to each baby over the first 5 days of treatment will be the exposure variable. We will also perform a simple per protocol analysis in which patients who received less than 50% of the projected doses in the first 5 days after enrolment will be excluded from the analyses.

[1]Positive septic screen: Presence of any two of the following laboratory parameters: total leucocyte count <5000μL^{-1}; absolute neutrophil count <1500μL^{-1}; band cell: neutrophil ratio > 0.2; micro erythrocyte sedimentation rate >15 mm at 1 hour; C-reactive protein levels >1 mg/dL.
Negative septic screen: Presence of a maximum of one of the above laboratory parameters.
