# Supplementary material for: Zinc as adjunct treatment for clinical severe infection in young infants: A randomized double-blind placebo-controlled trial in India and Nepal
Source: PLoS Med. 2025 Oct 9;22(10):e1004759. doi: 10.1371/journal.pmed.1004759 (PMC12527131; doi:10.1371/journal.pmed.1004759)
Supplement: Attachment — Submitted filename: Zn-PLOS Medicine-Responses to Reviewers Comments_12June2025.docx [file pmed.1004759.s014.docx]

**Zn Sepsis-PLOS Medicine-Responses to Reviewer’s Comments**

Dear Editor,

We would like to sincerely thank you and the reviewers for your valuable comments and the opportunity to revise our manuscript. We have enclosed our point-by-point responses to the feedback, along with a revised version of the manuscript with all changes marked using track changes. We believe that the revisions made in response to the feedback have significantly improved the clarity and quality of the manuscript.

Best regards,

Nitya and Tor

**Comments from the academic editor:**

I have reviewed the paper and agree with most reviewers that it is useful information that should get out. It is unfortunate that they couldn't achieve their planned recruitment. Also with the direction of effects and subgroup analyses, one wishes they had planned a larger and tighter study with solid bacteriology and micronutrient analysis. The bulk of the newborns appear to have late-onset sepsis and clinically suspected sepsis/pneumonia and so one wishes that there was greater effort at recruiting early and a tighter group of bacterial sepsis. Some analysis by maternal nutritional status, breastfeeding patterns and RSV / influenza screens would have been super helpful.

**Response:** Thank you very much for your thoughtful and constructive feedback. We fully acknowledge the limitations noted, including the shortfall in planned recruitment. We have explicitly acknowledged these limitations in the Discussion section of the manuscript.

The primary objective of this investigation was to evaluate the role of zinc as an adjunct to antimicrobial therapy in young infants with late-onset, community-acquired, clinically severe infection—a context in which microbiological confirmation and comprehensive diagnostics are often unavailable, particularly in low-resource settings.

The study was designed as a pragmatic trial to reflect real-world conditions in such environments, where the burden of severe infections is high and adjunctive therapies are urgently needed. While we agree that additional data on bacteriology, maternal nutritional status, breastfeeding patterns, and viral co-infections (such as RSV or influenza) would have strengthened the findings, these were beyond the scope of this trial. We sincerely appreciate your recognition of the relevance of our findings and hope that this work contributes meaningfully to the evidence base guiding the management of clinically severe infection in vulnerable young infants.

**Comments from the reviewers:**

**Reviewer #1: General comments:**

**I found the manuscript to be clearly written. The methods applied are sound and the results well presented.**

**Response**: Thank you very much for your feedback. We appreciate your recognition of the manuscript’s clarity, methodological rigor, and presentation of results.

1. **The interchangeable use of the terms clinically severe infection (CSI) and sepsis is confusing. I would suggest consistently applying CSI. (e.g. lines 60 and 62, line 113 "sepsis like illness))**

**Response**: We appreciate the reviewer’s observation and agree that consistent terminology is essential for clarity. In line with the case definition used in our study, we have revised the manuscript to use the term *clinical severe infection (CSI)* consistently when referring to our trial population. Instances where terms such as “sepsis” or “sepsis-like illness” were previously used have been replaced with “CSI” or clarified, where necessary, to reflect alignment with the study’s operational definition.

In addition to the corrections suggested, we have made edits to ensure consistent use of terminology throughout the manuscript. When referring to findings from other studies, we have retained their original terminology but have clearly contextualized it for the reader.

**2. Please make it clear the term "young infants" refers throughout the manuscript to infants 3 to 59 months of age.**

**Response:** Thank you for pointing this out. In our manuscript, the term “young infants” refers to infants aged 3 to 59 days.

We have now stated this explicitly in the Introduction and revised the following sentence for clarity (page 7; lines 122 - 124):

*‘We conducted a multicenter RCT to estimate the efficacy of adjunct oral zinc in reducing case fatality among young infants aged 3 to 59 days with sepsis, defined as clinical severe infection (CSI) [21, 22].’*

We have also ensured consistent use of the term “young infants”—referring to those aged 3 to 59 days—throughout the manuscript.

**Specific comments:**

**3. Line 106: This sentence seems to imply other treatments could replace antibiotics**.

**Response:** Thank you for the valuable comment. We agree that the sentence could be misinterpreted as implying that alternative treatments might replace antibiotics, which was not our intent. We have revised the sentence to clarify that the objective is to identify adjunctive interventions that complement antimicrobial and supportive therapy, rather than substitute for them.

The revised sentence (page 7; lines 107 - 109) now reads:

*‘It is, accordingly, important to identify inexpensive, effective, and accessible interventions that can be used in addition to antimicrobial and supportive therapy to improve outcomes in severe infections.’*

**4. Line 109: Specify efficacy limited to children 6 to 59 months of age.**

**Response**: Thank you for this valuable suggestion. We have revised the sentence to specify that the efficacy of zinc in reducing the duration of acute diarrhea is established in children aged 6 to 59 months, which is the age group for which current WHO recommendations apply.

The revised sentence (page 7; lines 111 - 112) now reads:

*‘Zinc given orally reduces the duration and severity of acute diarrhea in children aged 6 to 59 months [11–13] and is therefore recommended for its treatment in this age group [14].’*

**5. Vomiting: What proportion had repeated vomiting? How was this managed? Were any excluded?**

**Response**: Thank you for this important question. Vomiting following intervention administration was systematically monitored. In the zinc arm, 40 of 1,576 infants (2.5%) vomited after the first scheduled dose, and 21 of 1,576 infants (1.3%) vomited after re-administration of the same dose. In the placebo arm, 37 of 1,577 infants (2.3%) vomited after the first dose, and 7 of 1,577 infants (0.4%) vomited after re-administration. These data are provided in Supplement 1, Table S4: Adverse events in young infants with clinically severe infection.

As per the study protocol, infants who vomited within 30 minutes of a dose were re-administered the dose. A maximum of two attempts were allowed for each scheduled dose. If vomiting occurred after both attempts, that dose was skipped and the next scheduled dose was administered as planned. No infants were excluded due to vomiting, and none experienced persistent vomiting with every 12-hourly dose during hospitalization.

The procedure for intervention administration detailed in the Methods section, has now been updated for clarity. The revised text (page 10; lines 174 - 180) reads as follows:

*‘Once consent was obtained and the infant was randomized, one tablet of zinc or placebo was dissolved in 2.5 ml of expressed breast milk or clean water and administered to the infant. Subsequent doses were administered every 12 hours. Each dose was re-administered once if vomiting occurred within a 30-minute observation period.* ***If vomiting occurred after both attempts, that dose was skipped and the next scheduled dose was administered as planned.*** *The treatment was given via a nasogastric tube to infants not allowed oral feeds and withheld in instances as defined in the protocol [21].’*

**6. Study limitations: This was a severely malnourished study population (mean wt-for-age Z-score -2.7). This needs to be highlighted with reference to the inability of these infants to respond to the potential immune response of zinc treatment.**

**Response:** Thank you for highlighting this important point. We agree that the high prevalence of severe undernutrition in our study population (mean weight-for-age Z-score –2.7, with approximately 40% of infants below –3Z) is an important contextual factor and have now acknowledged this more explicitly in the discussion.

In our earlier randomized controlled trial published in The Lancet (2012), infants were stratified by nutritional status (weight-for-age <–2Z), and the effect of adjunct zinc was analyzed in both underweight and non-underweight subgroups. While that study showed a 40% overall reduction in treatment failure with adjunct zinc, no significant interaction was observed between nutritional status and treatment effect [18]. Although we did not perform a direct comparison of nutritional profiles across the two study populations, the present trial enrolled a cohort with consistently low weight-for-age (mean Z-score –2.7), indicating a high burden of undernutrition. This may have contributed to an attenuated response to zinc. Given zinc’s well-established role in immune function-particularly in supporting cellular and innate immunity (Shankar and Prasad, 1998; Ref 15)-this remains a biologically plausible consideration, even if not demonstrable in the subgroup analyses of the previous trial. We have incorporated this perspective into the discussion with appropriate caution.

Revised Discussion section (pages 20 - 21; lines 365 - 366):

*‘The limited number of events and the shortfall in achieving the projected sample size are important limitations of this trial. While extremely low attrition minimized the risk of selection bias, the reduced sample size and lower-than-expected event rate resulted in diminished statistical power and an increased risk of Type II error.* ***Additionally, it is possible that several enrolled children were so severely malnourished that they were unable to derive clinical benefit from the adjunct zinc.****’*

**Reviewer #2: This is a challenging study to complete and made more difficult and prolonged by the Covid pandemic. Not accomplishing the planned sample size is a limitation and the reduced power is not of great consequence given how far the primary outcomes are from the hypothesized 30% reduction in mortality.**

**Response:** Thank you for acknowledging the implementation challenges, particularly those posed by the COVID-19 pandemic. We agree that the shortfall in achieving the planned sample size is a limitation, and we have addressed this in the Discussion. Nevertheless, the small observed effect on case fatality, though substantially below the hypothesized 30% reduction, was precisely estimated. We appreciate the reviewer’s perspective that the reduced power is unlikely to have masked a large treatment effect, given the direction and magnitude of the observed outcomes.

**1.The methods should state the exclusion criteria.**

**Response**: Thank you for your observation. We acknowledge that the exclusion criteria were not explicitly stated in the submitted version of the manuscript. These criteria were prespecified in the trial protocol and are detailed in our published protocol paper. Due to word count limitations, we had referenced the published protocol paper for the exclusion criteria and presented the exclusion criteria within Figure 1 (Trial Profile). However, we agree that including the exclusion criteria directly in the Methods section improves clarity and completeness.

Accordingly, we have revised the Methods section as follows (page 9; lines 152 - 157):

‘*All infants aged 3–59 days visiting the hospitals’ emergency units and having at least one of five signs of CSI (stopped feeding well, severe chest indrawing, axillary temperature >38.0⁰C or <35.5⁰⁰C, and movement only when stimulated) [21, 22], were screened for eligibility. They were also required to have been well at some point from birth until the current episode of illness. Infants were excluded if they had a surgical or other medical condition that precluded administration of the study intervention via oral or nasogastric route; had received zinc supplementation within the previous 48 hours; had received injectable antibiotics for ≥48 hours prior to hospitalization; required exchange transfusion; were underweight (weight-for-age z-score <−4.5 or weight <1500 g) at presentation; or were already enrolled in another clinical study [21].’*

2. **The trial profile gives one reason as the condition interfered with oral intake. Then why was NG administration not possible?**

**Response**: Thank you for your comment. The exclusion criteria—now explicitly stated in the *Methods* section—clarify that infants were excluded if they had a surgical or other medical condition that precluded administration of the study intervention, whether orally or via the nasogastric (NG) route. This criterion was prespecified in the trial protocol to ensure that only infants who could safely receive the intervention by either route were enrolled.

Additionally, we have updated Figure 1 on the trial profile figure to clearly reflect this exclusion.

3. **It would be useful to present the gestational age and weight for gestational age and sex if these data are available.**

**Response**: Data on gestational age, weight for gestational age, and sex were not collected for this study and are therefore not available for analysis. We acknowledge that such information would have allowed more detailed characterization of the study population and enriched the interpretation of outcomes.

4. **Clearly using the CSI definition did not identify many children with likely sepsis is indicated by the laboratory findings and the recovery of 50% by day 2 and 75% by day 4. In this population there is clearly no clinically meaningful benefit of zinc. The suggestion of benefit in infants with confirmed or more likely sepsis is consistent with previous studies, but as the authors indicate would need to be confirmed before zinc can be recommended.**

**Response:** Thank you for this thoughtful comment. We agree that the use of the clinically severe infection (CSI) definition likely encompassed a broad spectrum of illness severity, including infants with self-limiting or non-bacterial infections, influencing both recovery rates and treatment effect estimates in the overall cohort.

The CSI definition was selected to align with WHO programmatic criteria and to facilitate early intervention of adjunctive therapy in low-resource settings, where access to confirmatory diagnostics is limited. While this operational definition enhances generalizability, it may reduce specificity for true bacterial sepsis and dilute the potential benefits of zinc in the larger study population.

Although the observed 17% reduction in mortality did not reach statistical significance, we consider this effect clinically meaningful, particularly in low resource settings with constrained treatment options.

Importantly, in our earlier randomized controlled trial published in The Lancet (2012), which included infants with probable serious bacterial infection defined by CRP ≥12 mg/L, adjunct zinc reduced treatment failure by 40% and showed a similar trend towards lower mortality [18]. These findings are consistent with the suggestion of benefit in infants with confirmed or probable sepsis and support the rationale for targeted trials in such subgroups before broad policy recommendations can be made. Our current manuscript already reflects this cautious interpretation.

As stated in the Conclusion section (page 21; lines 373 - 379):

‘*While our prespecified and post-hoc subgroup analyses may suggest a potential beneficial effect of zinc supplementation in subgroups of infants with laboratory parameters or clinical signs suggestive of more severe illness, these findings are exploratory and require cautious interpretation due to the wide confidence intervals and the absence of any effects on death during the 12-week follow-up period. We suggest that the effect of zinc in young infant sepsis be explored in other randomized controlled trials before considering its inclusion in treatment guidelines.’*

**Reviewer #3: Statistical review**

**This paper reports a randomised trial of zinc vs placebo for infection in young infants. The trial was large and reported well, giving useful data. I only had some minor comments:**

1. **Abstract: I would recommend that p-values for the primary outcomes are provided as well as the confidence interval: the study was powered to test that hypothesis.**

**Response**: Thank you for this helpful suggestion. We agree that the inclusion of p-values improves clarity and completeness. We have now added the relevant p-values for the primary outcomes in the text of the manuscript.

2. **Page 10: "(death from enrollment till discharge from hospital)" - I found this a bit confusing due to the two 'from's, perhaps 'hospital discharge' would avoid that.**

**Response**: Thank you for pointing this out. We agree that the phrase is confusing. We have revised it to “death until hospital discharge” to improve clarity.

3. **Page 11, outcomes: there were a couple of secondary outcomes on the trial registration page that were not mentioned here - would these be reported in other work? It would be good to mention this if so.**

**Response**: Thank you for drawing attention to this. This manuscript focuses on the primary outcome and selected secondary outcomes related to the acute illness episode. Other prespecified secondary outcomes- including those assessing immunobiological responses, cost-effectiveness, health gain, and financial risk protection- are planned to be reported in subsequent publications.

To clarify this for readers, we have added the following sentence to the end of the Outcomes section (pages 11 - 12; lines 214 - 216):

*‘Additional prespecified secondary outcomes, including immunobiological effects, cost-effectiveness analyses, and measures of financial risk protection, will be reported in future publications.’*

4. **Page 11 - could the assumed control event rate be added for the power calculation as well as the between group difference? I note the 10% inflation in sample size for attrition - could it be stated how missing outcome data was handled in the analysis? Presumably it was complete cases, which seems appropriate given the very low actual loss to follow-up.**

**Response**: We appreciate this helpful observation. We have now included the assumed control event rate used in the section on sample size calculation. As described in our published protocol, the expected case fatality risk among infants <2 months with clinical severe infection in similar settings was 10%, based on recent audits from district hospitals in India. This reference event rate, along with an anticipated ≥30% relative risk reduction in the zinc arm, informed our sample size estimate. A 10% inflation was applied to account for potential attrition.

We have also clarified how missing outcome data were handled. As specified in our statistical analysis plan, the primary analysis followed an intention-to-treat approach including all randomized infants with known outcomes. Given the minimal loss to follow-up, analyses were conducted on a complete case basis.

We have revised the manuscript to read as follows (page 12; lines 226 - 231):

*‘Our calculated sample size of 3,762 infants (1,881 per trial arm) was based on an assumed 10% case fatality risk among infants <2 months with clinical severe infection, to identify ≥30% relative risk reduction of death, assuming a reference event rate of 10% with 95% confidence and 90% power. This was inflated to 4,140 to account for an anticipated 10% attrition [21]. The analyses were done on an intention-to-treat basis and according to a predefined protocol and statistical analysis plan [21]. Analyses were conducted on a complete case basis, given the minimal loss to follow-up.’*

**Page 17 - "The median time to death until end of the 12-week study period was similar in the two trial arms (18 days [IQR 6-37 days] in zinc arm; 12 days [IQR 4-32 days]": I think this should be the median time to death amongst those who died (as fewer than 50% of participants died, there's no median estimable from the trial data).**

**Response**: Thank you for your valuable comment. We confirm that the median time to death refers specifically to infants who died during the 12-week study follow-up period, and not the entire trial population.

We have revised the sentence in the manuscript to clarify this (page 18; lines 294 - 299):

*‘During the period from enrollment to the end of the 12-week (84-day) study period, 140 of 1,554 infants (9.0%) died in the zinc arm, and 133 of 1,550 infants (8.6%) died in the placebo arm (RR 1.05 (95% CI [0.84, 1.32]; p=0.674)). Among infants who died, the median time to death was similar in the two trial arms: 18 days [IQR 6–37 days] in the zinc arm and 12 days [IQR 4–32 days] in the placebo arm; hazard ratio [HR] 1.05 (95% CI [0.83, 1.33]; p=0.681) (Table 2,* ***Fig 2a****).’*

5. **Discussion - given the mention of potential benefit from zinc in more severe subgroups, it would be useful to add if any of the subgroup analyses had significant interaction tests in the results section.**

**Response:** Thank you for this valuable suggestion. While formal interaction testing was not part of our original statistical analysis plan, we conducted post hoc analyses across all predefined subgroups using the Absolute Excess Risk due to Interaction (AERI) method. None of the interaction terms were statistically significant for the primary outcome or the main secondary outcome (treatment failure), except for an interaction by country on treatment failure (*p*=0.04). As no adjustment was made for multiple comparisons, this finding should be interpreted with caution. We would be happy to include a brief statement on interaction testing in the *Results* section if deemed appropriate.

James Watson

Any attachments provided with reviews can be seen via the following link:
--------------------------------------------------------- ---

General editorial requests:
(Please note: not all will apply to your paper, but please check each item carefully)

*	We ask every co-author listed on the manuscript to fill in a contributing author statement, making sure to declare all competing interests. If any of the co-authors have not filled in the statement, we will remind them to do so when the paper is revised. If all statements are not completed in a timely fashion this could hold up the re-review process. If new competing interests are declared later in the revision process, this may also hold up the submission. Should there be a problem getting one of your co-authors to fill in a statement we will be in contact. Please do not add or remove authors without first discussing this with the handling editor. You can see our competing interests policy here: <http://journals.plos.org/plosmedicine/s/competing-interests>.

*	Please upload any figures associated with your paper as individual TIF or EPS files with 300dpi resolution at resubmission; please read our figure guidelines for more information on our requirements: <http://journals.plos.org/plosmedicine/s/figures>. While revising your submission, please upload your figure files to the PACE digital diagnostic tool, <https://pacev2.apexcovantage.com/>. PACE helps ensure that figures meet PLOS requirements. To use PACE, you must first register as a user. Then, login and navigate to the UPLOAD tab, where you will find detailed instructions on how to use the tool. If you encounter any issues or have any questions when using PACE, please email us at PLOSMedicine@plos.org.

**Response:** All figures associated with the manuscript have been uploaded as individual files with 300dpi resolution as per the PLOS Medicine formatting requirement.

*	Please ensure that the paper adheres to the PLOS Data Availability Policy (see <http://journals.plos.org/plosmedicine/s/data-availability>), which requires that all data underlying the study's findings be provided in a repository or as Supporting Information. For data residing with a third party, authors are required to provide instructions with contact information (web or email address) for obtaining the data. Please note that a study author cannot be the contact person for the data. PLOS journals do not allow statements supported by "data not shown" or "unpublished results." For such statements, authors must provide supporting data or cite public sources that include it.

**Response:** We have updated the ‘Data availability statement’ to align with the PLOS Medicine requirement.

*	We expect all researchers with submissions to PLOS in which author-generated code underpins the findings in the manuscript to make all author-generated code available without restrictions upon publication of the work. In cases where code is central to the manuscript, we may require the code to be made available as a condition of publication. Authors are responsible for ensuring that the code is reusable and well documented. Please make any custom code available, either as part of your data deposition or as a supplementary file. Please add a sentence to your data availability statement regarding any code used in the study, e.g. "The code used in the analysis is available from Github [URL] and archived in Zenodo [DOI link]" Please review our guidelines at <https://journals.plos.org/plosmedicine/s/materials-software-and-code-sharing> and ensure that your code is shared in a way that follows best practice and facilitates reproducibility and reuse. Because Github depositions can be readily changed or deleted, we encourage you to make a permanent DOI'd copy (e.g. in Zenodo) and provide the URL.

*	Data availability: PLOS Medicine requires that the de-identified data underlying the specific results in a published article be made available, without restrictions on access, in a public repository or as Supporting Information at the time of article publication, provided it is legal and ethical to do so.
The Data Availability Statement (DAS) requires revision. Note that a study author cannot be the contact person for the data.

For each data source used in your study:
a) If the data are freely or publicly available, note this and state the location of the data: within the paper, in Supporting Information files, or in a public repository (include the DOI or accession number).
b) If the data are owned by a third party but freely available upon request, please note this and state the owner of the data set and contact information for data requests (web or email address). Note that a study author cannot be the contact person for the data.
c) If the data are not freely available, please describe briefly the ethical, legal, or contractual restriction that prevents you from sharing it. Please also include an appropriate contact (web or email address) for inquiries (again, this cannot be a study author).

**Response:** We have updated the ‘Data availability statement’ to align with the PLOS Medicine requirement.

*	If your trial had to undergo important modifications in response to extenuating circumstances such as the COVID-19 pandemic, please complete the CONSERVE-CONSORT checklist and provide in your Supporting Information; (<https://www.equator-network.org/reporting-guidelines/guidelines-for-reporting-trial-protocols-and-completed-trials-modified-due-to-the-covid-19-pandemic-and-other-extenuating-circumstances-the-conserve-2021-statement/>). When completing the checklist, please use section and paragraph numbers, rather than page numbers.

**Response:** Our trial did not undergo major modifications in response to extenuating circumstances such as the COVID-19 pandemic.

FORMATTING - GENERAL
*	Abstract: Please structure your abstract using the PLOS Medicine headings (Background, Methods and Findings, Conclusions). Please combine the Methods and Findings sections into one section.

**Response:** Our Abstract is structured as per the PLOS Medicine formatting requirement.

*	At this stage, we ask that you include a short, non-technical Author Summary of your research to make findings accessible to a wide audience that includes both scientists and non-scientists. The Author Summary should immediately follow the Abstract in your revised manuscript. This text is subject to editorial change and should be distinct from the scientific abstract. Ideally each sub-heading should contain 2-3 single sentence, concise bullet points containing the most salient points from your study. In the final bullet point of 'What Do These Findings Mean?', please include the main limitations of the study in non-technical language. Please see our author guidelines for more information: <https://journals.plos.org/plosmedicine/s/revising-your-manuscript#loc-author-summary>.

**Response:** Thank you. We have moved the Author Summary section to after the Abstract as per the PLOS Medicine formatting requirement.

*	Please express the main results with 95% CIs as well as p values. When reporting p values please report as p<0.001 and where higher as the exact p value p=0.002, for example. Throughout, suggest reporting statistical information as follows to improve clarity for the reader "22% (95% CI [13%,28%]; p</=)". Please be sure to define all numerical values at first use.

**Response:** Thank you for your comment. We have reported p-values along with 95% confidence intervals for the main results reported in the text of the manuscript. Where applicable, p-values are now presented as *p*<0.001 or as exact values (e.g., *p*=0.002), in line with the PLOS Medicine formatting requirement. We have also ensured that all numerical values are clearly defined at first use for clarity.

*	Please include page numbers and line numbers in the manuscript file. Use continuous line numbers (do not restart the numbering on each page).

**Response:** Page and line numbers are included in the manuscript file.

*	Please cite the reference numbers in square brackets. Citations should precede punctuation.

**Response:** Reference numbers have been cited following the PLOS Medicine requirement.

FIGURES AND TABLES
*	Please provide titles and legends for all figures and tables (including those in Supporting Information files).

*	Please define all abbreviations used in each figure/table (including those in Supporting Information files).

*	Please consider avoiding the use of red and green in order to make your figure more accessible to those with color blindness.

**Response:** Thank you. Titles and legends for all figures and tables have been provided and all abbreviations have used in each figure/table have been defined at first use

SUPPLEMENTARY MATERIAL
*	Please note that supplementary material will be posted as supplied by the authors. Therefore, please amend it according to the relevant comments outlined here.

*	Please cite your Supporting Information as outlined here: <https://journals.plos.org/plosmedicine/s/supporting-information>

REFERENCES
*	PLOS uses the numbered citation (citation-sequence) method and first six authors, et al.

*	Please ensure that journal name abbreviations match those found in the National Center for Biotechnology Information (NCBI) databases (<http://www.ncbi.nlm.nih.gov/nlmcatalog/journals>), and are appropriately formatted and capitalised.

*	Where website addresses are cited, please include the complete URL and specify the date of access (e.g. [accessed: 12/06/2023]).

*	Please also see <https://journals.plos.org/plosmedicine/s/submission-guidelines#loc-references> for further details on reference formatting.

**RCTs**

*	PLOS Medicine requires that all trials be prospectively registered in one of registries recognized by WHO. Please ensure that study registration details are included in the Methods section.

*	Please structure the Methods section using the following sub-headings: Study design and participants, Randomization and masking, Procedures, Outcomes, Statistical analysis.

**Response:** We have structured the Methods section using the sub-headings: Study design and participants, Randomization and masking, Procedures, Outcomes, Quality assurance and quality control, Statistical analysis.

*	Amendments to the protocol are outlined in detail in the supplementary file but are not described in the Methods section of the main manuscript text. Please incorporate some of this information in the Methods section.

**Response:** Thank you for your observation. As noted, the ‘Supplement Appendix’ outlines all protocol amendments. Some of these—including the revision of the age eligibility from 1–59 days to 3–59 days and the addition of the criterion that infants must have been well at some point in the first 48 hours of life—were made prior to trial initiation (February 28, 2017) and are reflected in the published protocol paper.

A few additional amendments were made in June 2017, very early in the trial, based on operational feasibility and ethical considerations, without compromising participant safety or scientific integrity. These included:

- Extension of the stabilization window from 8 to 24 hours to improve enrolment of clinically eligible but initially unstable infants
- Modification of the exclusion criterion from *weight-for-age z-score < –4.5* to *weight <1500 g* to avoid unjustified exclusion of vulnerable low birthweight infants

All protocol amendments were implemented only after approval from the relevant Institutional Ethics Committees. Due to space limitations, some of the post-initiation amendments were not included in the main *Methods* section.

We have now added a brief summary of two key amendments under the subheading *Study design, setting and participants*, as follows:

*‘Two key protocol amendments were implemented in June 2017, shortly after trial initiation: (i) the stabilization window was extended from 8 to 24 hours to allow clinically eligible but initially unstable infants additional time to stabilize, and (ii) the exclusion criterion was revised from weight-for-age z-score < –4.5 to weight <1500 gm to avoid excluding low birthweight infants who were at higher risk for poor outcomes, but for whom there was no evidence of increased risk associated with zinc supplementation. The amendments were approved by all relevant Institutional Ethics Committees prior to implementation.’ (page 8; lines 139 - 146)*

*	Confirmation that all amendments were approved by relevant IRBs is lacking in the Methods section of the main manuscript text.

**Response:** Thank you for your comment. As noted above, all protocol amendments were implemented only after obtaining approval from the relevant Institutional Ethics Committees.

We have now added a sentence in the *Methods* section of the main manuscript, to clarify this.

*‘The amendments were approved by all relevant Institutional Ethics Committees prior to implementation.’ (page 8; lines 145 - 146)*

*	Please comment on the statistical power of your trial in the main manuscript text. Planned recruitment appeared to be 2070 per arm, but reached ~1500 per arm.

**Response:** Thank you for your comment. The statistical power of the trial was indeed impacted by two factors: (i) we were able to recruit only about 75% of the planned sample size, and (ii) the number of events observed in the placebo arm was approximately half of what was anticipated.

This limitation has been acknowledged in the *Discussion* section of the manuscript, which now reads as follows (pages 20 - 21; lines 361 - 366):

*‘The limited number of events and the shortfall in achieving the projected sample size are important limitations of this trial. While extremely low attrition minimized the risk of selection bias, the reduced sample size and lower-than-expected event rate resulted in diminished statistical power and an increased risk of Type II error. Additionally, it is possible that several enrolled children were severely malnourished that they were unable to derive clinical benefit from the adjunct zinc.’*

*	The following inclusion criterion is missing in the Methods section of the manuscript text: “Infant should have been well at some point from birth till the current episode of illness.”

**Response:** Thank you for pointing this out. We acknowledge that the inclusion criterion—“Infant should have been well at some point from birth till the current episode of illness”—was inadvertently omitted from the *Methods* section of the manuscript.

This criterion was part of the approved and published trial protocol and has now been added to the *Methods* section of the main manuscript along with other eligibility criteria.

*‘All infants aged 3–59 days visiting the hospitals’ emergency units and having at least one of five signs of CSI (stopped feeding well, severe chest indrawing, axillary temperature ≥38.0⁰C or <35.5⁰C and movement only when stimulated) [21, 22], were screened for eligibility. T****hey were also required to have been well at some point from birth until the current episode of illness****.*’ (page 9; lines 150 - 151)

*	Please outline exclusion criteria in the Methods section.

**Response:** Thank you for the comment. We acknowledge that the exclusion criteria were not explicitly stated in the original *Methods* section. These criteria were prespecified in the trial protocol and are detailed in our published protocol paper. While we had referenced the protocol and presented the criteria in Figure 1 (Trial Profile), we agree that including them directly in the text enhances clarity.

We have now revised the *Methods* section (page 9; lines 152 - 157) to include the following:

‘*All infants aged 3–59 days visiting the hospitals’ emergency units and having at least one of five signs of CSI (stopped feeding well, severe chest indrawing, axillary temperature >38.0⁰C or <35.5⁰⁰C, and movement only when stimulated) [21, 22], were screened for eligibility. Infants were excluded if they had a surgical or other medical condition that precluded administration of the study intervention via oral or nasogastric route; had received zinc supplementation within the previous 48 hours; had received injectable antibiotics for ≥48 hours prior to hospitalization; required exchange transfusion; were underweight (weight-for-age z-score <−4.5 or weight <1500 g) at presentation; or were already enrolled in another clinical study [21].’*

*	Please clarify and explain all discrepancies between the paper and protocol. If the outcomes were not prespecified in the protocol, please define them in the Methods (Outcomes section) as post hoc and explain why they were added. Post-hoc comparisons should be presented as hypothesis-generating rather than conclusive.

**Response:** Thank you for highlighting this important point. This manuscript reports on the primary outcome and a subset of prespecified secondary outcomes related to the acute illness episode. Other prespecified secondary outcomes—such as immunobiological responses, cost-effectiveness, and financial risk protection—are planned for analysis and reporting in subsequent publications.

To clarify this for readers, we have added the following sentence at the end of the *Outcomes* section (pages 11 - 12; lines 214 - 216):

*‘Additional prespecified secondary outcomes, including immunobiological effects, cost-effectiveness analyses, and measures of financial risk protection, will be reported in future publications.’*

We confirm that all outcomes presented in this manuscript were prespecified in the trial protocol. No post hoc outcomes are reported. Any future exploratory or post hoc analyses will be clearly identified as such and interpreted as hypothesis-generating.

*	Please ensure that all prespecified outcomes (primary, secondary, and exploratory) are listed in the Methods/Outcomes section and indicate whether there are outcomes that are not presented in the current report. More specifically:
- Please provide more detail on all secondary outcomes in the Methods section of the manuscript text.

**Response:** Please refer to the response above

- Results for cessation of signs of clinical severe infection is not reported in the main text.

**Response:** Thank you for your comment. Cessation of signs of clinical severe infection (CSI) is a secondary outcome and listed under the subheading *Outcome* in the *Methods* section (page 11; line 212). In the *Results* section, we report that ‘........ the time until recovery from CSI were similar in the two arms (Table 2, Fig 2b).’ (page 18; lines 307 - 308), with supporting data presented in Table 2 and Figure 2b.

Some time-to-event analyses missing in the main text.

**Response:** Thank you for your comment. As outlined in the protocol and statistical analysis plan, several time-to-event analyses were prespecified. In this manuscript, we report time to cessation of signs of clinical severe infection (CSI) as a secondary outcome, as time to recovery was deemed a more clinically meaningful indicator of treatment response. This is presented in the *Results* section ‘........ the time until recovery from CSI were similar in the two arms (Table 2, Fig 2b).’ (page 18; lines 307 - 308)

For other time-to-event outcomes—such as time to death during hospitalization, time to death until the end of 12-week follow-up, time to treatment failure, and time to hospital discharge—the results were similar between the two trial arms. As we reported risk estimates for these outcomes in the main text (e.g., risk of death, risk of treatment failure), we did not present the corresponding time-to-event analyses to avoid redundancy, particularly where findings did not differ meaningfully between groups.

- Secondary outcomes relating to reappearance of CSI, discharge, CEA and Immunobiological readouts are not reported and should be outlined in the methods (even if results not reported).

**Response:** Thank you for your comment. We would like to clarify that the *Results* section includes the following secondary outcomes: *“The risk of severe illness requiring hospitalization, the median duration of hospitalization, and the time until recovery from CSI were similar in the two arms”* (Table 2, Fig 2b).

This manuscript focuses on the primary outcome and selected secondary outcomes related to the acute illness episode. Other prespecified secondary outcomes—including those assessing the reappearance of CSI, time to discharge, immunobiological responses, cost-effectiveness, health gain, and financial risk protection—are planned to be reported in subsequent publications.

To make this clear to readers, we have added the following sentence to the end of the *Outcomes* section (pages 11 - 12; lines 214 - 216):

*‘Additional prespecified secondary outcomes, including immunobiological effects, cost-effectiveness analyses, and measures of financial risk protection, will be reported in future publications.’*

- Subgroup analyses relating to age at enrolment appear post-hoc (3–6 days, 7–28 days, and 29–59 days). Please clarify.

**Response:** Thank you for your comment. We would like to clarify that subgroup analyses based on age at enrolment—specifically, <7 days and ≥7 days—were prespecified in both the protocol paper and the statistical analysis plan (as registered on the CTRI). In the current analysis, we further stratified the ≥7-day group into 7–28 days and 29–59 days to explore potential differential effects of adjunct zinc in neonates versus older infants beyond the neonatal period.

- Subgroup analyses were also planned for secondary outcomes, but have not been included in this report. Please clarify.

**Response:** Prespecified and post hoc subgroup analyses for the secondary outcome of treatment failure are presented in the Supplement 1 (see S1 Fig and S1 Table). These analyses were not included in the main text due to space constraints but are available to readers in full in the Supplement.

- Some per-protocol analyses were planned but are not included in this report. Please clarify.

**Response:** Thank you for your comment. As stated in the protocol and SAP (CTRI), a per-protocol analysis was prespecified, excluding infants who received less than 50% of doses in the first five days. As shown in Supplement 1 (S2 Table), nearly 90% of participants in both arms met this threshold, indicating high compliance. Given the minimal difference between per-protocol and full populations, and space constraints, these analyses were not included in the manuscript. However, we would be happy to include them in the Supplement if required.

*	Please specify the dates (Month Day, Year) during which study enrollment and follow up occurred.

**Response:** The study was initiated on February 28, 2017; the last infant was enrolled on November 30, 2021, and the final 12-week follow-up was completed on February 22, 2022. These dates are explicitly stated in the *Methods* (study period) and *Results* (screening and enrolment) sections of the manuscript.

*	Please include absolute numbers wherever you report percentages; eg, n/N (%)

**Response:** We have included absolute numbers wherever we report percentages

*	Please present the safety data for the study including numbers of specific events and whether or not adverse events are thought to be related to treatment. AEs should be reported in the abstract, per CONSORT and CONSORT-Harms.

**Response:** Thank you for your comment. Safety data are presented in Supplementary Table S4. As stated in the manuscript, infants were closely monitored for adverse events in accordance with the protocol and national guidelines. Adverse events were generally similar between groups, with a slight increase in vomiting in the zinc arm. No events were attributed to zinc. We have now added a brief summary of safety findings to the *Abstract*, in line with CONSORT-Harms recommendations.

*‘Adverse events were similar across trial arms, except for a slight increase in vomiting in the zinc arm; no events were attributed to the intervention.’* (pages 3 - 4; lines 56 - 58)

We have also revised the statement on adverse events in the main text. It now reads as ‘*Except for a slight increase in the risk of vomiting, we found no other negative effects of zinc* *(****S4*** ***Table****), an no adverse events were considered attributable to the intervention.’* (page 19; lines 330 - 331)

*	Please complete the CONSORT checklist (<https://www.equator-network.org/reporting-guidelines/consort/>) and ensure that all components of CONSORT are present in the manuscript, including how randomization was performed, allocation concealment, blinding of intervention, definition of lost to follow-up, power statement. When completing the checklist, please use section and paragraph numbers, rather than page numbers.

**Response:** Thank you for your comment. We have revised the CONSORT checklist to reflect the updated manuscript and ensured that all key elements—including randomization procedure, allocation concealment, blinding, definition of loss to follow-up, and the power calculation—are clearly addressed. The checklist now uses section and paragraph numbers as requested.

*	Please report your abstract according to CONSORT for abstracts, following the PLOS Medicine abstract structure (Background, Methods and Findings, Conclusions) <https://www.equator-network.org/reporting-guidelines/consort-abstracts/>

**Response:** The Abstract is reported as per the CONSORT for abstracts following the PLOS Medicine abstract structure (Background, Methods and Findings, Conclusions)

*	If your trial had to undergo important modifications in response to extenuating circumstances, please complete the CONSERVE-CONSORT checklist and provide in your Supporting Information; (<https://www.equator-network.org/reporting-guidelines/guidelines-for-reporting-trial-protocols-and-completed-trials-modified-due-to-the-covid-19-pandemic-and-other-extenuating-circumstances-the-conserve-2021-statement/>). When completing the checklist, please use section and paragraph numbers, rather than page numbers.

**Response:** Our trial did not undergo major modifications in response to extenuating circumstances such as the COVID-19 pandemic.

*	In keeping with our commitment to Open Science, please include the study protocol document and analysis plan (including any amendments) as Supporting Information to be published with the manuscript if accepted.

**Response:** All supporting documents including study protocol and statistical analysis plan have been submitted as Supplement Appendix.

*	Please note that PLOS Medicine requires prospective, public registration of a data sharing plan (as part of mandatory clinical trials registration) for all clinical trials that began enrollment on or after January 1, 2019, in accordance with ICMJE requirements.

**Response:** Thank you for the clarification. We note this requirement; however, our trial was initiated on February 28, 2017, prior to the ICMJE mandate on prospective registration of data sharing plans.

_________________________________________________
In compliance with data protection regulations, you may request that we remove your personal registration details at any time. (Use the following URL: <https://www.editorialmanager.com/pmedicine/login.asp?a=r>). Please contact the publication office if you have any questions.
