# Supplementary material for: Zinc as adjunct treatment for clinical severe infection in young infants: A randomized double-blind placebo-controlled trial in India and Nepal
Source: PLoS Med. 2025 Oct 9;22(10):e1004759. doi: 10.1371/journal.pmed.1004759 (PMC12527131; doi:10.1371/journal.pmed.1004759)
Supplement: Attachment — Submitted filename: Zn-PLOS Medicine_Response to comments_06thAug2025.docx [file pmed.1004759.s015.docx]

**Zn Sepsis-PLOS Medicine-Responses to Editor and Reviewer Comments**

Dear Editor**,**

We sincerely thank you and the reviewers for your thoughtful comments and for the decision to accept our manuscript, subject to minor editorial and production revisions. We have carefully addressed all remaining requests and are submitting a point-by-point response to each comment. A revised version of the manuscript has also been submitted, along with a marked-up copy highlighting the changes. We believe these final revisions have further improved the clarity and quality of the paper.

Best regards
Nitya and Tor

------------------------------------------------------------

Requests from Editors:

**GENERAL:**

**Comment:** Please amend your title to "Zinc as adjunct treatment for suspected clinical severe infection in young infants: A randomized double-blind placebo-controlled trial in India and Nepal".

**Response:** Thank you for the suggestion. While we appreciate the suggestion and have revised the abstract to clarify that clinical severe infection (CSI) refers to suspected sepsis, we are hesitant to include the word *'suspected'* in the title before *clinical severe infection*. This would be inconsistent with how the term CSI is used in the context of our manuscript and the broader literature, including the AFRINEST study we cite (Ref 22), where it denotes the presence of clinical signs of severe infection**.**

We have amended the manuscript title to:
"Zinc as adjunct treatment for clinical severe infection in young infants: A randomized double-blind placebo-controlled trial in India and Nepal."

**Comment:** Please ensure that all abbreviations are defined at first use throughout the text.

**Response:** Thank you. All abbreviations used have been defined at first use.

**Comment:** Please confirm that all numbers presented in the abstract are present and identical to numbers presented in the main manuscript text.

**Response:** Thank you for this comment. We have reviewed the abstract and main text to ensure consistency of numerical data. We have now added the number and proportion of female infants (1,203 [38%] of 3,153) to the Results section of the main manuscript text to match the information presented in the abstract.

The revised para in the main manuscript text reads as follows (pages 14 –15; lines 288 – 295):

*The participants in the two trial arms had comparable baseline characteristics* ***(Table 1****). The enrolled cohort included 1,203 (38%) females. The median age at enrollment was 25 days (interquartile range [IQR] 13–41 days), and the mean weight was 2.9 kg (standard deviation [SD] 0.8). The median duration of symptoms before admission into the study was 48 hours (IQR 24–72 hours). Approximately 40% (1,047 of 2,529) of the babies had CRP >10 mg/L, 24% (726 of 3,089 infants) had a positive septic screen, and we found potentially pathogenic bacteria in 7.6% (208 of 2,742 infants) of the blood cultures. The 14-day course of study treatment was completed by 99% of participants in both trial arms* ***(S2 Table).***

**ABSTRACT:**

**Comment:** Please include ‘arm’ after ‘zinc’ in the following sentence: “During 12 weeks from enrolment, 140 of 1,554 infants (9.0%) died in the zinc, and 133 of 1,550 infants (8.6%) in the placebo arm (RR 1.05 (95% CI [0.84, 1.32]; p=0.674)).”

**Response:** Thank you for pointing this out. We have revised the sentence to include the word ‘arm’ after ‘zinc’ as requested.

The corrected sentence now reads (page 3; lines 56–58):
*Among those who completed 12 weeks of follow-up, 140 of 1,554 infants (9.0%) died in the zinc arm, and 133 of 1,550 (8.6%) in the placebo arm (RR 1.05 (95% CI [0.84, 1.32]; p=0.674)).*

**Comment:** In the last sentence of the Abstract Methods and Findings section, please describe the main limitation(s) of the study's methodology.

Please include the fact that the study is underpowered as a limitation.

**Response**: Thank you for this suggestion. We have revised the last sentence of the Abstract’s Methods and Findings section to include the primary methodological limitation. Specifically, we now note that the study was underpowered due to a shortfall in sample size and lower-than-anticipated event rates.

The revised sentence of the Abstract’s Methods and Findings section is as below (pages 3 – 4; lines 59 – 61):

*The main limitation of the study is that it was underpowered due to lower-than-anticipated event rates and a shortfall in the achieved sample size.*

**Comment:** line 38: are the mentioned deaths attributable to infection? please clarify this.

**Response**: Thank you for this helpful comment. The original sentence was intended to indicate that sepsis is a major contributor to neonatal deaths, not that all deaths were attributable to infection. We had kept the phrasing concise to adhere to the abstract word limit, but we recognize that the meaning may not have been sufficiently clear. We have now revised the sentence to improve clarity.

The modified section now reads (page 3; lines 38 – 40):

*Annually, an estimated 2.3 million infants die within their first month of life, primarily in sub-Saharan Africa and South Asia. Infections, including sepsis, are among the major contributors to these deaths.*

**Comment:** Line 43—needs a period.

**Response**: Thank you for pointing this out. We have added the missing period as suggested.

**Comment:** Line 48: state whether this was a confirmed or a suspected infection.

**Response**: We appreciate the opportunity to clarify this point. We have revised the Abstract to state that the sepsis was suspected, not microbiologically confirmed. It now specifies that enrolled infants had suspected sepsis, defined as clinical severe infection based on clinical signs adapted from the WHO Integrated Management of Childhood Illness (IMCI) criteria.

### Revised sentence for Abstract (page 3; lines 47 – 51):

### *Infants aged 3–59 days hospitalized with suspected sepsis, defined as clinical severe infection (CSI), adapted from the WHO Integrated Management of Childhood Illness (IMCI) criteria, were randomly assigned to receive 10 mg of elemental zinc daily or placebo orally for 14 days, in addition to standard of care.*

**Comment:** Line 49: State that zinc or placebo was added to SOC (Standard of care).

**Response**: We thank the reviewer for the helpful suggestions. We have revised the sentence to clarify that the infants had suspected sepsis, defined as clinical severe infection and that zinc or placebo was given in addition to standard of care treatment.

The revised sentence in the Abstract reads as follows (page 3; lines 47 – 51):

### *Infants aged 3–59 days hospitalized with suspected sepsis, defined as clinical severe infection (CSI), adapted from the WHO Integrated Management of Childhood Illness (IMCI) criteria, were randomly assigned to receive 10 mg of elemental zinc daily or placebo orally for 14 days, in addition to standard of care.*

**Comment:** Line 53: replace 'against' with 'compared to' or 'and'

**Response**: Thank you for the suggestion. We have replaced ‘against’ with ‘compared to’ as recommended (page 3; line 54).

**Comment:** Line 55: Please clarify why the number of total infants is different between the hospital stay vs 12 weeks. (e.g due to loss of follow-up)

**Response**: Thank you for this comment. We have revised the sentence to clarify that the reported 12-week mortality includes only infants who completed follow-up. This explains the smaller denominators compared to in-hospital mortality data, which included all randomized infants.

The revised sentence reads as follows (page 3; lines 56 – 58):

*Among those who completed 12 weeks of follow-up, 140 of 1,554 infants (9.0%) died in the zinc arm, and 133 of 1,550 (8.6%) in the placebo arm (RR 1.05 (95% CI [0.84, 1.32]; p=0.674)).*

**AUTHOR SUMMARY:**

**Comment:** Please move the last bullet point in ‘why was this study done’ to ‘what did the researchers do and find.

**Response**: We have removed the last bullet point from the 'Why Was This Study Done?' section and moved it to the 'What Did the Researchers Do and Find?' section, in accordance with the suggestion.

**Comment:** Please remove ‘well-designed’ from your author summary: “This well-designed, large, multisite randomized controlled trial (RCT) was implemented to evaluate oral zinc as an adjunct to standard antibiotic therapy in reducing the risk of death among young infants with clinical severe infection (CSI)”

**Response**: We have removed the term ‘well-designed’ from the Author Summary to ensure objectivity.

**Comment:** Please remove the first bullet point in ‘what did the researchers do and find’ (Our study, undertaken at several institutions in two Asian countries, is the largest RCT on the effect of zinc as an adjunct to standard antimicrobial therapy in reducing death during hospitalization and within 12 weeks after enrolment among young infants with CSI.)

**Response**: We appreciate this helpful suggestion. We have revised the 'What Did the Researchers Do and Find?' section by removing the original first bullet point and replacing it with a more concise description of the study's objective and design, previously included in the 'Why Was This Study Done?' section.

**Comment:** Please remove the last bullet point (line 100-102)  under 'What Do These Findings Mean?' (as this is redundant with the previous bullet point)

**Response**: Thank you for highlighting this. We agree that the final bullet point under 'What Do These Findings Mean?' was redundant and have removed it.

**Comment:** In the author summary, in the final bullet point of 'What Do These Findings Mean?', please include the main limitations of the study in non-technical language. Please include the fact that the study is underpowered as a limitation.

**Response**: Thank you for your comment. We have added a final bullet point in the 'What Do These Findings Mean?' section of the Author Summary to describe the main limitation of the study using non-technical language, including the fact that the study was underpowered.

The final bullet point under ‘What Do These Findings Mean?’ reads as follows (page 6; lines 101 –103):

- *One limitation of this study is that fewer infants were enrolled than planned, and fewer deaths occurred than expected, which reduced the ability to detect small differences between the groups*.

**Comment:** Please ensure that all text is objective.

**Response:** Thank you for your comment. We have reviewed the Author Summary carefully to ensure that the language used throughout is objective and evidence-based. We have removed or revised any potentially evaluative terms (e.g., ‘well-designed’) and confirmed that the summary neutrally describes the study rationale, design, findings, and implications without overstatement.

**Comment:** Please rephrase line 77, which states that zinc is an effective adjunct treatment (which is at odds with the following bullet point in line 79).

**Response**: Thank you for highlighting this inconsistency. We agree that the original phrasing overstated the evidence for zinc as an adjunct treatment. We have revised the bullet point to reflect that zinc is a potential rather than established adjunct, in line with the level of evidence presented in the following bullet.

Revised bullet is as follows (page 5; lines 79 – 81):

- *Limited access to effective antibiotics for serious infections in newborns underscores the importance of evaluating inexpensive and potentially beneficial adjunct treatments such as zinc in low-resource settings.*

**Comment:** Line 89: Please mention that Zn or placebo was added to SOC.

**Response**: We have revised the sentence to specify that zinc or placebo was given in addition to the standard of care (page 5; lines 89 – 90).

**INTRODUCTION:**

**Comment:** Please have a look at the first 2 lines; these are currently almost identical to the first lines of the Abstract.

**Response**: We have revised the first two lines of the Introduction to reduce overlap with the Abstract while preserving the essential background on neonatal mortality and its leading causes. The revised version also improves the flow and avoids unnecessary repetition.

The modified first two lines of the Introduction are as follows (page 7; lines 106 – 108):

*Neonatal mortality remains a major global health concern, with the highest burden in sub-Saharan Africa and South Asia [1]. Of the estimated 2.3 million neonatal deaths each year [1], a significant proportion is attributed to severe infections, including sepsis [1–8].*

**Comment:** Line 123: consider stating 'suspected sepsis' rather than 'sepsis'.

**Response**: We agree that ‘suspected sepsis’ is more appropriate in this context and have revised the text accordingly to reflect this distinction (page 7; line 126; Abstract page 3; line 43).

**Comment:** Please provide a definition of clinical severe infection in the introduction, and clarify that this is suspected, not microbiologically confirmed.  Clarify whether this is a conventional clinical term and/or whether this is used in India and Nepal.

**Response**: Thank you for this helpful comment. To improve clarity and narrative flow, we have now revised the final sentence of the Introduction to refer to suspected sepsis, defined as clinical severe infection (CSI) adapted from WHO Integrated Management of Childhood Illness (IMCI) guidelines. Additional details on the definition and criteria for CSI have been provided in the Methods section (page 9; lines 160 – 164). We have also clarified that CSI reflects suspected, not microbiologically confirmed, sepsis. While not country-specific, this case definition is widely used in low- and middle-income settings, including in India and Nepal.

We have revised the last sentence of the Introduction as follows (page 7; lines 125 – 128):

*We conducted a multicenter RCT to estimate the efficacy of adjunct oral zinc in reducing case fatality among young infants aged 3 to 59 days with suspected sepsis, defined as clinical severe infection (CSI) adapted from WHO Integrated Management of Childhood Illness (IMCI) guidelines [21–23].*

The updated Methods section reads as follows (page 9; lines 160 – 165):

*All infants aged 3–59 days visiting the hospitals’ emergency units and having at least one of five signs of CSI (stopped feeding well, severe chest indrawing, axillary temperature >38.0⁰C or <35.5⁰C and movement only when stimulated) [21–23], were screened for eligibility. This definition is adapted from the WHO Integrated Management of Childhood Illness (IMCI) guidelines and reflects suspected, not microbiologically confirmed, sepsis.*

**METHODS:**

**Comment:** line 175: do you mean 'sterile' water with 'clean water'? Please specify.

**Response**: Thank you for pointing this out. We have corrected the text to specify that sterile water or breast milk was used to dissolve the dispersible zinc or placebo tablet (page 10; line 189).

**Comment:** Line 202: you state that Plasma zinc concentrations at baseline and discharge were measured, and I see these are reported in S3. However, please also provide us with the raw data for this in a supplemental file.

**Response**: We have provided the raw data on plasma zinc concentrations at baseline and discharge in a supplemental file in .csv format, as requested.

**Comment:** Please provide more details about the standard of care at each of the different study sites.

**Response**: We have now included a detailed description of standard case management across study sites in the Methods – Procedures section. Although minor contextual differences existed, all hospitals followed harmonized protocols with a fixed antibiotic treatment algorithm, supportive care measures, and physician-led clinical oversight.

The revised text outlines the standard of care for infants with suspected sepsis at the different study sites (pages 10 – 11; lines 198 – 206).

*Antibiotic therapy followed harmonized, site-specific protocols. First-line treatment included intravenous ampicillin or amoxicillin-clavulanic acid with an aminoglycoside (amikacin or gentamicin). Infants previously treated with injectable antibiotics received a third-generation cephalosporin plus aminoglycoside. Suspected meningitis was managed with cefotaxime or ceftriaxone and amikacin, and staphylococcal infections with cloxacillin or amoxicillin-clavulanic acid; vancomycin was added for suspected staphylococcal meningitis. Antibiotics were given for 7–10 days, extended up to 3 weeks for meningitis. Supportive care (fluids, temperature control, and oxygen) was provided as needed. Treating physicians managed all medical care [21].*

**DISCUSSION:**

**Comment:** Please include a discussion on the potential differences in SOC across the study sites. This needs to include potential heterogeneity across hospitals, and should comment on adherence of pill taking once patients and caregivers returned home.

**Response**: We appreciate this valuable comment. We acknowledge that heterogeneity in the standard of care across hospitals is an inherent challenge in multi-site studies such as ours, and that the main implication is effect modification, which may be overlooked and is difficult to fully account for. We have added text to the Discussion in two parts to reflect this and have clarified that adherence to the intervention after discharge was assessed and described in the Results section.

The revised Discussion sections read as

(page 22; lines 378 – 382):

*While standard of care may vary across hospitals, protocol-driven antibiotic management and site-specific training minimized inter-site differences. This variability, however, reflects real-world conditions and enhances the generalizability of our findings. In addition, adherence to post-discharge zinc or placebo was closely monitored and documented through pill counts and caregiver reports.*

(page 22; lines 395 – 400):

*It is important to acknowledge the heterogeneity among study participants, arising from differences in illness severity, nutritional status, and the standard of care across hospitals, as a potential limitation in interpreting the findings. While we evaluated the influence of these factors on the effect of zinc (Figure 3), it is possible that other relevant effect modifiers were not captured. Furthermore, the considerable overlap in confidence intervals across subgroups limits the ability to draw definitive conclusions regarding effect modification.*

**Comment:** Line 335: please remove 'precisely estimated' from this sentence.

**Response**: Thank you for the suggestion. We have removed the phrase ‘precisely estimated’ from the sentence as requested.

The revised sentence reads as: *We report a small, and statistically not significant, effect of daily adjunct zinc therapy on case fatality* (page 21; lines 359 – 360).

**Comment:** Please ensure that discussion of the study is objective.

**Response:** We have reviewed the Discussion carefully to ensure that the language remains objective and evidence-focused. The text describes the results without overstating the effect of zinc and uses appropriately cautious terms when interpreting subgroup and post hoc findings. The strengths and limitations of the study are presented in a balanced manner. No changes were made, as the current version maintains a neutral and scientifically appropriate tone throughout.

**Comment:** Please temper any conclusions related to post hoc analyses.

**Response**: Thank you for this helpful comment. We agree with the need to interpret post hoc findings with caution. The concluding paragraph has been reviewed and already incorporates this by clearly stating that the subgroup findings are exploratory, the confidence intervals are wide, and that further trials are needed. No changes were made as the current wording appropriately reflects the limitations of the post hoc analyses.

The concluding para reads as follows (page 23; lines 406 – 411):

*In conclusion, in this study, the observed effects of zinc were small and did not reach statistical significance. While our prespecified and post-hoc subgroup analyses may suggest a potential beneficial effect of zinc supplementation in subgroups of infants with laboratory parameters or clinical signs suggestive of more severe illness, these findings are exploratory and require cautious interpretation due to the wide confidence intervals and the absence of any effects on death during the 12-week follow-up period.*

FUNDING STATEMENT:

*The funding statement should include: specific grant numbers, initials of authors who received each award, URLs to sponsors’ websites. Also, please state whether any sponsors or funders (other than the named authors) played any role in study design, data collection and analysis, the decision to publish, or preparation of the manuscript. If they had no role in the research, include this sentence: “The funders had no role in study design, data collection and analysis, decision to publish, or preparation of the manuscript.”

**Response:** We have updated the Financial Disclosure statement to include grant numbers, funding sources with URLs, and author initials associated with each award. A clarification of the funders’ role in the study has been provided under the section ‘Role of the Funder/Sponsor’.

ETHICS AND CONSENT:

*Thank you including the names of the IRBs who provided ethical approval for this study. Please also include IRB approval numbers.

**Response:** We have revised the manuscript to include the IRB approval numbers alongside the names of the ethics committees, as requested. These details are now provided in the Methods section under ‘Study design and participants.’

The updated section reads as follows (page 8; lines 134 – 145):

*Ethical approvals were obtained from the Translational Health Science and Technology Institute (THSTI) Institutional Ethics Committee (Ref: THS 1.8.1/(48)); the Nepal Health and Research Council (Ref: 598); the Regional Committee for Medical and Health Research Ethics in Norway (Ref: 2016/407 REK sør-øst D); and the Institutional Ethics Committees (IECs) of all participating hospitals, including Maulana Azad Medical College and Lok Nayak Hospital (Ref: F.1/IEC/MAMC/(54/03/2016/No/137), Vardhman Mahavir Medical College & Safdarjung Hospital (Ref: IEC/VMMC/SJH/Project/May/2016/207), Chacha Nehru Bal Chikitsalaya (Ref: F.1/IEC/MAMC/(53/2/2016/No134), Kasturba Hospital (Ref: 1770/MS/KH/16), Lady Hardinge Medical College and Associated Hospitals (Ref: LHMC/ECHR/2019/14), Kanti Children’s Hospital (Ref: 192/72/73), Institute of Medicine, Tribhuvan University (Ref: 251(6-11-E)^2^/073/074), and Patan Academy of Health Sciences (Ref: out1610281110).*

FIGURES:

* Please provide titles and legends for all figures and tables (including those in Supporting Information files). Please define all acronyms used in each figure or table in its corresponding legend. Please ensure that you include a title for each figure, and include the description of each subpanel in the legend. Specifically this needs to be done for Figure 3.

**Response:** Thank you. Titles and legends for all figures and tables have been provided and all abbreviations have used in each figure/table have been defined at first use.

RCT:

*Thank you for completing the CONSORT 2025 checklist and including it as supporting information. Please cite the checklist in the article (e.g. as ‘S1 Checklist’).

**Response:** We have cited the CONSORT 2025 checklist in the manuscript as “S1 Checklist,” in accordance with the journal’s guidelines (page 8; line 151).

* As your trial had to undergo important modifications in response to extenuating circumstances, please complete the CONSERVE-CONSORT checklist and provide in your Supporting Information.

When completing the checklist, please use section and paragraph numbers, rather than page numbers.

**Response:** We have completed the CONSERVE-CONSORT checklist as requested, referencing relevant section and paragraph numbers. As noted in our response to reviewer comments dated 12 June 2025, our trial did not undergo major modifications due to extenuating circumstances such as the COVID-19 pandemic. The only operational impact was a temporary suspension of recruitment at most sites, which consequently limited the number of eligible participants. This has been acknowledged in the Discussion section under study limitations.

**COMMENTS FROM REVIEWERS:**

**Reviewer #1:** I have read the authors' responses to the 3 reviewer comments and find them acceptable. After reading the full revised text I have no further comments to add.

**Response:** We thank the reviewer for taking the time to review the revised manuscript and responses. We appreciate the positive feedback and are pleased that the revisions have been found acceptable.

**Reviewer #3:** Thank you to the authors for addressing my previous comments well. On their last response regarding interaction testing: I would personally recommend that the discussion, if it mentions interpretations from subgroup analysis, should caveat it my stating that was not significant evidence for there being an interaction. Other than this, I am happy with the revised paper.

**Response:** We appreciate the reviewer’s recommendation. The concluding paragraph of the Discussion already addresses subgroup analyses in a clearly exploratory context and highlights key limitations, including wide confidence intervals, the post hoc nature of the analyses, and the need for further trials. We agree that we can be more explicit about the (lack of) evidence for effect modification and have added clarifying language to the paragraph discussing heterogeneity. We also believe that the confidence intervals in the subgroup forest plots (Figure 3, S1 and S2 Figs) provide a more informative depiction of the precision of subgroup effects than formal interaction p-values. However, if deemed necessary, we would be happy to add a specific statement to the Results section noting that no statistically significant interactions were observed.

Any attachments provided with reviews can be seen via the following link:

_____________________________________________

In compliance with data protection regulations, you may request that we remove your personal registration details at any time.  (Use the following URL: https://www.editorialmanager.com/pmedicine/login.asp?a=r). Please contact the publication office if you have any questions.
