# Supplementary material for: Zinc as adjunct treatment for clinical severe infection in young infants: A randomized double-blind placebo-controlled trial in India and Nepal
Source: PLoS Med. 2025 Oct 9;22(10):e1004759. doi: 10.1371/journal.pmed.1004759 (PMC12527131; doi:10.1371/journal.pmed.1004759)
Supplement: Attachment — Submitted filename: Zn-PLOS Medicine_Response to Editor_31Aug2025.docx [file pmed.1004759.s016.docx]

**Zn Sepsis-PLOS Medicine-Responses to Editor and Reviewer Comments**

Dear Editor**,**

Thank you for your comments. We have carefully addressed each of the points raised and provide a point-by-point response detailing our revisions. A revised version of the manuscript, as well as a marked-up copy highlighting the changes, have been submitted for your review. We believe these revisions have further enhanced the clarity and overall quality of our paper.

Best regards
Nitya and Tor

------------------------------------------------------------

**Comment:** Discussion: you mention the study has ‘notable strengths’. Please signpost the limitations in the same clear way.

**Response:** Thank you for this suggestion. In the Discussion section, we have now clearly signposted the study’s limitations in a separate paragraph, immediately following the strengths. This section explicitly outlines the main limitations of our study, making them as clear and accessible as our summary of strengths.

**Comment:** Please include URLs for the funding agencies in your financial disclosure.

**Response:** Thank you for bringing this to our attention. We have revised the Financial Disclosure section to include the URLs for each of the funding agencies as requested.

**Comment:** Title and legends:
- For Figure 1: please change your title to “study flowchart”

**Response:** Following your recommendation, the title of Figure 1 has been changed to “Study Flowchart.”

-For Figure 2 and 3: Please provide a title for each figure, and a legend, which includes the description of each subpanel. (Currently, there are separate titles for the subpanels)

**Response:** Thank you for highlighting this. We have revised Figures 2 and 3 to include a single overall title for each figure and have provided a comprehensive legend that describes each subpanel within the figure, as per your guidance.

**Comment:** CONSERVE-checklist: Thanks for providing the CONSERVE-checklist. Can you please also provide a citation to this checklist within the manuscript (similar to the “S1 checklist”)?

**Response:** We have now included a citation to the CONSERVE checklist within the manuscript, in the same manner as the “S1 checklist,” to ensure appropriate reference to the supplementary material. The CONSERVE checklist is cited as “S2 Checklist.”

**Comment:** Thank you for providing us with the raw data for the plasma zinc concentrations at baseline and discharge. Could you please upload these as supplemental data, and state in the legend of figure S3 where these data can be found? We suggest the following statement “The data underlying the graphs shown in the figure can be found in S1 data”.

**Response**: The raw data for plasma zinc concentrations at baseline and discharge have now been uploaded as supplemental data (S1 Data). We have updated the legend of S3 Table to indicate that the data underlying the values shown in the table can be found in S1 Data, following your suggested wording.
